# Strategies to target SARS-CoV-2 entry and infection using dual mechanisms of inhibition by acidification inhibitors

**DOI:** 10.1371/journal.ppat.1009706

**Published:** 2021-07-12

**Authors:** Chaitra Prabhakara, Rashmi Godbole, Parijat Sil, Sowmya Jahnavi, Shah-e-Jahan Gulzar, Thomas S. van Zanten, Dhruv Sheth, Neeraja Subhash, Anchal Chandra, Akshatha Shivaraj, Patricia Panikulam, Ibrahim U, Vijay Kumar Nuthakki, Theja Parassini Puthiyapurayil, Riyaz Ahmed, Ashaq Hussain Najar, Sai Manoz Lingamallu, Snigdhadev Das, Bhagyashri Mahajan, Praveen Vemula, Sandip B. Bharate, Parvinder Pal Singh, Ram Vishwakarma, Arjun Guha, Varadharajan Sundaramurthy, Satyajit Mayor

**Affiliations:** 1 National Centre for Biological Sciences (TIFR), Bengaluru, India; 2 University of Trans-Disciplinary Health Sciences and Technology (TDU), Bengaluru, India; 3 SASTRA University, Thanjavur, India; 4 CSIR—Indian Institute of Integrative Medicine, Jammu, India; 5 Institute for Stem Cell Science and Regenerative Medicine (inSTEM), Bengaluru, India; 6 Manipal Academy of Higher Education (MAHE), Madhav Nagar, Manipal, Karnataka, India; Icahn School of Medicine at Mount Sinai, UNITED STATES

## Abstract

Many viruses utilize the host endo-lysosomal network for infection. Tracing the endocytic itinerary of SARS-CoV-2 can provide insights into viral trafficking and aid in designing new therapeutic strategies. Here, we demonstrate that the receptor binding domain (RBD) of SARS-CoV-2 spike protein is internalized via the pH-dependent CLIC/GEEC (CG) endocytic pathway in human gastric-adenocarcinoma (AGS) cells expressing undetectable levels of ACE2. Ectopic expression of ACE2 (AGS-ACE2) results in RBD traffic via both CG and clathrin-mediated endocytosis. Endosomal acidification inhibitors like BafilomycinA1 and NH_4_Cl, which inhibit the CG pathway, reduce the uptake of RBD and impede Spike-pseudoviral infection in both AGS and AGS-ACE2 cells. The inhibition by BafilomycinA1 was found to be distinct from Chloroquine which neither affects RBD uptake nor alters endosomal pH, yet attenuates Spike-pseudovirus entry. By screening a subset of FDA-approved inhibitors for functionality similar to BafilomycinA1, we identified Niclosamide as a SARS-CoV-2 entry inhibitor. Further validation using a clinical isolate of SARS-CoV-2 in AGS-ACE2 and Vero cells confirmed its antiviral effect. We propose that Niclosamide, and other drugs which neutralize endosomal pH as well as inhibit the endocytic uptake, could provide broader applicability in subverting infection of viruses entering host cells via a pH-dependent endocytic pathway.

## Author summary

This study investigates the cellular mechanisms by which SARS-CoV-2 can gain entry into human cells. We find that the virus employs diverse endocytic processes to enter cells and the acidic environment within these endocytic compartments is essential for infection. Using these observations from first principles, we screened a small set of FDA-approved drugs which could potentially inhibit endosomal acidification and therefore prevent viral entry and infection. The routinely prescribed anti-helminthic drug, Niclosamide, was observed to have this capability. Our study proposes that drugs altering both endocytic entry as well as endosomal acidification can assist in the clinical management of viral infections.

## Introduction

Coronaviruses (CoVs) are a group of related enveloped RNA viruses of which some are known to cause respiratory tract infections in humans. The recent emergence of SARS-CoV-2 and its rapid spread across the world in multiple waves has posed a global health emergency [1]. Several therapeutic strategies are currently being used to alleviate the respiratory symptoms of patients infected with SARS-CoV-2 [2,3]. However, strategies aimed to directly counter the virus have met with only limited success. A search for antivirals affecting the endocytic entry of viruses is particularly useful as infections from multiple emerging CoV-2 strains and other related viruses can be controlled through the inhibition of a common step [4].

Virus entry into host cells is a multistep process. A key step in successful invasion is the release of viral genomic content into the host cell cytoplasm. To achieve this, viruses bind to specific cell surface receptors and subsequently undergo membrane fusion either directly at the plasma membrane or following endocytic uptake. While fusion directly at the plasma membrane is well established for HIV and Influenza virus infections [5,6], both alternatives of entry are feasible for CoV infections depending on the availability of receptors and proteases at the host cell surface. Different CoVs interact with a range of specific receptors for entry [7–11]. Although angiotensin converting enzyme 2 (ACE2) is a well-studied receptor for SARS-CoV-2 [12], other receptors and co-receptors are being discovered [13–17]. Additionally, CoVs require proteolytic processing of the viral envelope Spike protein by host cell proteases to gain entry [18,19]. Therefore, these viruses can directly fuse at the cell surface if the Spike protein is cleaved by a cell surface serine protease like TMPRSS2 [12,20], or utilize an endo-lysosomal route for fusion, where the Spike protein is primed by cysteine protease cathepsins [12,21–23]. Viral entry and infection in different host cells is dependent on the expression of these key host factors [24]. Cell tropism studies revealed that SARS-CoV-2 infection is not only restricted to airway epithelium but the cells along the Gastrointestinal tract are also infected [25]. An understanding of the entry pathways across various host cell types is important as it allows better interpretation of cell-based drug screens and translatability of cellular models of infection.

The role of the endo-lysosomal network appears to be crucial in delivering viruses to acidic compartments. For instance, cathepsins function optimally in a low pH environment [18,26]. Inhibitors of acidification which increase the pH of endosomal compartments significantly reduce the infection of pseudoviruses as well as native MERS-CoV, SARS-CoV, SARS-CoV-2 viruses [12,27–30]. Drugs like Apilimod and YM201636 reduce CoV infections [27,31,32] by inhibiting the maturation of late endosome to lysosome without altering the endosomal pH directly. These studies emphasize the importance of an optimal endocytic network in viral entry and infection. The applicability of therapeutics that act by dissipating pH gradients across intracellular compartments remains to be explored in the clinical management of SARS-CoV-2 infection.

Multiple endocytic pathways operate at the cell surface [33,34] which can be exploited by viruses. However, the endocytic routes preferred by SARS-CoV-2 in different host cell types is largely unknown. The clathrin and dynamin independent CLIC/GEEC (CG) endocytic pathway [35], is of particular interest here as uptake through this pathway is known to be pH-dependent. Vacuolar ATPases (V-ATPases), which actively pump protons into the endocytic compartments [36], play a crucial role in the formation of CG endosomes as established using genetic and pharmacological perturbations [37,38]. By contrast, uptake through clathrin-mediated endocytosis (CME) remains unaltered upon V-ATPase perturbation [37]. Homotypic fusion of nascent CG endosomes (called CLICs–clathrin-independent carriers) forms highly acidic early endosomal compartments of the CG pathway (called GEECs–GPI anchored protein enriched early endosomal compartments) with an estimated luminal pH of 6.0 [39]. Thus, GEECs could provide a conducive environment for viral uncoating and membrane fusion. Interestingly, Adeno-associated virus (AAV2) hijacks the CG pathway for infection [40] and SARS-CoV has also been reported to enter cells through a clathrin and dynamin independent endocytic pathway [29]. These observations prompted us to study the role of CG endocytosis in the context of SARS-CoV-2 entry and infection.

In this report, we study the endocytosis of receptor binding domain (RBD) of SARS-CoV-2 Spike protein in gastric epithelial cells (AGS) in the presence and absence of ACE2. We show that RBD is endocytosed via the CG pathway and its uptake is sensitive to pharmacological perturbations of this pathway in AGS cells which express undetectable levels of ACE2. Overexpression of ACE2 in AGS cells (AGS-ACE2) results in RBD employing both CG and CME pathways for entry. Endosomal acidification inhibitors such as BafilomycinA1 and NH_4_Cl not only altered the endosomal pH but also blocked RBD uptake, similar to CG cargo uptake. Spike-pseudovirus transduction assays revealed that these acidification inhibitors impede early steps of viral entry. We find that Chloroquine which also blocks early steps of Spike-pseudovirus entry acts differently from BafilomycinA1 and NH_4_Cl by not altering the RBD uptake and minimally changing the endocytic pH. Extending our observations, we conducted a targeted screen using a subset of FDA-approved drugs for similar cell biological mechanisms of inhibition as offered by BafilomycinA1 and NH_4_Cl. We identified Niclosamide as a promising candidate that inhibits RBD uptake, increases endosomal pH, impedes Spike-pseudovirus infection and potentiates the effects of Hydroxychloroquine. Using a clinical isolate of SARS-CoV-2, we validated the antiviral effects of BafilomycinA1 and Niclosamide in Vero as well as AGS-ACE2 cells, thus underlining the potential of endosomal acidification inhibitors for antiviral therapy. Along with the recently described anti-inflammatory property of Niclosamide [41,42], we suggest that this drug could be a feasible start point for developing small molecule entry inhibitors to mitigate SARS-CoV-2 infection.

## Results

### Generation of SARS-CoV-2 probe to study its endocytosis itinerary

**Fig 1:**
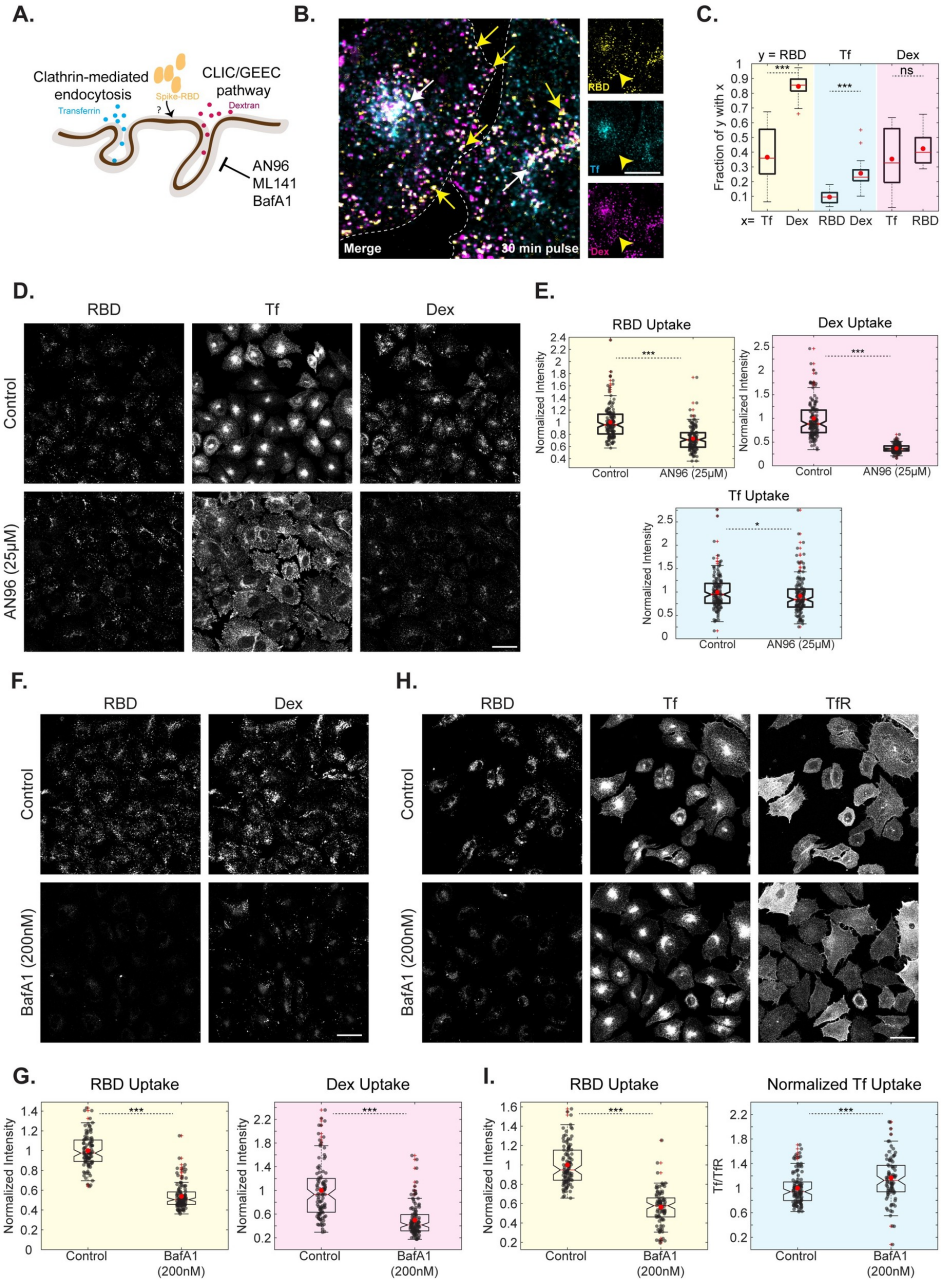
RBD uptake is sensitive to CG Pathway inhibitors in AGS cells. A: Schematic describing endocytic pathways at the plasma membrane with specific cargoes for each endocytic pathway: transferrin (CME Cargo) and 10kDa dextran (CG Cargo). AN96, ML141 and BafA1 specifically affect the uptake of CG cargoes. B, C: AGS cells were pulsed with RBD, dextran and transferrin for 30 minutes and imaged at high resolution after fixation. Images are shown in B and quantification of Manders’ co-occurrence coefficient is shown in C. This compares the fraction of RBD endosomal intensity with transferrin or dextran (p-value < e-06), transferrin endosomal intensity with dextran or RBD (p-value < e-05) and dextran endosomal intensity with transferrin or RBD (p-value = 0.18). RBD is more co-localized to dextran endosomes. Number of cells = 10. White arrow represents endosomes containing RBD, dextran and transferrin. Yellow arrow represents endosomes with RBD and dextran without transferrin. Dashed white line in B represents the approximate cell boundary. D, E: AGS cells were pretreated with Control (0.6% DMSO) or AN96 25μM for 30 minutes and pulsed with RBD, dextran and transferrin for 30 minutes with or without the inhibitor. Treatment with AN96 reduces RBD (p-value < e-19) and dextran (p < e-44) uptake while minimally alters transferrin uptake (p = 0.02). Images are shown in D and quantification in E. Numbers of cells > 100 for each treatment. F, G: AGS cells were treated with Control (0.2% DMSO) or BafA1 200nM for 30 minutes and pulsed with RBD and dextran for 30 minutes with or without the inhibitor. Treatment with BafA1 reduces RBD (p-value < e-33) and dextran (p-value < e-18) uptake. Images are shown in F and quantification in G. Numbers of cells > 100 for each treatment. H, I: AGS cells were treated with Control (0.2% DMSO) or BafA1 200nM for 30 minutes and pulsed with RBD and transferrin for 30 minutes with or without the inhibitor. The surface transferrin receptor (TfR) was labelled after fixation. Treatment with BafA1 reduces RBD uptake (p-value < e-27) and increased normalized transferrin uptake (p-value < e-03). Images are shown in H and quantification in I. Numbers of cells > 80 for each treatment. Data (E, G, I) is represented as a scatter with box plot. Black dots represent per-cell data points. Box plot represents the distribution (25% to 75% percentile) with the red line indicating the median and red dot indicating the mean of the distribution. Whiskers represent distribution up to 1.5 times interquartile range and + indicates outliers beyond the whiskers. In the entire manuscript, ***, **, * and ns indicate p-value of Wilcoxon rank-sum test < 0.001, <0.01, <0.05 and not significant, respectively. Scale bar: 20μm (B) and 40μm (D, F, H).

Viruses can enter cells via multiple endocytic routes (Fig 1A) [33]. A cellular system that exhibits clathrin-dependent and independent routes is required for identifying the possible pathways that the virus may utilize to enter cells. Therefore, we chose a human adenocarcinoma gastric cell line (AGS cells) [43] as a surrogate model system to study SARS-CoV-2 endocytosis and infection. To explore the trafficking itinerary, we purified the receptor binding domain (RBD) of SARS-CoV-2 Spike protein [44] and fluorescently labelled the same (S1A and S1B Fig and Materials and Methods). We tested the specificity of the labelled RBD probe in AGS cells transiently overexpressing myc-tagged ACE2 and found that more RBD was bound to cells overexpressing ACE2 (S1C Fig). We also observed a positive correlation between the amount of RBD endocytosed and surface levels of ACE2 (S1D and S1E Fig), supporting the notion that ACE2 is one of the cell surface receptors of RBD [12].

### RBD is internalized via CG endocytosis and RBD uptake is sensitive to CG Pathway inhibitors

We employed the methodology of tracking RBD uptake along with cargoes specific to CME (transferrin) and CG (10kDa dextran) endocytic pathways to determine the endocytic route taken up by RBD (Fig 1A). At 10 minutes post internalization, transferrin endosomes of the CME pathway are distinct from dextran endosomes of the CG pathway [43]. At these times, internalized RBD is colocalized with endosomes containing the CG cargo but not the CME cargo (S2A and S2B Fig). At 30 minutes post internalization, as well, this segregation remains. While a small fraction of RBD endosomes were colocalized with endosomes containing both transferrin and dextran, a large fraction of RBD endosomes were localized to compartments uniquely marked by dextran (Fig 1B and 1C; compare % RBD with transferrin and dextran). This suggests that the itinerary of uptake of RBD is similar to CG cargo and different from CME cargo.

CG pathway is regulated by small GTPases—CDC42 [45], Arf1 [46] and GEF of Arf1, GBF1 [47]. Inhibitors that block the function of these regulators affect the formation of CG endosomes without altering uptake through the CME pathway. The inhibitor AN96, which is a stable analog of LG-186 [48,49], targets GBF1 and specifically affects the CG pathway (Godbole et al., Manuscript in preparation). Towards determining the trafficking route of RBD, we examined the effect of inhibitors of CG pathway on RBD, dextran and transferrin uptake in AGS cells. Cells were subjected to a brief pre-treatment with different inhibitors (30 minutes), followed by a pulse of RBD, dextran and transferrin (30 minutes) in the presence of these inhibitors (Materials and Methods). We observed that AN96 treatment reduced both RBD and dextran uptake but had minimal effects on the amount of transferrin internalized (Fig 1D and 1E). We also observed that the peri-nuclear transferrin recycling endosomal pool was redistributed throughout the cytoplasm upon treatment with AN96 without affecting the net amount of transferrin internalized. Another CG pathway inhibitor, ML141 (CDC42 inhibitor) [49], also significantly decreased both dextran as well as RBD uptake (S2E and S2F Fig).

Several viruses utilize the macropinocytosis pathway as an entry route into cells [50]. Since 10kDa dextran marks both CG cargo as well as larger endocytic compartments like those derived from macropinocytosis [51], we tested if macropinocytosis plays any role in RBD uptake. Macropinocytosis is dependent on amiloride-sensitive Na+/H+ exchangers [52]. Upon treatment with Amiloride, we found no alteration in the uptake of RBD, dextran and transferrin (S2G and S2H Fig). Whereas macropinocytic dextran uptake stimulated by PMA (phorbol 12-myristate 13-acetate) was completely rescued upon co-treatment with Amiloride (S2I Fig). This confirms that macropinocytosis does not play a role in RBD trafficking in AGS cells. Together, the co-localization studies and pharmacological inhibition experiments strongly suggest that RBD uptake occurs via the CG pathway and is inhibited by specific blockers of the CG pathway.

### BafilomycinA1 and NH_4_Cl block RBD uptake

Given the relevance of acidification in both formation of CG endosomes [37,39] and viral infection [53], we focused on studying the role of acidification inhibitors on the uptake of RBD. We assessed the effect of BafilomycinA1 (BafA1), a specific inhibitor of V-ATPase [54], on RBD, dextran and transferrin uptake in AGS cells. A strong reduction in RBD and dextran uptake and an increase in normalized transferrin uptake was observed when cells were treated with BafA1 (Fig 1F–1I), in a dose-dependent manner (S3A and S3Bi Fig). The increase in transferrin uptake could be because BafA1 also retards the transferrin recycling from the recycling endosomes [55] and thereby increasing the net amount of transferrin internalized within cells as observed. We also examined the effect of NH_4_Cl, a weak base known to alter endosomal acidification [56], on the uptake of these 3 cargoes. Similar results as with BafA1 were observed (S3A and S3Bi Fig), confirming our earlier [37] finding that uptake via the CG pathway is pH sensitive and blocking acidification results in reduced CG uptake.

Towards understanding the mechanism of action for acidification inhibitors in bringing about these changes in trafficking, we assessed their effect on two parameters–numbers of endosomes (S3Bii Fig) and per-endosome intensity in the presence/absence of inhibitor (S3Biii Fig). We observed that both BafA1 and NH_4_Cl reduced the total number of RBD and dextran endosomes without affecting the per-endosome intensity. However, while the total number of transferrin endosomes remained unchanged, the per-endosome intensity of transferrin increased with BafA1 and NH_4_Cl treatment. This indicates that the reduction in RBD and dextran is likely due to a block in the entry while an increase in per-endosome transferrin intensity could be because of a block in the formation of recycling endosome carriers, as proposed earlier.

These results are not specific to AGS cells alone. HEK-293T cells, which are also permissive to Spike-pseudovirus transduction, showed similar inhibition of RBD and dextran uptake, and an increase in transferrin uptake with BafA1 (S10A–S10D Fig).

### RBD is localized to acidic compartments

**Fig 2:**
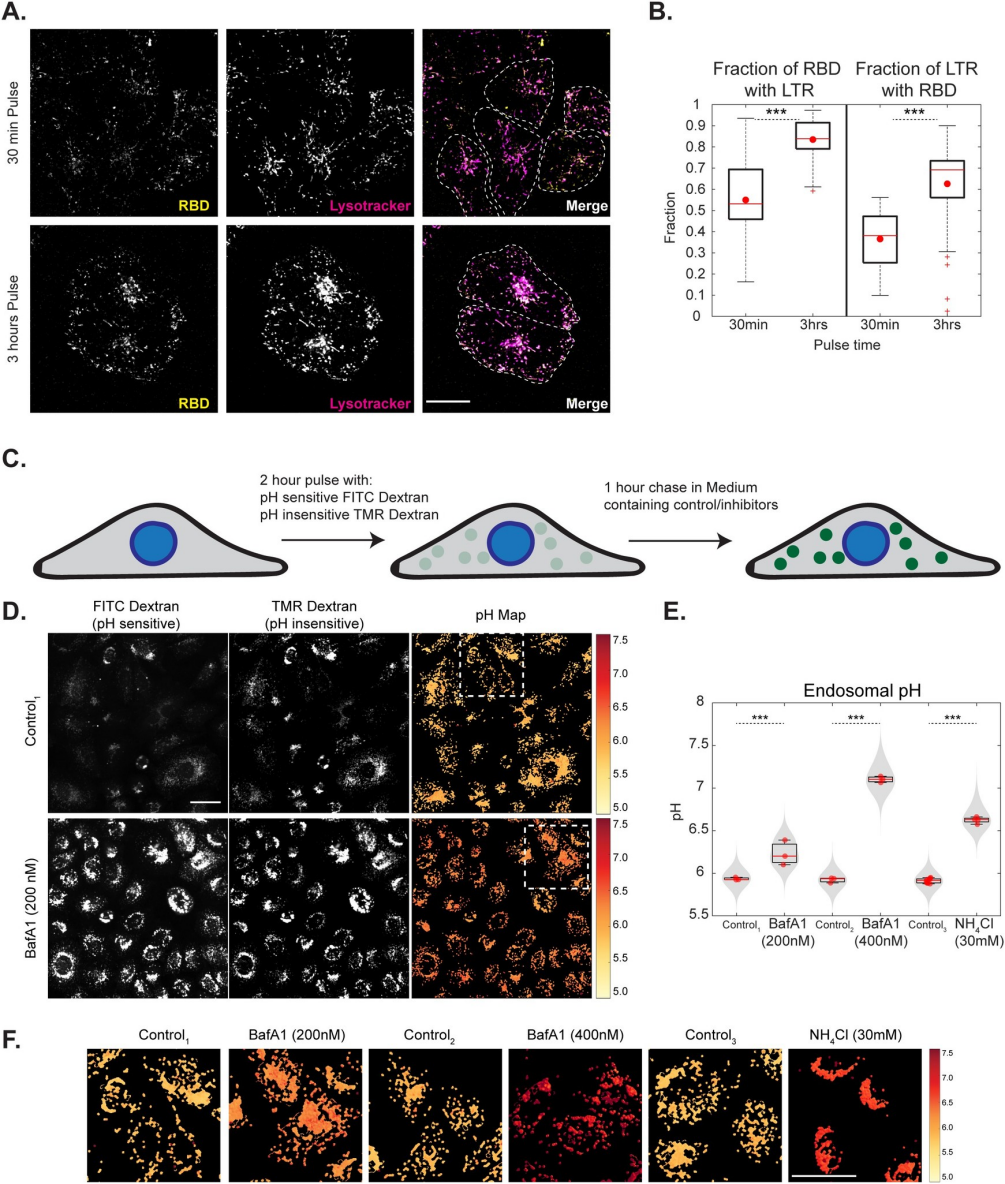
RBD trafficking with CG cargo is localized to acidic compartments and endosomal acidification inhibitors neutralize these endosomes. A, B: AGS cells were pulsed with RBD for 30 mins or 3 hours, labelled with lysotracker in the last 15 minutes of pulse and imaged live at high resolution. Images are shown in A and quantification of Manders’ co-occurrence coefficient is shown in B. RBD is colocalized with Lysotracker positive compartments. With increase in time more RBD is associated with Lysostracker (p-value < e-06) and more Lysotracker positive compartments have RBD (p-value < e-06). Each condition has >12 cells. Dashed white line in A represents approximate cell boundary. C: Schematic describing the experimental protocol for estimating the pH of endosomes by ratiometric measurements using pH-sensitive (FITC) and pH-insensitive (TMR) dextran. D-F: AGS cells were pulsed with FITC and TMR dextran for 2 hours, chased for 1 hour with BafA1 200nM/400nM, NH_4_Cl 30mM or control and imaged live. Endosomal pH is increased upon addition of acidification inhibitors (p-values < e-118 for BafA1 200nM, < e-122 for BafA1 400nM, < e-223 for NH_4_Cl). Images along with pH maps are shown in D (and in S4C) and quantification in E (and in S4D). Enlarged regions of pH maps indicated by white boxes are shown in F. Box plot in E represents the distribution of medians of each repeat which is denoted by red dots. Violin plot indicates all the data points from repeats. Colour bar in F corresponds to the estimated endosomal pH. Control_1_ is 0.2% DMSO, Control_2_ is 0.4% DMSO and Control_3_ is 0% DMSO. Number of repeats ≥ 3 for each treatment and each repeat has >80 cells. Scale bar: 20μm (A) and 40μm (D).

Internalized cargoes can be recycled along with the bulk membrane [57] or directed towards degradation with the fluid phase [58]. Typically, transferrin bound to its receptor marks the early sorting/recycling endosomes and lysotracker labels the acidic degradative compartments within a cell [34]. At 30 minutes of pulse with the three cargoes, while a small fraction of RBD (~36%) associated with transferrin, the majority of RBD (~84%) co-localized with dextran suggesting that RBD is directed predominantly towards the degradation route rather than the recycling route (Fig 1B and 1C). The lysotracker labelling showed highly acidic tubular compartments with significant co-localization with RBD. At 30 minutes of pulse with RBD, around 55% of RBD co-localized with lysotracker marked compartments. At longer time points (3 hours) of pulse with RBD, an even increased proportion of RBD (85%) associated with compartments marked by lysotracker, confirming that RBD is trafficked to acidic compartments (Fig 2A and 2B).

### BafilomycinA1 and NH_4_Cl alter the pH of acidic endosomal compartments

We next focused on determining the change in endosomal pH brought about by various inhibitors within the acidic compartments populated by RBD. Cells were labelled with pH-sensitive (FITC) and pH-insensitive (TMR) dextran for 2 hours and chased for 1 hour with or without inhibitors (Fig 2C and Materials and Methods). The above pulse and chase durations were chosen to allow accumulation of labelled dextran in late endosomes and lysosomal compartments. Additionally, since the acidification inhibitors also have inhibitory roles in the early steps of CG endocytosis as discussed in the previous section, to evaluate their effect on endosomal pH, cells were incubated with inhibitors only during the chase. While the ratio of the fluorescence of these probes is used to estimate endosomal pH by comparing the ratio with the calibration curve [59] (S4A and S4B Fig and Materials and Methods), quantifications of the endosomal intensities and the endosomal number of TMR dextran aids in understanding the effect of various drugs on late endosomal trafficking.

Treatment of cells with acidification inhibitors showed an increase in endosomal pH. The average pH of the late endosomes in control cells was 5.8. The pH of these compartments increased to 6.2 and 7.1 in the presence of BafA1 200nM and 400nM respectively. Incubation with NH_4_Cl also resulted in increasing the pH of these endosomes to 6.6 (Figs 2D and 2E and S4C). While BafA1 marginally changed the TMR intensity per endosome, NH_4_Cl greatly increased the TMR intensity indicating that NH_4_Cl also brings about the fusion of endosomes (S4D Fig). All the acidification inhibitors also reduced the numbers of endosomes (S4D Fig) and this effect was most prominent with NH_4_Cl wherein the endosomes were organized close to the perinuclear region (S4C Fig). The spatial pH maps show the distribution of the pH of endosomes within a cell. Cells treated with BafA1 400nM and NH_4_Cl showed a homogenous distribution of endosomes with increased pH similar to the respective cell averages. On the other hand, cells treated with BafA1 200nM, showed heterogeneity in endosomal pH with some endosomes depicting high pH while others were closer to the average (Fig 2F).

To assess the effect of BafA1 on the pH of early time point endosomes, AGS cells were labelled with FITC and TMR dextran for 20 minutes and chased for 10 minutes with or without BafA1 for the entire duration of pulse and chase (S4E Fig). While the total amount of dextran uptake is not affected significantly, the endosomal FITC intensity and the endosomal ratio of FITC/TMR, which can be considered as a proxy for endosomal pH, show a robust increase with BafA1 treatment (S4F Fig). This indicates that BafA1 also affects the endosomal pH of early time point endosomes.

### Endosomal acidification inhibitors affect the entry of Spike-pseudovirus

The observations made using RBD internalization were extended to SARS-CoV-2 Spike-pseudotyped lentiviral particles (Spike-pseudovirus), generated using a previously established methodology [60]. Pseudoviral infection was assessed using reporter (mCherry) expression (S5A Fig and Materials and Methods). The specificity of the Spike-pseudovirus was validated by comparing infection with an alternatively pseudotyped virus (VSVG; S5Di Fig) and bald pseudoparticles (S5C Fig). Dilutions of the supernatant containing bald pseudoparticles showed no transduction, while similar dilutions of supernatant with Spike-pseudovirus showed high levels of transduction in AGS (S5C Fig). Independently, a competition experiment was conducted to assess the effect of excess soluble RBD and trimeric RBD on transduction of Spike-pseudovirus in AGS (Materials and Methods). The transduction efficiency was reduced in the presence of soluble RBD as well as trimeric RBD, indicating that the Spike-pseudovirus competes for the same binding sites as RBD (S5E Fig).

**Fig 3:**
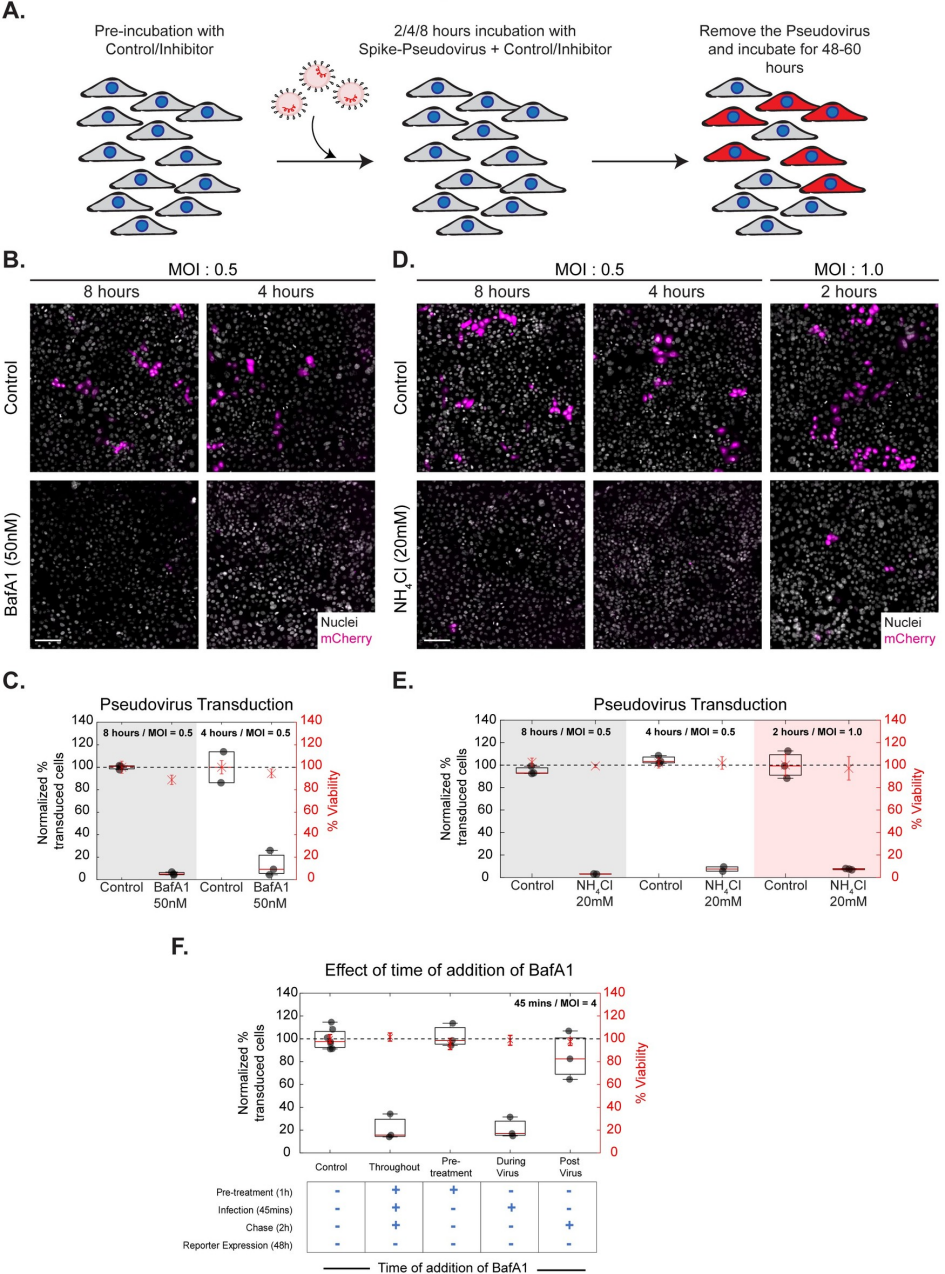
BafA1 and NH_4_Cl affect Spike-pseudovirus infection. A: Schematic describing the experimental protocol for SARS-CoV-2 Spike-pseudovirus transduction assay. AGS cells were pre-incubated with the inhibitors (BafA1 50nM, NH_4_Cl 20mM, CQ 50μM) for an hour and transduced with Spike-pseudovirus at MOI 0.5 or MOI 1.0 for indicated incubation times. Following this, virus and inhibitor-containing medium was removed, and cells were further incubated with inhibitor-free media. B and D show images of AGS cells expressing the reporter mCherry protein and C and E show the quantification of normalized % transduction of cells treated with inhibitors compared to respective controls. B-E: Transduction efficiency is reduced with BafA1 (p-values < e-86 for 8 hours, < e-60 for 4 hours) and NH_4_Cl (p-values < e-83 for 8 hours, < e-72 for 4 hours, < e-85 for 2 hours) at all time points of incubation. Control in B is 0.05%DMSO and in D is 0%DMSO. Number of repeats ≥ 2 for each treatment. F: AGS cells were treated with control or BafA1, pre/during/post incubation with virus as well as throughout all three stages. BafA1 significantly reduces transduction when used throughout (p-value < e-63) as well as during virus presentation (p-value < e-59). Only pre-treatment or post-treatment of BafA1 does not significantly change the percentage of transduction. Number of repeats = 7 for control and 3 for each condition. Data (C, E, F) is represented as percentage transduction normalized to control on left Y-axis along with percentage viability, calculated from the total number of nuclei, normalized to control on the right Y-axis. Black dots represent the mean of each repeat. Box plot represents the distribution of the means (25% to 75% percentile) with red line indicating the median of the distribution. Asterix with error bars in red represents the mean +/- SD of % viability. Black dotted line marks 100%. Scale bar: 100μm (B, D).

Since our experiments were aimed at understanding the entry mechanism of Spike-pseudovirus, we designed the transduction assays with shorter pseudovirus and inhibitor incubation times and followed the infection efficiency by tracing reporter expression at a later time point (Fig 3A and Materials and Methods). We characterized the transduction efficiency of the pseudovirus and found 0.5 MOI (4/8 hours) or 1 MOI (2 hours) of pseudovirus incubation to be optimal (S5F Fig). This design was chosen to reduce the long-term toxicity of the inhibitors to the cells and minimize any secondary effects on the translational processes of the reporter gene post entry.

Spike-pseudovirus infection has been previously reported to be reduced upon treatment with NH_4_Cl, BafA1 and Chloroquine in different cell types [27,61]. Using our methodology, we tested the effect of NH_4_Cl and BafA1 in AGS cells and observed a significant reduction in Spike-pseudovirus infection with no difference in cell viability at all time points of viral incubation (Fig 3B–3E). HEK-293T (S10E–S10G Fig) and A549-ACE2 (S10H Fig) cells also exhibited a similar inhibition of transduction upon treatment with acidification inhibitors.

RBD uptake is reduced upon treatment with AN96 and ML141, albeit to a lesser extent compared to the effect of BafA1 and NH_4_Cl. Therefore, we assessed the effect of AN96 and ML141 on Spike-pseudovirus transduction in AGS cells. Different concentrations of AN-96 were tested, and we observed no reduction in transduction even at the highest concentration of 25μM (S5Gi Fig), without any compromise on cell viability. Similarly, treatment with ML141 did not alter the normalized percentage transduction compared to the control (S5Gii Fig). Blocking of the CG pathway often results in the redistribution of CG cargo towards the CME pathway [43]. Therefore, using high-resolution imaging, we assessed the fate of the RBD endosomes that continued to be internalized upon treatment with AN96. We observed that an increased fraction of internalized RBD endosomes colocalized with transferrin in AN96 treated cells compared to that of control (S2C and S2Di Fig). Similarly, increased co-occurrence was observed in the fraction of dextran endosomes associated with transferrin endosomes on comparing the control with AN96 treated cells (S2Diii Fig). Blocking the CG pathway results in an altered trafficking itinerary of RBD and increases its association with transferrin. This suggests that RBD could be redirected to be internalized via the CME upon blocking the CG pathway. Partial inhibition of uptake may not strongly manifest in our pseudovirus assay, as the read-out is all or none and is not sensitive to the number of virus particles entering the cells. Together with the results of BafA1 and NH_4_Cl, our findings suggest that inhibitors that affect both RBD uptake and neutralize acidic endosomes could be one of the strategies used to impede Spike-pseudovirus transduction. However, those which affect one or the other pathway may not be as effective at blocking virus transduction.

### Chloroquine does not affect RBD uptake, minimally alters endosomal pH but affects Spike pseudovirus infection

**Fig 4:**
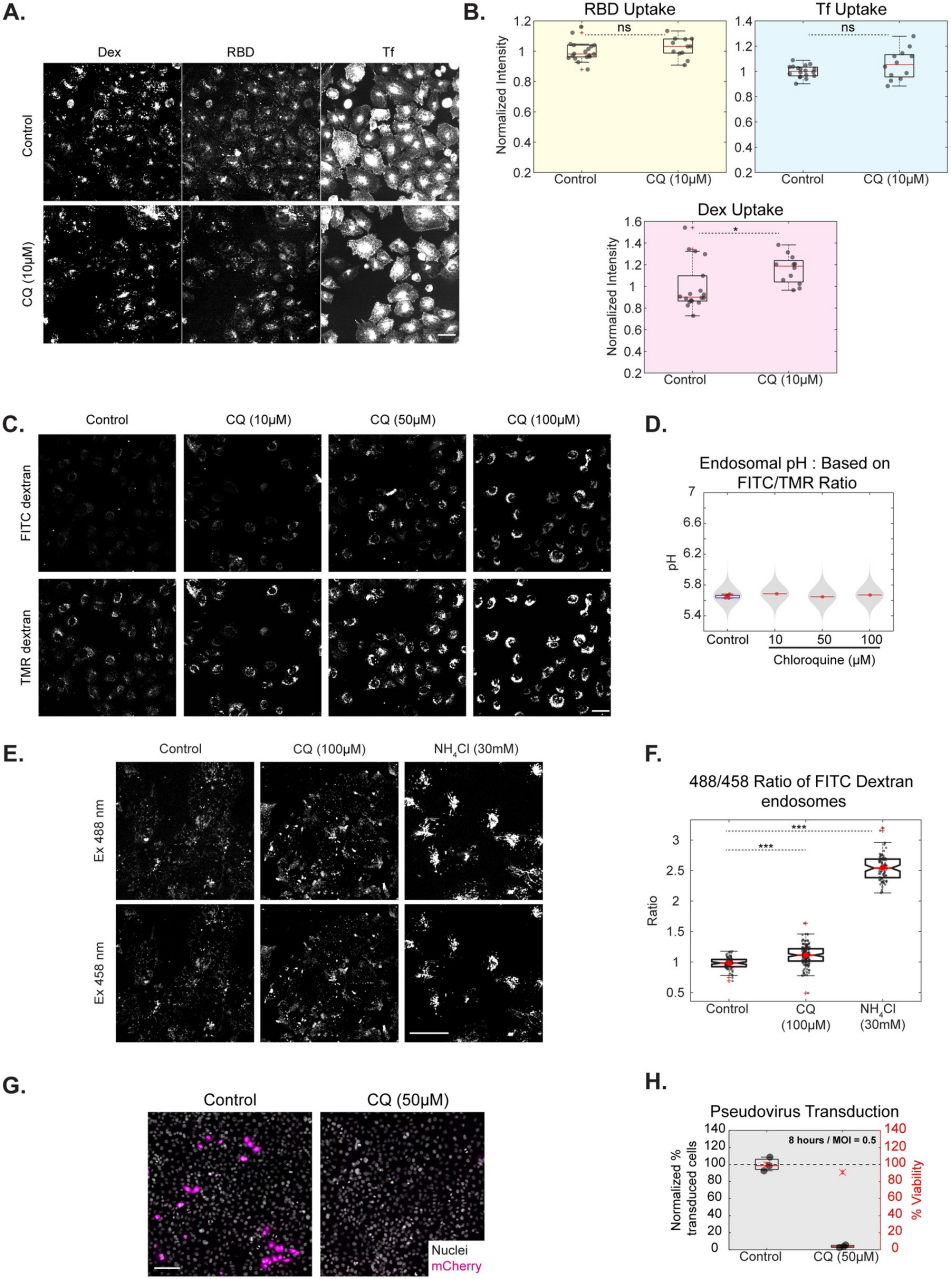
Chloroquine does not affect RBD uptake, minimally alters endosomal pH but affects Spike pseudovirus infection. A, B: AGS cells were treated with Control (0% DMSO) or CQ 10μM for 30 minutes and pulsed with RBD, transferrin and dextran for 30 minutes with or without the inhibitor. Images shown in A and quantification in B show no change in the uptake of transferrin and RBD and marginal change in dextran uptake with CQ (p values = 0.2 for RBD, 0.24 for Tf, 0.013 for Dex). Box plot represents the distribution of medians of each repeat which is denoted by black dots. Number of repeats = 18 and 12 for Control and CQ, respectively and each repeat has >100 cells. C, D: AGS cells were pulsed with FITC and TMR dextran for 2 hours, chased for 1 hour with Control (0.2% DMSO), 10, 50 or 100μM of CQ and imaged live. CQ minimally alters the FITC/TMR ratio (p-value < e-23, 0.38 and < e-03 for 10, 50 and 100 μM of CQ, respectively). Numbers of cells in each condition is >150 cells. E, F: AGS cells pulsed with pH-sensitive (FITC) dextran for 2 hours and chased for 1 hour with Control or 100μM CQ or 15 minutes with 30mM NH_4_Cl and imaged live. CQ increases the 488/458 ratio of FITC dextran only slightly (p < e-10) compared to the increase brought about by NH_4_Cl (p-value < e-27). Images are shown in E and quantification in F. Number of cells > 75 for each treatment. G, H: Assay as described in 3A; Transduction efficiency is reduced with CQ with 8 hours of incubation (p-value < e-90). Number of repeats = 3 each for 0% DMSO Control and CQ. Data representation in B, D is as described in Fig 2; F as described in Fig 1; H as described in Fig 3. Scale bar: 40μm (A, C, E), 100μm (G).

Chloroquine, a diprotic weak base, is expected to accumulate in acidic compartments and neutralize lysosomal pH [62]. While, mounting evidence shows that Chloroquine and its analogs can inhibit the infection by several viruses such as Ebola, Dengue, Chikungunya, HIV, etc [63], many studies point towards differences between the mode of action of Chloroquine and acidification inhibitors–BafA1 and NH_4_Cl [64,65]. We, therefore, tested the effect of Chloroquine on RBD, dextran and transferrin uptake to verify if it behaves like BafA1. We found that upon treatment with Chloroquine, the uptake of neither RBD nor transferrin was altered significantly (Fig 4A and 4B). Dextran uptake was marginally higher upon treatment with Chloroquine (Fig 4B).

The effect of Chloroquine in changing the endosomal pH of late endosomes was assessed. At different concentrations of Chloroquine tested, the endosomal pH measured using FITC/TMR ratio was only minimally increased (Fig 4C and 4D). We also observed that both FITC and TMR endosomal intensities increased with the concentration of Chloroquine. To confirm our results, we used another method to estimate endosomal pH. FITC has a pH-sensitive (488nm) and a pH-insensitive excitation (450nm) [56]. We used the 488/458 excitation ratio of FITC dextran as a readout of pH and found that this ratio also showed only a small albeit significant increase with Chloroquine when compared to control cells, unlike the increase brought about by NH_4_Cl (Fig 4E and 4F). This observation could explain the lack of an endocytic effect on RBD, dextran or transferrin uptake upon treatment with Chloroquine.

Despite this, we observed a marked reduction of viral transduction with Chloroquine treatment in AGS cells (Fig 4G and 4H) and HEK-293T cells (S10E–S10G Fig). Upon testing whether long term incubation of Chloroquine (as used in this assay) results in changes in endosomal pH or RBD uptake, we observed no alterations in the assessed phenotypes in AGS cells (S6A and S6B Fig). This suggests a distinct pH-independent mechanism of intervention by Chloroquine, functioning at the initial stages of infection.

### ACE2 biases RBD uptake via the clathrin-mediated endocytic pathway

**Fig 5:**
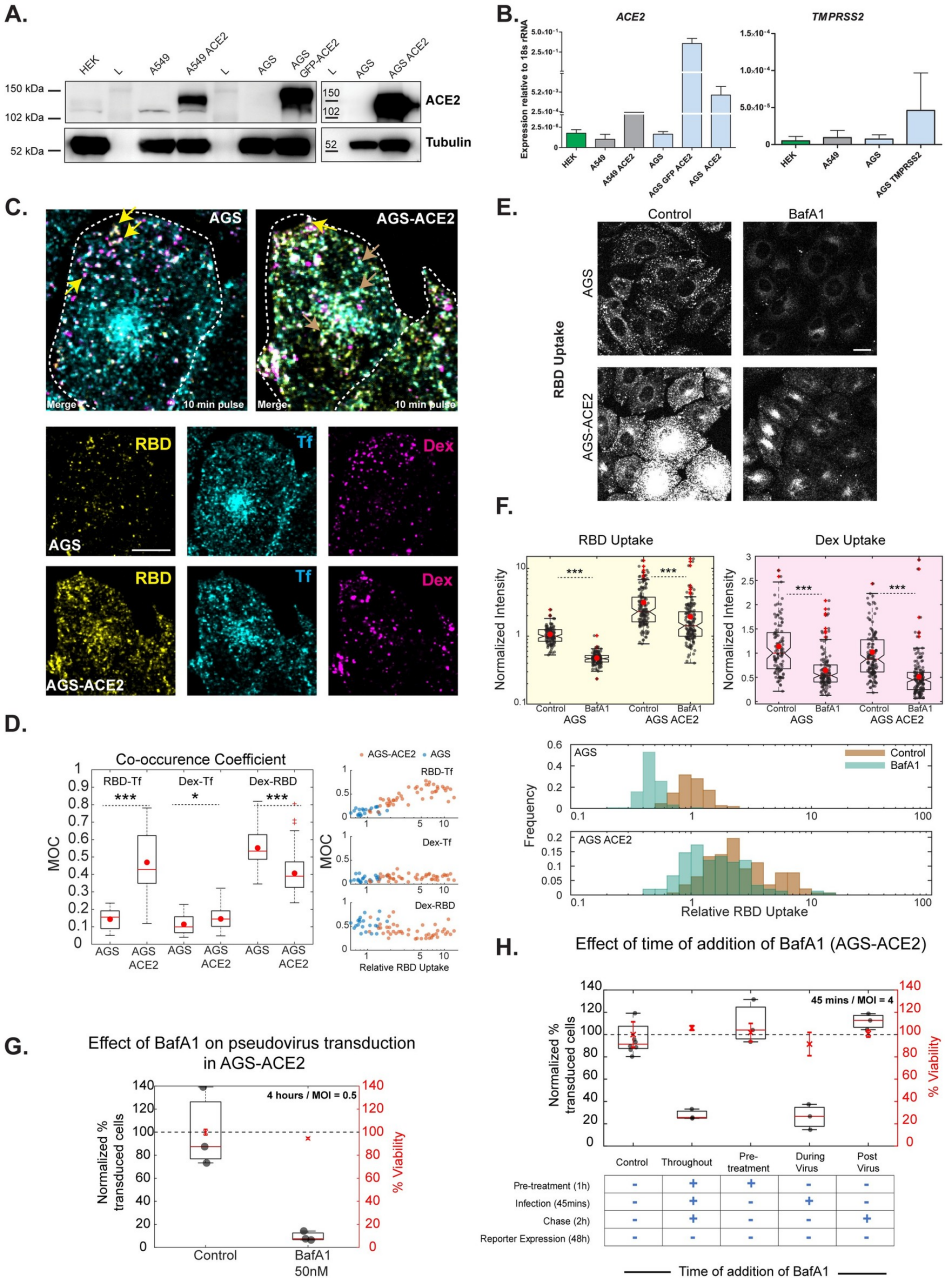
Effects of BafA1 on RBD uptake and Spike-pseudovirus transduction in AGS-ACE2 cells. A: HEKs, A549 and AGS cell lysates were evaluated for expression of ACE2 protein. Lysates from A549 and AGS cells stably overexpressing ACE2 were also included. AGS cells do not show any detectable levels of ACE2 protein expression while HEKs and A549 cells express the protein. Tubulin expression was used as housekeeping control. B: Transcript profiling in HEKs, A549 and AGS cells indicates ACE2 (i) and TMPRSS2 (ii) expression at low levels in all three cell lines. Expression is reported in each line relative to its 18s rRNA levels. C, D: AGS and AGS-ACE2 cells were pulsed with RBD, dextran and transferrin for 10 minutes and imaged at high resolution after fixation. Images are shown in C and quantification of Manders’ co-occurrence coefficient is shown in D. This compares the occurrence coefficient between RBD and transferrin (p-value < e-08), transferrin and dextran and RBD (p-value = 0.04) and dextran (p-value < e-03) between the two cell types. RBD is more co-localized to dextran endosomes in AGS cells, RBD is co-localized to both dextran and transferrin endosomes in AGS-ACE2 cells. The correlation coefficient between RBD-Tf increases with RBD-uptake. Number of cells = 21 (AGS), 45 (AGS-ACE2). Yellow arrow represents endosomes containing RBD and dextran. Gray arrow represents endosomes with RBD and transferrin. Dashed white line in C represents the approximate cell boundary. E, F: AGS and AGS-ACE2 cells were treated with Control (0.4% DMSO) or BafA1 400nM for 30 minutes and pulsed with RBD and dextran for 30 minutes with or without the inhibitor. Treatment with BafA1 reduces RBD (p-value < e-37 in AGS, < e-10 in AGS-ACE2) and dextran (p-value < e-13 in AGS, < e-20 in AGS-ACE2) uptake in both cell types. Images are shown in E and quantification in F. Numbers of cells > 100 for each treatment. G: Transduction efficiency is reduced with 50nM BafA1 (p-values < e-75). Control is 0.04%DMSO. Number of repeats = 3 for each treatment. H: AGS-ACE2 cells were treated with control or 50nM BafA1, pre/during/post incubation with virus as well as throughout all three stages. BafA1 significantly reduces transduction when used throughout (p-value < e-67) as well as during virus presentation (p-value < e-68). Only pre-treatment or post-treatment of BafA1 does not significantly change the percentage of transduction. Number of repeats = 8 for control and 3 for each condition. Data representation in F is as described in Fig 1, G and H is as described in Fig 3. Scale bar: 10μm (C) and 20μm (E).

To ascertain the mode of RBD and Spike-pseudovirus entry into AGS cells, we next measured the levels of known modulators–ACE2 and TMPRSS2. While low levels of ACE2 was detected in HEK-293T and A549 cells, we did not detect any ACE2 in AGS cells by western blot analysis (Fig 5A). However, low expression of ACE2 transcripts was observed by qPCR in all three cell types (Fig 5B). Thus, AGS can be considered as a cell line with undetectable levels of endogenous ACE2. TMPRSS2 levels measured using qPCR also showed low levels in all the cell lines tested. Spike-pseudovirus infection was competed out by excess RBD (S5E Fig), indicating an RBD-binding modality of entry in AGS cells. However, specific RBD-receptor interactions are unknown currently. Further studies interrogating specific RBD-receptor interactions in AGS cells will be required to determine the exact binding mechanisms.

To determine the effect of ACE2 on uptake of RBD in AGS cells, we generated a stable AGS cell line ectopically expressing ACE2 (AGS-ACE2); the expression of ACE2 was confirmed using qPCR and western blot analysis (Fig 5A). RBD uptake in AGS-ACE2 was about 3-fold higher than AGS cells (Fig 5E and 5F). On characterizing the RBD endocytic itinerary in AGS-ACE2 cells, we observed an increase in the co-occurrence of RBD with transferrin and slightly reduced co-occurrence of RBD with dextran compared to AGS cells (Fig 5C and 5D). This indicates that in addition to trafficking via the CG pathway, RBD is now trafficked via the CME in AGS-ACE2 cells. The AGS-ACE2 cell line has a heterogenous population of cells with a range of RBD uptake. We observed a positive trend between the co-occurrence coefficient of RBD with transferrin, and cellular RBD uptake (Fig 5D). This is consistent with the possibility that ACE2 biases RBD uptake towards CME.

### Effects of BafA1 on RBD uptake and Spike-pseudovirus infection in AGS-ACE2 cells

We next evaluated the effect of BafA1 on RBD and dextran endocytosis and observed a significant reduction in the uptake of both in AGS and AGS-ACE2 cells (Fig 5E and 5F). However, the absolute reduction of RBD uptake in AGS-ACE2 is not to the same extent as in AGS cells. This is likely because BafA1 only affects the CG fraction of uptake and has little effect on RBD entering via the CME. Additionally, BafA1 shows a similar reduction of normalized RBD uptake (normalized to the surface levels of ACE2) in AGS cells transiently overexpressing myc-ACE2 (Materials and Methods, S3C and S3D Fig).

AGS-ACE2 cell line provides an important tool to distinguish the effects brought about by acidification inhibitors on endocytosis and neutralization of acidic endosomes. This is because ACE2 biases RBD uptake towards CME and therefore, the effects of BafA1 in this cell line will more predominantly be due to its neutralization role in the endo-lysosomal network. In pseudovirus transduction assays, we observed a significant reduction in the percentage of infected AGS-ACE2 cells with BafA1 (Fig 5G). Thus, BafA1 robustly affects Spike-pseudovirus infection in experimental regimes where RBD enters cells via both endocytic pathways, taking it one step closer to being more universal in its action of preventing infection.

We next designed time-of-addition experiments to disentangle the possibilities of the involvement of BafA1 at different stages of the viral entry process in both AGS (Fig 3F) and AGS-ACE2 (Fig 5H) cells. These assays revealed that the BafA1 sensitive step is during the virus presentation (~45 minutes) in both cell lines. However, pre-treatment with BafA1 or post-treatment with BafA1, even as early as 45 minutes after pseudovirus presentation, does not inhibit viral entry in both cell lines. This confirms that the effect of BafA1 is restricted to the early time points of entry and the endosomal neutralization role of BafA1 is a necessary and sufficient step in controlling the infection. Additionally, in cell lines with low ACE2 (like AGS and HEK293T), BafA1 also restricts infection by restricting entry via the CG pathway. Dual mechanisms of inhibition offered by BafA1 can be considered as a potential strategy to explore similar pharmacologically active compounds to control SARS-CoV-2 infection.

### Identifying FDA-approved drugs functioning similar to BafA1 and NH_4_Cl

**Fig 6:**
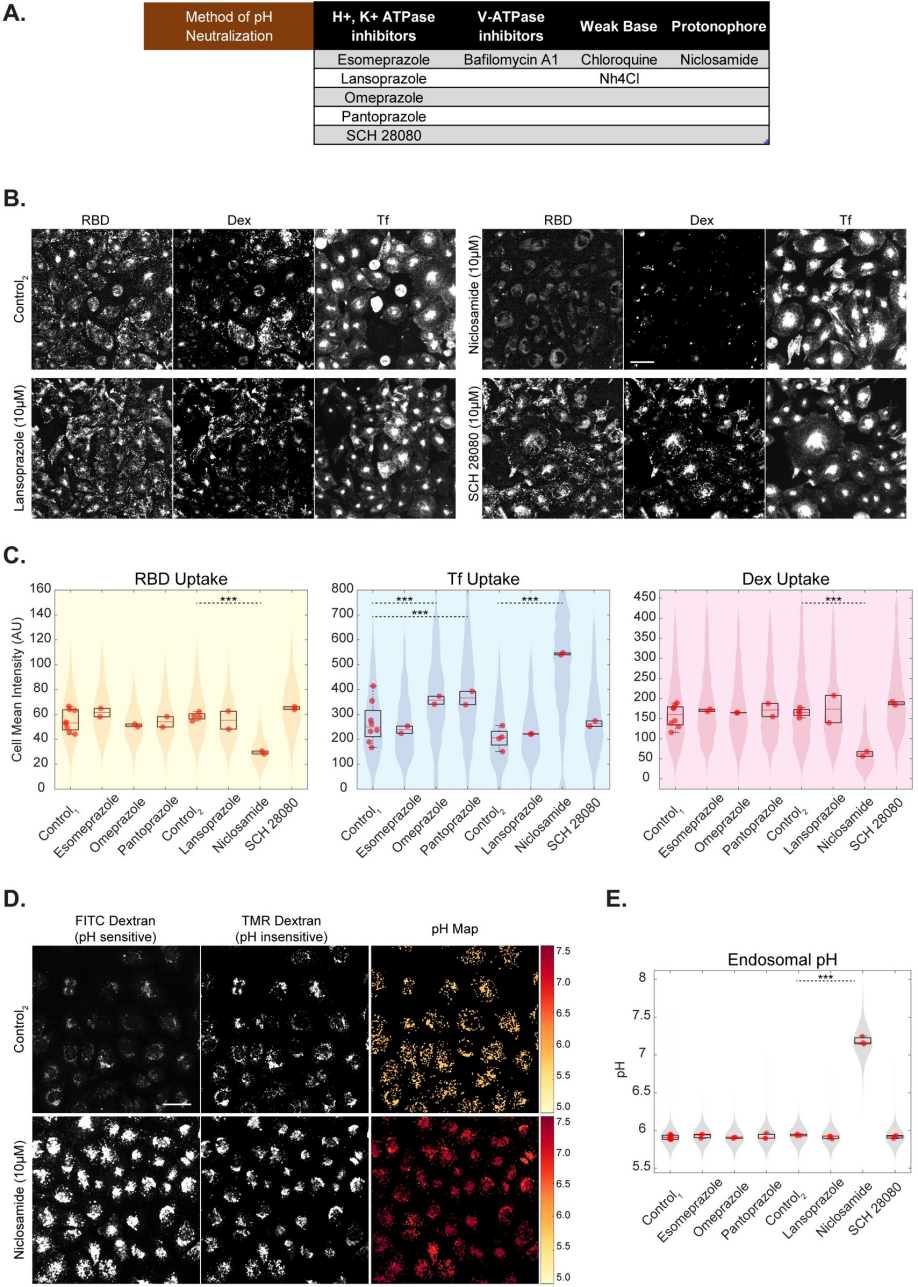
Identifying FDA-approved drugs functioning similar to BafA1 and NH_4_Cl. A: Table describing various methods of altering endosomal pH along with the chosen subset of drugs to screen for entry and acidification inhibition B, C: High-throughput assay in which AGS cells were treated with an array of drugs at 10μM concentration for 30 minutes and pulsed with RBD, dextran and transferrin for 30 minutes. Niclosamide shows reduction in RBD (p-value < e-195) and dextran (p-value < e-133) uptake and increase in transferrin uptake (p-value < e-155). Omeprazole (p-value < e-34) and Pantoprazole (p-value < e-38) show an increase in transferrin uptake and minimally affects RBD or dextran uptake. Images are shown in B (and in S7A), quantification is shown in C and p-value table for all the inhibitor treatments is indicated in S1 Table. Control_1_ is 0% DMSO and Control_2_ is 0.3% DMSO. Number of repeats ≥ 2 for each treatment and each repeat has >80 cells. D, E: High-throughput assay in which AGS cells were pulsed with FITC and TMR dextran for 2 hours, chased for 1 hour with an array of drugs or control and imaged live. Endosomal pH increases upon addition of Niclosamide (p-value < e-110). Images along with pH maps are depicted in D (and in S7B), quantification is shown in E (and in S7C) and p-value table for all the inhibitors is indicated in S1 Table. Control_1_ is 0% DMSO and Control_2_ is 0.2% DMSO. Number of repeats ≥ 3 for each treatment and each repeat has >80 cells. Data representation in C and E is as described in Fig 2. Scale bar: 40μm (B, D).

Armed with the knowledge on the modes of action of acidification inhibitors in reducing the uptake of RBD, increasing the pH of endosomes and abrogating the infection of Spike-pseuodovirus, we screened a small subset of FDA-approved drugs with the potential to alter the pH of endosomes (Fig 6A). We selected a panel of 6 drugs which includes those acting on Na+/K+ ATPase (Omeprazole, Esomeprazole, Pantoprazole, SCH-28080, Lansoprazole) and a protonophore that disrupts proton gradient (Niclosamide). We developed a quantitative high throughput screening pipeline for testing these drugs in both endocytic assay as well as pH estimation assay in AGS cells. The screen was carried out at a concentration of 10μM for all drugs.

Of the 6 drugs tested in the endocytosis assay, Niclosamide showed the strongest effect on the uptake of the 3 probes (RBD, dextran and transferrin) similar to what we observed for the acidification inhibitors. Niclosamide-treated cells showed reduced RBD and dextran uptake and increased transferrin uptake (Fig 6B and 6C). It is interesting to note that while the other proton pump inhibitors had minimal effects on RBD or dextran uptake at the concentration tested, Omeprazole and Pantoprazole showed a significant increase in transferrin uptake (Figs 6C and S7A). This suggests that these two drugs could specifically act on the transferrin containing endosomes and not in the compartments of relevance for RBD and dextran uptake, while Niclosamide inhibits the RBD and dextran uptake.

Of the 6 drugs tested in the late endosomal pH estimation assay, Niclosamide also showed the strongest neutralization effect on the pH of acidic endosomes (Fig 6E) by increasing the endosomal ratio of FITC/TMR (S7C Fig). The other drugs had minimal effects on the pH of late endosomes at the concentration tested (Figs 6E and S7B). The spatial pH maps of Niclosamide treated cells show an increase in pH in the majority of endosomes within the cell (Fig 6D). Niclosamide increased the FITC endosomal intensity and reduced the numbers of endosomes (S7C Fig) similar to the effect of BafA1 on these endosomal trafficking parameters.

Omeprazole and other proton pump inhibitors are prodrugs that are used for treating Gastro-esophageal reflux disease (GERD) [66]. They are activated by low pH, bind covalently to H+/K+ ATPase and inhibit the enzymatic function [67]. We tested the hypothesis if these drugs could also similarly block the proton pumps in the late endosomes and thus increase the endosomal pH [68,69]. Earlier studies have indicated that Omeprazole [70], Lansoprazole [71], and Pantoprazole [72], neutralize the endosomal pH only when used at very high concentrations (> 1mM) in EMT-6 and MCF-7 cells. However, the plasma concentration of these proton pump inhibitors varies between 1–23μM [66]. Thus, at least in the concentration range of relevance, we find no effect of these drugs on the acidification of endosomes and the uptake of RBD.

### Niclosamide functions similar to BafA1 and NH_4_Cl as an acidification and entry inhibitor

**Fig 7:**
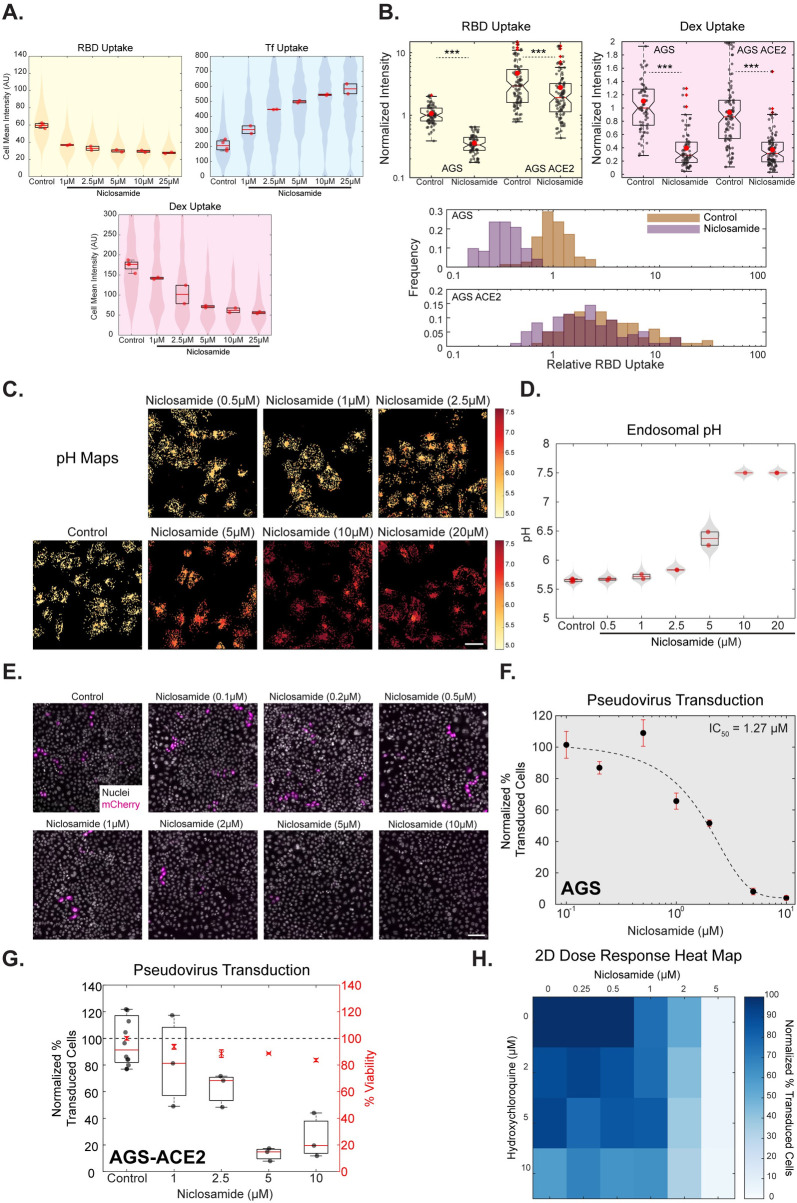
Niclosamide functions as an acidification and entry inhibitor. A: High-throughput endocytic assay in which AGS cells were treated with different concentrations of Niclosamide for 30 minutes followed by pulse of RBD, dextran and transferrin for 30 minutes. RBD and dextran uptake reduces, and transferrin uptake increases in a dose-dependent manner. Images are shown in S8A, quantification in 7A and S8B and p-value table for all the concentrations is indicated in S1 Table. Number of repeats = 4 for Control (0.6% DMSO) and 2 each for each concentration of Niclosamide. Each repeat has >80 cells. B: AGS and AGS-ACE2 cells were treated with Control (0.2% DMSO) or 10μM Niclosamide for 30 minutes and pulsed with RBD and dextran for 30 minutes with or without the inhibitor. Treatment with Niclosamide reduces RBD (p-value < e-24 in AGS, < e-3 in AGS-ACE2) and dextran (p-value < e-17 in AGS, < e-16 in AGS-ACE2) uptake in both cell types. Numbers of cells > 70 for each treatment. C, D: High-throughput pH estimation assay in which AGS cells were pulsed with FITC and TMR dextran for 2 hours, chased for 1 hour with different concentrations of Niclosamide and imaged live. A dose-dependent increase in endosomal pH is seen with increasing Niclosamide concentrations. Images along with pH maps are shown in 7C and quantification in 7D and S8C. p-value table is indicated in S1 Table. Number of repeats = 6 for Control, 2 each for each concentration of Niclosamide and 1 for 10μM Niclosamide. Each repeat has >80 cells. E, F, G: Spike-pseudovirus transduction assay in which AGS cells (7E, 7F, S9A) or AGS-ACE2 (7G) were preincubated for an hour with different concentrations of Niclosamide or DMSO and incubated along with virus (MOI = 0.5) for 8 hours (7E, 7F) or 4 hours (S9A, 7G) followed by the continued presence of 100nM Niclosamide or 0.005% DMSO in the media after removal of the virus until termination. Images of cells expressing the reporter mCherry protein in E and Normalized percentage transduction in F (AGS) and G (AGS-ACE2) show a dose-dependent reduction in transduction efficiency upon treatment with Niclosamide compared to pooled control from different DMSO treatments. Black dots with red error bars represent mean +/- SD. Dotted line represents the sigmoidal fit of the means across different Niclosamide concentrations. Refer to S9A(i) for % viability quantification, S9A(ii) for transduction efficiency after 4 hours of incubation with the virus in AGS cells and S1 Table for p-value table. H: 2-dimensional dose-response heat map in H depicts the combinatorial Spike pseudovirus transduction assay in AGS cells with indicated concentrations of Niclosamide (0–5μM) and Hydroxychloroquine (0–10μM). Normalized percentage transduction of cells is represented as a heat map. Refer to S9F for % viability quantification. Data representation in A and D is as described in Fig 2, in B is as described in Fig 1. Scale bar: 40μm (C) and 100μm (E).

Niclosamide is an anti-helminthic FDA-approved drug and has been in use since the 1960s (Ditzel, 1967). Many recent studies show that Niclosamide has broader clinical applications and has also been identified as an antiviral against SARS-CoV, human Rhinovirus, Influenza virus, Dengue virus [73,74]. As Niclosamide emerged as a potential drug candidate in both the RBD endocytosis as well as endosomal pH neutralization screens, we investigated the dose-dependent role of Niclosamide in reducing RBD uptake, neutralizing endosomal pH and inhibiting Spike-pseudovirus infection. We found that Niclosamide reduced both RBD and dextran uptake, as well as increase transferrin uptake in a dose-dependent manner (1–25μM) (Figs 7A and S8A). We observed Niclosamide’s effect on RBD endocytosis even at concentrations as low as 1μM. On analyzing the effect of Niclosamide on endosomal numbers and intensity, we found that Niclosamide increased the endosomal intensity of transferrin endosomes and reduced the number of RBD and dextran endosomes (S8B Fig). These effects are remarkably similar to the effects observed with acidification inhibitors–BafA1 and NH_4_Cl. We also confirmed the inhibitory effect of Niclosamide on RBD and dextran uptake in another cell line–HEK-293T (S10A–S10D Fig), and on normalized RBD uptake in AGS cells overexpressing ACE2 (S3C and S3D Fig). In AGS-ACE2 cells, similar to the effect of BafA1, Niclosamide also showed a reduction in RBD uptake (Fig 7B).

Further, we observed a dose-dependent effect of Niclosamide on neutralizing the pH of late endosomes and neutralization effects were seen even at 2.5μM (Fig 7C and 7D). The dose-response effect is seen on the ratio of endosomal FITC/TMR as well as other endosomal trafficking parameters—FITC and TMR endosomal intensities and numbers of endosomes (S8C Fig). The spatial pH maps of cells also show a gradual shift of endosomal pH from acidic to neutral pH with different doses of Niclosamide (Fig 7C), especially at 2.5μM wherein some endosomes within the cell are still acidic while some others are neutralized. Towards evaluating the effect of Niclosamide on the pH of early time point endosomes, AGS cells were labelled with FITC and TMR dextran for 20 minutes and chased for 10 minutes with or without Niclosamide for the entire duration of pulse and chase (S4E Fig). Unlike BafA1, Niclosamide reduced the net uptake of dextran. However, similar to BafA1, Niclosamide increased the endosomal FITC intensity and endosomal FITC/TMR ratio of early time point (30 minutes) endosomes (S4F Fig), indicating that Niclosamide neutralizes the pH of these endosomes as well.

We assessed the dose-dependent effect of Niclosamide on Spike-pseudovirus entry in AGS and AGS-ACE2 cells, using the experimental strategy designed to assess virus entry as described before. We observed a strong reduction of transduction efficiency as a function of increasing Niclosamide concentration at different viral incubation durations with negligible toxicity (Figs 7E and 7G and S9A), with an IC_50_ of ~1.27μM (Fig 7F and Materials and Methods). HEK-293T (S10E–S10G Fig) and A549-ACE2 (S10I Fig) cells also exhibited a similar inhibition of transduction upon treatment with Niclosamide. Together, all three assays provide proof of principle that a pH modulator functioning like BafA1, Niclosamide, can act as an entry inhibitor.

### Enhancing the inhibition of infectivity: A combination strategy with Niclosamide and Hydroxychloroquine

A combinatorial approach of drugs with varying mechanisms of inhibition works as an effective therapy to combat infection [75]. Given that Niclosamide exhibits a short half-life [76], has poor bio-availability (~10%) [77] and our observations indicate moderate IC_50_ for inhibition of Spike-pseudovirus transduction, we tested if the action of Niclosamide can be enhanced in the presence of another FDA-approved drug known to be effective against SARS-CoV-2 infection. Since published reports and commonly practised treatments against SARS-CoV-2 infection employ Hydroxychloroquine (HCQ), a less toxic variant of Chloroquine [78], we tested the effect of HCQ on altering late endosomal pH and Spike-pseudovirus transduction assay. Like Chloroquine, cells treated with 50μM HCQ also minimally altered the late endosomal pH (S9B and S9C Fig). However, we observed the pseudovirus transduction to be markedly reduced at HCQ concentrations of 50μM and 25μM (S9D and S9E Fig) and only modestly reduced at the concentrations of 10μM or lower in a dose-dependent manner (See the first box plot in S9Fi-iii Fig). To assess the synergistic effect of the two drugs, we chose a concentration range with the maximum concentrations of 10μM HCQ and 5μM Niclosamide. The 2-dimensional dose-response map shown in Fig 7H summarizes the effect of the two drugs on transduction. We observed an augmented reduction in infection when HCQ was used at a concentration of 10μM along with varying concentrations of Niclosamide compared to where HCQ was used at 0, 2 and 5μM (S9Fi-iii Fig). These results indicate an additive effect on inhibition of pseudovirus transduction when effective concentrations of HCQ is added along with effective concentrations of Niclosamide (Fig 7H). Thus, Niclosamide could potentially enhance the efficacy of other treatments currently being used to combat SARS-CoV-2 infection.

### Bafilomycin and Niclosamide inhibit SARS-CoV-2 infection in AGS-ACE2 and Vero cells

**Fig 8:**
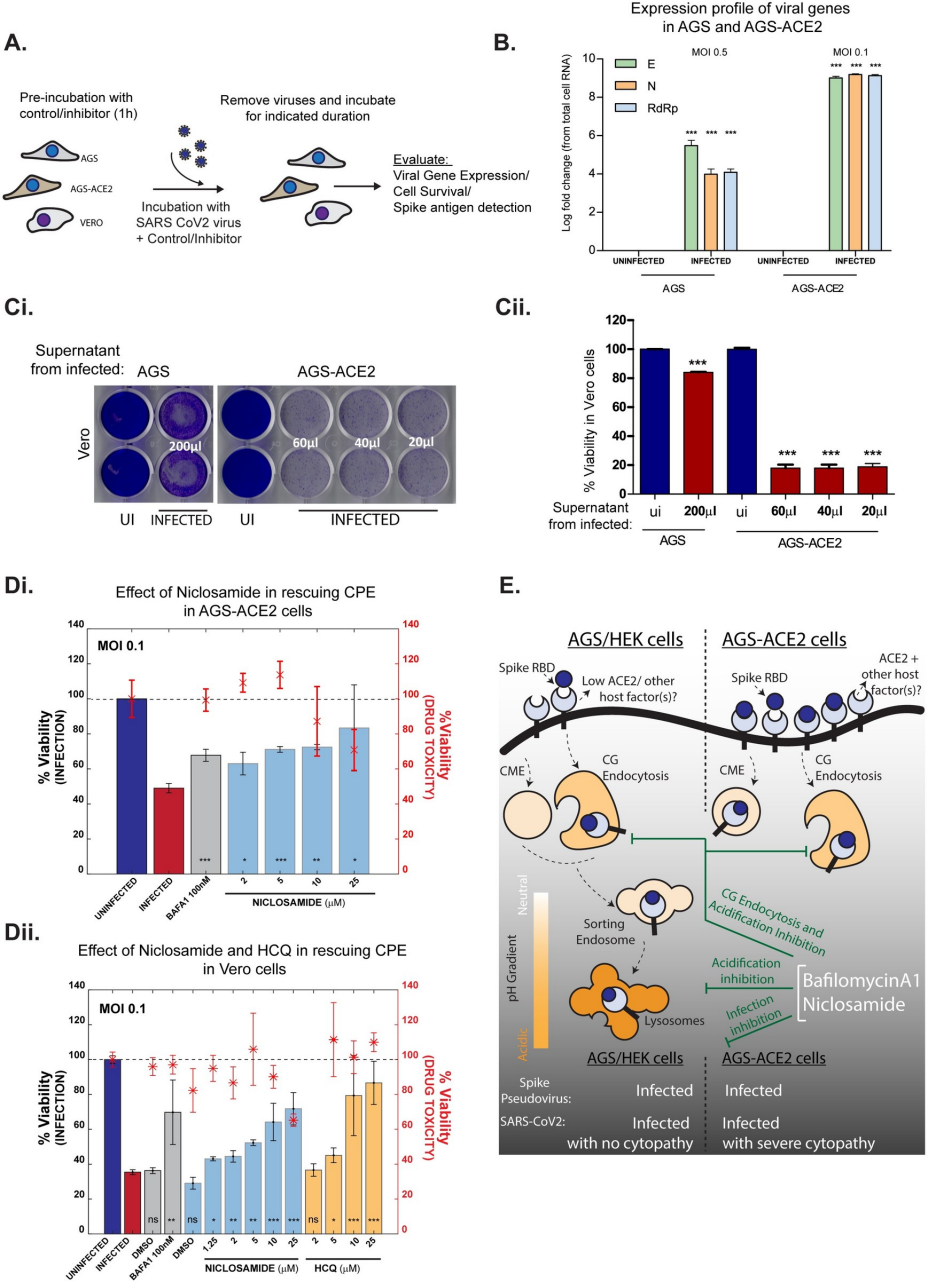
Validating the effect of endosomal acidification inhibitors on infection with SARS-CoV-2 virus. A: Schematic of viral infection assays in three cell types: AGS, AGS-ACE2 and Vero. Cells were pre-treated with vehicle controls or inhibitors for 1 hour and then presented with viruses at specified MOI for indicated duration. Viruses were then removed, and cells were further incubated in presence or absence of inhibitors. Infection efficiency was estimated using viral gene expression, cell viability or immunostaining for Spike antigen. B: Viral gene expression from cell lysates of infected AGS and AGS-ACE2 cells depicted as log fold change with respect to uninfected controls. Expression is normalized to 18s rRNA levels. Cells were infected with viruses at MOI 0.5 for AGS and MOI 0.1 for AGS-ACE2 for 30 minutes, washed and further incubated for 18 hours. Number of repeats = 3 for all conditions. C: Evaluating infectability of supernatants from infected AGS and AGS-ACE2. AGS and AGS-ACE2 were initially infected with viruses at MOI 0.5 and MOI 0.1 respectively for 8 hours, viruses were then removed, and cells were further incubated for 24 hours. Indicated volumes of culture supernatants were used for infecting Vero cells for a total of 96 hours (for supernatant from AGS) and 72 hours (for supernatant from AGS-ACE2). Following this, Vero cells were stained with crystal violet to assess cell viability post infection. Image of the stained plate is shown in (i) and quantification in (ii). Number of repeats = 2 (AGS/Vero) and 3 (AGS-ACE2 /Vero) for both uninfected and infected conditions. D: Assessing effect of endosomal acidification inhibitors on viral infection. AGS-ACE2 (i) and Vero cells (ii) were pre-treated with control or inhibitors for 1 hour followed by presentation of viruses at MOI 0.1 for 30 minutes (AGS-ACE2) or 8 hours (Vero) in the presence or absence of inhibitors. Viruses were removed and cells further incubated for 16 hours (AGS-ACE2) or 48 hours (Vero) and cell viability assessed by ATP quantification assay. Number of repeats = 3 for each condition. Bars represent mean +/- SD of % viability upon infection. Asterix with error bars in red represents the mean +/- SD of % viability indicating drug toxicity in the absence of viruses. E: In cells expressing low endogenous ACE2, Spike RBD endocytosis is aided by ACE2/other host cell factors via the CLIC/GEEC pathway. Overexpression of ACE2 results in trafficking of RBD through both CG and CME pathways. Acidification inhibitors BafA1 and FDA approved drug Niclosamide, neutralize the pH of endosomes as well as block entry via the CG pathway. Infection assays using both Spike pseudovirus and infectious SARS-CoV-2 virus confirm that BafA1 and Niclosamide prevent viral infection.

Our studies on RBD trafficking and Spike-pseudovirus infections were extended to the infectious SARS-CoV-2. Evaluation of viral gene transcripts following infection using qRT-PCR and estimation of cytopathic effects (CPE—host cell death due to viral infection) are commonly employed to assess viral infections [79,80] (Fig 8A). Appropriate experimental conditions (MOI and time of incubation) for each cell line were standardized by evaluating the time at which CPE appears. Infection was confirmed in AGS and AGS-ACE2 cells with qRT-PCR against viral genes; infected cell lysates of AGS and AGS-ACE2 cells show about 4 and 9 log fold increase, respectively at 18 hpi (hours post-infection) compared to corresponding uninfected controls (Fig 8B). Further, viral gene expression in infected AGS cell lysates and their supernatants indicated a persistent presence of these transcripts at 24, 48 and 72 hpi (S11C Fig). SARS-CoV-2 infection in AGS and AGS-ACE2 was additionally validated by collecting supernatants of infected cells and using them to re-infect Vero cells. Cell viability was reduced in Vero cells infected with AGS supernatant after 96 hpi. However, much lower volumes of supernatants from AGS-ACE2 cells affected Vero cell viability after 72 hpi, in line with the higher viral gene transcripts seen in these cells (Fig 8C).

CPE was observed as early as 8 hours of incubation with the virus in AGS-ACE2 cells, while Vero cells showed similar effects at a much later time point of 72 hours (S11A Fig). Interestingly, no such effects were observed in AGS cells even at a higher MOI of the virus at 8 hours (S11A Fig) and longer durations of 96 hours (S11A Fig). The extent of CPE was also quantified using cell viability assays (S11B Fig). Interestingly, similar to AGS cells, Calu-3 and Caco-2 cell lines which are permissive hosts to SARS-CoV-2 also do not exhibit CPE despite robust viral replication [79].

The effect of endosomal acidification inhibitors on SARS-CoV-2 infection was examined using CPE as a readout in AGS-ACE2 and Vero cells. Both BafA1 and Niclosamide showed a significant rescue in cell survival in AGS-ACE2 (Fig 8Di) and Vero cells (Fig 8Dii). Similar rescue effects were observed in AGS-ACE2 cells in a time-dependent manner (S11D Fig). Hydroxychloroquine was also effective in inhibiting viral infection in Vero cells with no associated toxicity (Fig 8Di). Further, immunostaining for Spike antigen in infected AGS-ACE2 cells suggested a decrease in viral load, as shown by a decrease in Spike intensity, in the presence of BafA1 and Niclosamide (S11E Fig). These observations emphasize the relevance of endosomal acidification inhibitors and validate their use as potential therapeutic candidates in restricting SARS-CoV-2 infection.

## Discussion

Understanding the molecular mechanisms of viral entry into relevant target cells is critical to design effective treatment and prevention strategies against infection. Employing various methodologies, we report for the first time that fluorescently labelled RBD of SARS-CoV-2 enters cells through a pH-dependent CG pathway in gastric origin AGS cells that have low or undetectable levels of ACE2 and utilizes CME in addition to CG upon ACE2 overexpression. High-resolution quantitative imaging approaches enabled us to detect the localization of RBD to acidic compartments. Endosomal acidification inhibitors that affect the uptake of CG cargo also inhibit RBD uptake. Complementing our observations with RBD, we show that infection by Spike-pseudovirus and clinical isolate of SARS-CoV-2 is also dependent on endosomal acidification. Further, by employing a targeted drug screen, we have identified Niclosamide as a potential inhibitor against SARS-CoV-2 entry and infection (Fig 8E).

The choice of viral entry into host cells is influenced by cell surface interacting partners and co-factors [12,28]. Although ACE2 has been identified as the receptor for SARS-CoV-2, other receptors are being uncovered. These include Neuropilin [13,14], CD147 [15], Heparan Sulphate proteoglycans [16] and HDL scavenger receptors [17]. Additionally, the highly glycosylated nature of Spike protein could also confer the ability to interact with yet unidentified receptors. It is well recognized that virus-receptor interactions, influenced by the levels of host receptor(s) and additional co-factors, dictate the endocytic route employed by the virus [81]. This is further exemplified by our observation that although RBD uptake is reduced upon blocking the CG pathway in AGS cells, residual RBD re-routes towards the CME and enables pseudovirus infection. Re-routing could be due to binding to different receptors that follow alternative internalization routes, and these remain uncharacterized in AGS cells. Additionally, ectopic expression of ACE2 in AGS cells results in trafficking RBD via CME in addition to the CG pathway. Whether the Spike-pseudovirus and SARS-CoV-2 follow routes of entry like RBD, can be addressed with tractable pseudoviruses or synthetic virus-like particles. SARS-CoV-2 infection has been reported in multiple cell types expressing low levels of ACE2 –pulmonary and olfactory epithelial cells [13]. CG pathway could be a preferred route of entry in such cell types while CME can be employed in cells with ACE2 expression (as reported here and [82]). Future studies can explore the contribution of host factor interactions and endocytic routes in understanding cell-type-specific pathological outcomes of viral infection.

Even though respiratory symptoms dominate the clinical presentation of COVID-19, a subset of patients also face gastrointestinal symptoms [83,84]. There is growing evidence for the involvement of the gastrointestinal tract in SARS-CoV-2 infection [25,85]. Transcriptomic and histochemical studies show that the gastrointestinal tract comprises of cell-types differentially expressing ACE2: oesophagus and proximal stomach have undetectable ACE2, distal stomach, duodenum, colon and rectum express high levels [86]. While our data demonstrate that both AGS (derived from stomach carcinoma) and AGS-ACE2 cells are permissive to SARS-CoV-2 infection, AGS-ACE2 cells show more severe cytopathic effects and extensive virus replication compared to AGS cells. These two cell lines could therefore offer suitable model systems to study SARS-CoV-2 infection in the gastrointestinal tract.

Known inhibitors of endosomal acidification, BafilomycinA1 and NH_4_Cl, play an important role in neutralizing acidic lysosomes and thus subverting viral membrane fusion and entry of several viruses [12,27–30]. Here, we report that these inhibitors also play a more upstream role by inhibiting the endocytosis of RBD itself. Both these treatments inhibited the uptake of CG cargo and RBD, reduced Spike-pseudovirus infection and drastically elevated endosomal pH. It is interesting to note that the inhibition of acidification in addition to dramatically reducing CG uptake did not cause re-trafficking of RBD through another endocytic pathway, as was observed for other CG inhibitors. This suggests that the acidification inhibitors could negatively influence the RBD-receptor interactions at the cell surface along with further ramifications of blocking the CG pathway. In both AGS and AGS-ACE2 cells, BafilomycinA1 was effective in inhibiting Spike-pseudovirus transduction when added at the time of virus infection but was ineffective when added post-infection (even as early as 45 minutes post-infection). BafilomycinA1 could, therefore, affect an early step in viral entry and not any downstream events such as viral protein translation or replication. The finding that BafilomycinA1 affects Spike-pseudovirus infection in AGS-ACE2 cells which employs both CG and CME routes of RBD entry, further validated that the inhibitor affects infection in the common acidic endo-lysosomal compartments where multiple endocytic pathways converge. We and others show that BafilomycinA1 also affects infection by VSV-G pseudotyped virus ([27] and S5Dii Fig) known to be endocytosed primarily via the CME [87,88]. Thus, targeting endosomal acidification can inhibit infection in a variety of cell types by affecting the very first step of endocytosis or the subsequent step of viral fusion in acidic endocytic compartments or both.

Dual mechanisms of inhibition offered by BafilomycinA1 encouraged us to screen a subset of FDA-approved compounds for similar inhibitory characteristics: proton-pump inhibitors (Omeprazole, Lansoprazole, Pantoprazole, Esomeprazole, SCH-28080), and protonophore (Niclosamide). Of all the 6 compounds tested only Niclosamide inhibited CG cargo and RBD uptake, elevated endosomal pH and concomitantly inhibited Spike-pseudovirus infection, all in a dose-dependent manner with an IC_50_ of 1.27 μM in AGS cells. Niclosamide also rescued cytopathic effects upon infection with a clinical isolate of SARS-CoV-2 in AGS-ACE2 and Vero cells. Among several mechanisms of action [76], Niclosamide disrupts proton gradient across mitochondrial [89] and endosomal [73] membranes. The elevated endosomal pH brought about by Niclosamide was shown to inhibit human rhinovirus infection [73]. Additionally, Niclosamide has been identified as an anti-viral agent against SARS-CoV [90], Dengue [74], MERS-CoV [91] and more recently proposed for SARS-CoV-2 (with IC_50_ of 0.28 μM in Vero cells) [92]. Niclosamide also affects calcium-activated chloride channels and scramblases and interferes with syncytia formation induced by SARS-CoV-2 spike protein [42]. Our finding that Niclosamide inhibits SARS-CoV-2 entry provides an additional mechanism for its anti-viral property. In contrast, the proton pump inhibitors used in our study failed to interfere with RBD uptake. This could be because they remained inactive [66] or the concentrations tested predominantly affect H+/K+ ATPases, while mM concentrations are required to inhibit V-ATPases [68]. Along these lines, studies show that proton pump inhibitors inhibit Ebola-pseudovirus [93], SARS-CoV and SARS-CoV-2 [94] infection only when used beyond achievable plasma concentrations [66].

Surprisingly, Chloroquine did not affect RBD uptake and only marginally raised the endosomal pH. However, it still affected Spike-pseudovirus infection in the initial steps of entry. The mechanism of action of Chloroquine appears to be distinct from BafilomycinA1 and Niclosamide. Chloroquine is likely to function in many pH-independent ways to inhibit SARS-CoV-2 infections. For example, by altering terminal glycosylation of ACE2 [95]; via its activity as a zinc ionophore affecting ACE2 activation [96,97]; by interacting with ER-resident Sigma receptors that initiate cell stress response [98]; by its ability to strongly bind a viral protease essential for Spike activation [99].

In conclusion, our study reports that the high-capacity CG pathway serves as a potential endocytic route for SARS-CoV-2 in cells with low/undetectable levels of ACE2, and via both CG and CME upon ectopic expression of ACE2. We further show that endosomal acidification is critical for SARS-CoV-2 entry and infection and can be a promising therapeutic target across multiple host cell types as observed by the results seen with Niclosamide, BafilomycinA1 and NH_4_Cl. This study also paves way for large-scale screens to test different chemical libraries including FDA-approved drugs as acidification inhibitors and scrutinize for more Niclosamide-like drugs that might have better bioavailability or can be used in combination with other antiviral drugs. Moreover, the methods described in our study can be effectively extended to primary cells and emerging organoid systems that represent the more natural hosts for infection.

## Materials and methods

*Cell lines*, *constructs*, *and antibodies*: See S1 Text for more details.

### Chemicals and reagents

Niclosamide and AN96 were chemically synthesized and proton pump inhibitors, Esomeprazole and Pantoprazole, were extracted from commercially available tablets as detailed in S1 Text. The other proton pump inhibitors, Lansoprazole and SCH-28080, were obtained from the LOPAC1280 library, and Omeprazole was procured from Sigma (O104).

### Endocytosis assays

AGS or HEK-293T cells were plated in 35mm coverslip bottom dishes and processed after 48 hours at 60–70% confluency. Cells were washed twice with HEPES buffer (wash and imaging buffer composition: 150mM NaCl, 20mM HEPES, 5mM KCl, 1mM CaCl_2_, 1mM MgCl_2_, 2mg/ml Glucose, pH 7.5) at 37°C. Endocytosis was monitored using fluorescently labelled RBD (Alexa/Atto 488, 10μg/ml), 10kDa TMR-dextran (1mg/ml) and/or Iron-loaded Transferrin (10μg/ml, Alexa 647) in serum-free medium for indicated time points at 37°C. Endocytosis was stopped using ice-cold wash buffer and cells were subsequently fixed with 2.5% paraformaldehyde (PFA) for 20 minutes at room temperature (RT). Cells were then washed and imaged. For inhibitor experiments, cells were pre-treated with various inhibitors (AN96 25μM, ML141 50μM, Amiloride 1mM, BafA1 200nM or 400nM, NH_4_Cl 30mM) and respective controls in serum-free medium for 30 minutes at 37°C and inhibitors were maintained during endocytic assays.

To measure normalized transferrin or normalized RBD uptake (Figs 1H–1I and S3C and S3D), cell surface-bound probes after the endocytic pulse with transferrin or RBD were stripped using two washes with ice-cold ascorbate buffer (160mM sodium ascorbate, 40mM ascorbic acid, 1mM MgCl_2_, 1mM CaCl_2_, pH 4.5), followed by three washes with ice-cold wash buffer at 4°C. Cells were then fixed with ice-cold 2.5% PFA for 5 mins at 4°C and 15 minutes at RT. Transferrin receptor (TfR) was labelled by incubating cells with fluorescently labelled anti-hTfR (OKT-9) for 2 hours at RT. To label surface ACE2, fixed cells were blocked with 10mg/ml bovine serum albumin (30 minutes) followed by incubation with anti-myc primary antibody (1 hour) and secondary antibody (45 minutes) in blocking buffer at RT. Cells were then washed and imaged.

### pH estimation assays

For estimating the pH of late endosomes, cells were pulsed with pH-sensitive 10kDa FITC-dextran (1mg/ml) and pH-insensitive 10kDa TMR-dextran (1mg/ml) for 2 hours in serum-free media, chased for 1 hour in the presence of inhibitors or control and imaged live. The above pulse and chase times were chosen to allow the accumulation of labelled dextran in acidic late endosomal and lysosomal compartments. To estimate the endosomal pH, the ratio of FITC to TMR fluorescence was computed and compared to a pH calibration curve (S4A and S4B Fig) which was generated by equalizing the endosomal pH to that of an external buffer. After the pulse with FITC and TMR-dextran and chase, cells were incubated with 5μg/ml nigericin containing buffers of different pH for 10 minutes and imaged to evaluate FITC/TMR ratios for each pH.

For estimating the pH of late endosomes using the 488/458 excitation ratio of FITC-dextran (Fig 4E and 4F), cells were pulsed with FITC-dextran at 1mg/ml for 2 hours, followed by chase in the presence or absence of inhibitors and imaged live.

For estimating the FITC/TMR ratio of early endosomes (S4E and S4F Fig), cells were incubated with pH-sensitive 10kDa FITC-dextran (1mg/ml) and pH-insensitive 10kDa TMR-dextran (1mg/ml) for 20 minutes, chased for 10 minutes and imaged live. Throughout the pulse and chase duration, the cells were incubated in serum-free media with control (0.2%DMSO) or BafA1 400nM or Niclosamide 10μM.

### Spike-pseudovirus transduction assays

AGS/HEK-293T cells were plated in optical bottom 96-well plates. 36 hours post-plating, when cell numbers were ~4000, transduction was carried out at indicated MOIs. For inhibitor treatment, cells were pre-incubated with indicated concentrations of NH_4_Cl/ BafA1/ CQ/ Niclosamide/ HCQ/ AN96/ ML141, for 1 hour. This was followed by addition of the Spike-pseudoviruses in presence or absence of the inhibitors. At the end of 2/4/8hours, media containing pseudoviruses and inhibitors was removed, and cells were washed once with drug-free media. This was followed by addition of media with or without inhibitor: NH_4_Cl, BafA1 and CQ were removed from the media; Niclosamide, AN96 and HCQ were maintained at a low concentration of 100nM, 1μM and 500nM respectively. This was done to assess the effects of the inhibitors at the initial stages of inhibition, minimize long-term toxicity to the cells as well as to avoid effects on the translational processes of the reporter gene post entry. After 60 hours, cells were fixed, nuclei were labelled with Hoescht and assessed for transduction efficiency based on mCherry reporter expression. In the case of HEK-293T cells (S10G Fig), MTT cell viability assay was performed to check toxicity (assay described in S1 Text).

### SARS-CoV-2 infection assays

SARS-CoV-2 (NR-52284 obtained from BEI-Resources) infections were conducted in AGS, AGS-ACE2 and Vero cells at the indicated MOIs and incubation durations as mentioned in the legends of Figs 8 and S11. Post infection, different assays were used to evaluate the extent of infection: Cell viability assays, Spike immunostaining and qPCR. Each assay is detailed in S1 Text. The effect of endosomal acidification inhibitors on SARS-CoV-2 infection were tested in AGS-ACE2 and Vero cells. Cells were pre-treated with inhibitors/vehicle controls at different concentrations for 1 hour, followed by infection in the presence of inhibitors/vehicle control. Virus and inhibitors were removed after indicated time of infection, cells were washed 3–4 times and incubated in virus free media with or without inhibitors. Post infection, Niclosamide treated cells were maintained in media with lower concentration of Niclosamide, while Bafilomycin treated cells were maintained in virus and drug free growth media until termination of the assay. To determine cytotoxicity of inhibitors, cells were treated with indicated concentrations of inhibitors in the absence of any virus presentation.

### Imaging and analysis

#### a. Endocytic and pH estimation assays

For 35mm dish-based endocytic experiments, fixed samples were imaged using confocal microscopy (Olympus FV3000, 20X/0.85NA objective) to image RBD, dextran and transferrin endosomes with Z sections of 1μm. Maximum intensity projected images were used for further analysis. Cell ROIs were drawn and features such as cell mean intensity in each channel was extracted.

For high-throughput endocytic and pH estimation experiments, automated imaging (Spinning disc, Phenix Perkin Elmer, 40XW/1.1NA objective) was used to image nucleus along with RBD, dextran and transferrin (for endocytosis) or FITC and TMR dextran (for pH) with Z sections of 1μm each. For both assays, cell profiler based pipeline was used to segment cells, nucleus and endosomes and extract features as described in S1 Text. For pH calibration, the mean of the endosomal ratio distributions at different extracellular pH was fit to a sigmoidal equation. For both assays, custom MATLAB routines were used to estimate the endosomal intensities, the number of endosomes and cell mean intensities. In addition, for pH assays, endosomal ratio (FITC/TMR) and endosomal pH (using the calibration curve) for each endosome was computed. As the endosomal intensity distribution within cells is a heavy right-tailed distribution, median endosomal intensity for each probe for each cell was estimated. The distributions of cell mean intensity/endosomal intensities/numbers of endosomes per cell per treatment (for endocytosis) and endosomal intensities/ratio/pH per cell per treatment (for pH) is represented in each quantification.

For 488/458 endosomal ratio estimation experiments, live imaging was done using confocal microscopy (Zeiss LSM 780, 40X/1.4NA objective). Excitation lasers 488nm, 458nm were used and emission was detected using a spectral detector (490nm-560nm). Images were processed as described above to estimate endosomal intensities and endosomal ratios per cell.

#### b. Colocalization analysis

Confocal microscopy (Olympus FV3000, 60X/1.42NA objective) with Z sections of 0.4μm each was employed to image cells across all channels. A MATLAB routine was written to extract colocalization indices. For each cell, endosomes in each channel were segmented based on threshold values. The segmentation in each channel was made finer using morphological operations (dilation followed by erosion). Segmented endosomes were considered for colocalization analysis. Manders’ coefficients and Pearson’s correlation coefficients were computed as described before [100].

#### c. Pseudovirus transduction assays

Automated imaging (Widefield, Phenix, 10X/0.3NA objective) of 96 well assay plates was used to image nucleus as well as mCherry positive cells. A cell profiler based pipeline was used to segment nucleus and extract features, as described in S1 Text. Approximately 50,000 nuclei (cells) were scored for each treatment. A MATLAB routine was written to estimate the % transduction. Mean intensities of the segmented nucleus in the nuclei channel and the mCherry channel for each nucleus across all fields were extracted. Each assay plate included “No-Virus” negative control. This control was used to estimate the background intensities of the mCherry channel within each segmented nucleus. The median of this distribution was considered the background. All nuclei with mCherry intensities of at least 1.8–2.2 times (empirically determined) the intensity of the background were considered positive. For each field, the fraction of positive nuclei to the total number of nuclei was determined. The mean of % transduction across all fields for each treatment was calculated. The % transduction was normalized to that of the control and is represented in all the quantifications. The total number of nuclei for each treatment is also represented to understand the effect of the toxicity of drugs.

### Statistical methods and hypothesis testing

Statistical tests between control and treatment were performed in MATLAB using Wilcoxon rank-sum test and the p-value of the hypothesis testing and the number of repeats is indicated in figure legends and S1 Table. In the entire manuscript, ***, **, * and ns indicate p-value of Wilcoxon rank-sum test < 0.001, <0.01, <0.05 and not significant, respectively. It is to be noted that p-value is affected by both the magnitude of differences as well as the sample size. For large sample size, as in case of our high throughput experiments, the impact of random error in measurement will be reduced and the larger magnitude of difference between the control and treatment will be associated with a much smaller p-value. Unpaired t-test was conducted using GraphPad Prism for data in Figs 8 and S11. Similar notation for indicating significance is represented as above.

## Supporting information

S1 Fig Generation of SARS-CoV-2 probe to study its endocytosis itinerary. A: Schematic describing the protocol for purification and fluorescent labelling of RBD. B: i) Image of a 10% SDS PAGE Gel showing the output from Ni-NTA purification of his-tagged RBD. Input is the culture supernatant containing secreted RBD (marked by a black box on the gel). FL is the flowthrough after binding the supernatant to the Ni-NTA column. RBD is eluted in fractions containing increasing concentrations of imidazole (50, 100, 150, 200, 250 mM). ii) Image of a 10% SDS PAGE Gel showing purified RBD after Gel filtration step of purification. L represents the ladder lane. C: AGS cells were transfected with myc-ACE2 and pulsed with RBD and transferrin for 30 minutes. Surface ACE2 was marked using anti-myc antibody. Myc-ACE2 transfected cells show increased RBD. D, E: AGS cells were transfected with myc-ACE2 and pulsed with RBD for 30 minutes. The cell surface-bound RBD was stripped using ascorbate buffer and cell surface ACE2 was labelled using anti-myc antibody. Images in D and scatter plot in E shows a positive correlation between the amount of RBD endocytosed and levels of surface ACE2. Number of cells >50. Scale bar: 40μm (C, D).
(TIF)

S2 Fig RBD uptake is sensitive to CG pathway inhibitors. A, B: AGS cells were pulsed with RBD, dextran and transferrin for 10 minutes and imaged at high resolution after fixation. Images in A and quantification in B shows that dextran and RBD are more correlated compared to dextran and transferrin (p-value < e-04) or transferrin and RBD (p-value < e-05) as measured using Pearson’s correlation coefficient (PCC). Number of cells = 10. C, D: AGS cells were treated with Control (0.6% DMSO) or AN96 25μM for 30 minutes, pulsed with RBD, dextran and transferrin for 30 minutes with Control or AN96 and imaged at high resolution upon fixation. Images are shown in C and quantification of Manders’ co-occurrence coefficient is shown in D. This depicts the fraction of RBD endosomal intensity with transferrin or dextran (i), the fraction of transferrin endosomal intensity with dextran or RBD (ii) and the fraction of dextran endosomal intensity with transferrin or RBD (iii). As seen in D(i), in control cells, the fraction of RBD endosomal intensity is more associated with dextran than transferrin (p-value < e-07). With AN96, internalized RBD and dextran is associated more with transferrin compared to control cells. Numbers of cells in each condition >10. p-value table is indicated in S1 Table. E, F: AGS cells were treated with Control or ML141 50μM for 30 minutes and pulsed with RBD and Dextran for 30 minutes with or without the inhibitor. RBD (p-value < e-9) and Dextran (p-value < e-20) uptake is significantly reduced upon treatment with ML141. Images are shown in E and quantification in F. Numbers of cells > 100 for each treatment. G, H: AGS cells were treated with Control (0.2% DMSO) or Amiloride 1mM for 30 minutes and pulsed with RBD, transferrin and dextran for 30 minutes with or without the inhibitor. RBD (p-value = 0.05), Dextran (p-value = 0.04) and transferrin (p-value = 0.013) uptake is not altered with Amiloride. Images are shown in G and quantification in H. Numbers of cells > 80 for each treatment. I: AGS cells were serum starved and treated with Control (0.2%DMSO), PMA alone (100nM), Amiloride alone (1mM) or in combination and pulsed with dextran for 30 minutes. Dextran uptake is enhanced with PMA; co-treatment with Amiloride abolishes this increase. Data representation is as described in Fig 1. Scale bar: 10 μm (A, C) and 40μm (E, G).
(TIF)

S3 Fig RBD uptake is sensitive to acidification inhibitors. A, B: AGS cells were treated with Control (0.3% DMSO, 0.6% DMSO, 0% DMSO) or inhibitors (BafA1 200nM, BafA1 400nM, NH_4_Cl 30mM) for 30 minutes and then pulsed with RBD, transferrin and dextran for 30 minutes with or without inhibitors. Images are shown in A and quantification in B with total cell mean intensity shown in (i), the number of endosomes shown in (ii) and intensity per endosome shown in (iii) for each probe in each condition. Control_1_ is 0.3% DMSO, Control_2_ is 0.6% DMSO and Control_3_ is 0% DMSO. Number of repeats ≥ 4 for each treatment and each repeat has >80 cells. C, D: AGS cells transfected with myc-ACE2 were treated with Control (0.2%DMSO) or BafA1 400nM or Niclosamide 10μM for 30 minutes and then pulsed with RBD for 30 minutes. The cell surface-bound RBD was stripped using ascorbate buffer and cell surface ACE2 was labelled using anti-myc antibody. Normalized RBD uptake is quantified as the ratio of the amount of internalized RBD to the amount of surface ACE2. Images depicted in C and quantification in D show that there is a reduction of RBD uptake upon treatment with BafA1 (p-value < e-08) or Niclosamide (p-value < e-07) in transfected as well as untransfected cells. Number of cells > 50 for each condition. Data representation in B and D are as described in Figs 2 and 1, respectively. Scale bar: 40μm (A, C).
(TIF)

S4 Fig Acidification inhibitors neutralize endosomal pH. A, B: pH calibration in AGS cells. AGS cells pulsed with pH-sensitive (FITC) and pH-insensitive (TMR) dextran were incubated in buffers of different pH with 5μg/ml of Nigericin and imaged live. A steady increase in the endosomal ratio of FITC/TMR with increasing pH is observed. The observed ratio vs clamped pH is fit to a sigmoidal curve (red curve) which is used as a calibration curve to estimate the pH of endosomes. Numbers of cells in each condition is >100 cells. The data in B is represented as mean +/- SD. C, D: For the experiment described in Fig 2F–2H, images including pH maps are shown in Fig 2F, Fig 2H, S4C and quantification in Fig 2G, S4D. FITC and TMR endosomal intensities, numbers of endosomes and FITC/TMR endosomal ratio are quantified in S4D. BafA1 200nM/400nM and NH_4_Cl increases FITC intensity and reduces numbers of endosomes. NH_4_Cl also affects trafficking as seen with an increase of TMR intensity. Endosomal ratio (as a proxy for endosomal pH) also shows an increase with all the acidification inhibitors. Control_1_ is 0.2% DMSO, Control_2_ is 0.4% DMSO and Control_3_ is 0% DMSO. Number of repeats ≥ 3 for each treatment and each repeat has >80 cells. p-value table is indicated in S1 Table. E, F: Estimation of FITC/TMR ratio of early endosomes. AGS cells were pulsed with FITC and TMR dextran for 20 minutes, chased for 10 minutes and imaged live. Throughout the pulse and chase duration, the cells were incubated with Control (0.2%DMSO) or BafA1 400nM or Niclosamide 10μM. Dextran uptake and TMR endosomal intensity are marginally reduced with Niclosamide while unaffected with BafA1. An increase in FITC endosomal intensity as well FITC/TMR endosomal ratio is observed with both inhibitors. Number of cells > 35 for each condition. p-value table is indicated in S1 Table. Data representation in D and F is as described in Figs 2 and 1, respectively. Scale bar: 40μm (A, C, E).
(TIF)

S5 Fig Characterization of SARS-CoV-2 spike pseudotyped virus. A: Schematic showing the strategy for generating SARS-CoV-2 Spike-pseudovirus. 2nd generation lentiviral helper plasmid psPAX was co-transfected with the reporter plasmid pHRmCherry and SARS-CoV-2 Spike protein-encoding plasmid pTwist Spike in HEK-293T cells to generate Spike pseudotyped virus particles. pHRmCherry reporter plasmid was used to score for infected cells by mCherry expression. B: Western blot showing bands of different molecular weights as detected by the anti-Strep-tag antibody which recognizes the C-term 2X Strep-tag on the Spike proteins incorporated into the pseudovirus particles. C: Comparison of infection by Spike-pseudotyped or bald pseudotyped (lacking spike) virus particles in AGS cells. Quantification shows that Spike-pseudotyped viruses infect AGS cells at various dilutions while bald-pseudoviruses do not infect at same dilutions. Number of repeats for each condition = 2 for each dilution. D: Transduction of AGS cells by Spike-pseudotyped viruses compared to an alternatively pseudotyped virus. (i) Quantification of infection at indicated MOI shows that VSV-G pseudotyped viruses are capable of transducing AGS cells at a higher efficiency. Number of repeats is 3 for Spike-pseudotyped viruses and 2 for VSV-G pseudotype. Data is plotted as mean +/- SD. (ii) Infection by VSV-G pseudotyped viruses is also susceptible to BafA1. Number of repeats = 2 for each condition. E: Specificity of Spike protein-ACE-2 dependent pseudovirus entry was tested in a competition assay in the presence of excess purified RBD of Spike. Quantification of percentage transduction shows a reduction in transduction efficiency with both monomeric and trimeric Spike RBD in AGS cells. Number of repeats = 2 for each condition. F: Characterization of transduction efficiency in AGS cells of Spike-pseudovirus at varying MOIs and varying incubation times. Quantification of percentage transduced mCherry positive cells depicts a steady increase in transduction as a function of MOI and incubation times. Number of repeats = 2 for each condition. The data is plotted as mean +/- SD. G: (i) Treatment of AGS cells across different concentrations of AN96 shows no significant reduction in transduction efficiency of Spike-pseudotyped viruses compared to the pooled controls from 0.6%, 0.24%, 0.12%, 0.048% and 0.024% DMSO treatments. Number of repeats = 15 for pooled controls and 3 for each concentration of AN96. (ii) Treatment of AGS cells with 5μM of ML141 shows no significant reduction in transduction efficiency compared to control (p-value = 0.55). Number of repeats = 3,2 for control (0% DMSO) and ML141 respectively. Data representation in C, D, E, G is as described in Fig 3.
(TIF)

S6 Fig Chloroquine does not affect RBD uptake and minimally affects endosomal acidification in AGS cells. A, B: Using AGS cells treated with Control or CQ for 12 hours, RBD, dextran and transferrin uptake experiment (A) and FITC/TMR endosomal ratio estimation experiment (B) was conducted. Quantification in G and H show that RBD uptake, dextran uptake and FITC/TMR endosomal ratio are unaffected by long term treatment with CQ. Number of cells > 80 for each condition. Data representation is as described in Fig 1.
(TIF)

S7 Fig Identifying FDA-approved drugs functioning similar to BafA1 and NH4Cl. A: For the experiment described in Figs 6B and 6C, images are shown in 6B and S7A and quantification in Fig 6C. p-value table is indicated in S1 Table. B, C: For the experiment described in Fig 6D and 6E, images including pH maps are shown in Figs 6D and S7B and quantification in Figs 6E and S7C. FITC and TMR endosomal intensities and numbers of endosomes are quantified in S7C. Niclosamide increases FITC intensity, reduces numbers of endosomes and has minimal effect on TMR intensity. p-value table is indicated in S1 Table.
(TIF)

S8 Fig Niclosamide functions like BafA1 in inhibiting RBD uptake and neutralizing the endosomal pH. A, B: For the endocytic assay experiment described in Fig 7A, images are shown in S8A Fig and quantification in Figs 7A and S8B, with total cell mean intensity shown in Fig 7A, number of endosomes shown in S8B(i) Fig and intensity per-endosome shown in S8B(ii) Fig. The number of RBD endosomes and dextran endosomes decrease, while transferrin endosomal intensity increases with increasing concentrations of Niclosamide. p-value table is indicated in S1 Table. C: For the pH estimation assay described in Fig 7C and7D, quantification of endosomal FITC intensities, TMR intensities and FITC/TMR endosomal ratio is shown in S8C. A dose-dependent increase in FITC endosomal intensity, as well as ratio, is seen with increasing Niclosamide concentrations. p-value table is indicated in S1 Table. Data representation in B, C is as described in Fig 2. Scale bar shown in A is 40μm.
(TIF)

S9 Fig Niclosamide and Hydroxychloroquine affect Spike-pseudovirus transduction in a dose-dependent manner. A: Quantification in A shows the normalized percentage transduction of Spike-pseudo virus across different concentrations of Niclosamide for incubation times of 8hours(i) and 4hours(ii) in AGS cells. Both the inhibitor and the virus were removed beyond the indicated times and cells were incubated with the continued presence of 100nM Niclosamide or 0.005% DMSO until termination. Images shown in Fig 7E and dose-response curve depicted in Fig 7F are related to the experiment in S9A(i). Number of repeats = 3 for each concentration of Niclosamide except 2 and 4 for 0.1μM and 0.2μM, respectively for the 8 hours set and 1 for 0.1μM Niclosamide in the 4 hours set. For the experiment in Fig 7G in AGS-ACE2 cells, number of repeats = 3 for each concentration of Niclosamide. p-value table is indicated in S1 Table. B, C: AGS cells pulsed with pH-sensitive (FITC) dextran for 2 hours and chased for 1 hour with Control or 50μM HCQ and imaged live. HCQ increases pH only slightly. pH maps are shown in B and quantification in C. Numbers of repeats: Control = 22, HCQ = 2. D, E: Images of AGS cells expressing the reporter mCherry protein upon transduction with Spike-pseudovirus in D and quantification in E show a dose-dependent reduction in transduction efficiency upon treatment with HCQ at the two concentrations tested compared to control (p-value < e-66 for HCQ 25μM, p-value < e-96 for HCQ 50μM). Number of repeats = 4 for control (0% DMSO) and 3 each for each concentration of HCQ. F: For the experiment described in Fig 7H, Quantification in S9F shows the normalized percentage transduction across indicated concentrations of Niclosamide in combination with indicated Hydroxychloroquine concentration of 2μM(i), 5μM(ii) and 10μM(iii). The percentage of cell viability for each condition is also indicated. Number of repeats = 2 for HCQ 5μM + Niclosamide 1μM combination and 3 each for all other combinations. p-value table is indicated in S1 Table. Data representation in A, E and F are as described in Fig 3 and C as described in Fig 2. Scale bar: 100μm (B).
(TIF)

S10 Fig BafA1 and Niclosamide affect RBD uptake in HEK-293T cells and reduce Spike pseudovirus infection in HEK-293T and A549-ACE2 cells. A-D: HEK-293T cells were treated with Control (0.4%DMSO), BafA1 400nM or Niclosamide 10μM for 30 minutes and pulsed with RBD and transferrin (A and B) or RBD and dextran (C and D) for 30 minutes with or without inhibitors. Images are shown in A and C, quantification is shown in B and D. RBD and dextran uptake is robustly reduced, while transferrin uptake increases upon treatment with BafA1 and Niclosamide. Number of cells ≥ 75 for each treatment. p-value table is indicated in S1 Table. E: Images show Spike-pseudovirus transduced mCherry positive HEK-293T cells in the presence of NH_4_Cl 20mM, CQ 10μM, BafA1 50nM and Niclosamide 5μM. F: Quantification of the normalized area of mCherry positive cells as a proxy for transduction, shows a reduction in transduction efficiency upon treatment with NH_4_Cl 20mM and CQ 10μM compared to 0% DMSO (Control_1_), 25nM and 50nM of BafA1 compared with 0.05%DMSO (Control_2_), 1μM and 5μM of Niclosamide compared to 0.1%DMSO (Control_3_). Number of repeats = 3 for each condition except 4 for 0%DMSO. The data is represented as mean +/- SD. G: Toxicity, as assessed by MTT based colorimetric assay, is represented as percentage viability of cells upon treatment with NH_4_Cl 20mM and CQ 10μM compared to 0% DMSO (Control_1_), 25nM and 50nM of BafA1 compared with 0.05%DMSO (Control2), 1μM and 5μM of Niclosamide compared to 0.1%DMSO (Control3). Number of repeats = 3 for each condition except 9 for 0%DMSO. The data is represented as mean +/- SD. H-I: Quantification of the mCherry positive cells in A549-ACE2 cells, as a proxy for transduction, shows a reduction in transduction efficiency upon treatment with 50nM of BafA1 in H and range of concentrations of Niclosamide in I (compared to respective DMSO controls). Data representation in B, D are as described in Fig 1 and H, I as in Fig 3. Scale bar: 40μm (A, C), 100μm (E).
(TIF)

S11 Fig Cytopathic effect of SARS-COV-2 in AGS-ACE2 and Vero cells is rescued by BafA1 and Niclosamide. A: AGS, AGS-ACE2 and Vero cells were infected with viruses at indicated MOI for 8, 8 and 72 hours respectively. AGS cells were also infected with indicated MOI for 96 hours. Post infection, bright field images show cytopathic morphology in both AGS-ACE2 and Vero but not in AGS. Images shown are representative of multiple independent experiments. B: Evaluation of cell viability at early time points post infection in AGS and AGS-ACE2 cells. Cells were infected with viruses at indicated MOI for 4 hours or 8 hours. Cell viability, assessed using an ATP quantification assay, indicates cytopathic effects in AGS-ACE2 cells. Percentage viability relative to uninfected control is depicted. Number of repeats = 3 for each condition. C: Viral gene expression in AGS cell lysates (i) and supernatants (ii) as a function of duration of infection. AGS and AGS-ACE2 cells were infected with viruses at indicated MOIs for the specified time periods post infection. Expression is depicted as log fold change compared to uninfected cells in (i), raw Ct values of viral gene transcripts from culture supernatants in (ii). NTC: no transcript control; PTC: positive transcript control. Number of repeats = 3 (uninfected and infected AGS), 1 (uninfected AGS-ACE2) and 2 (infected AGS-ACE2). D: Effect of endosomal acidification inhibitors on SARS-CoV2 infection in AGS-ACE2 cells. Cells were pre-treated with control/inhibitor for 1 hour at indicated concentrations and infected with virus for 6 hours in the presence/absence of inhibitors. Viruses were then removed, and cells were further incubated for 0, 6 or 12 hours. Cells treated with Niclosamide were maintained at 1μM post infection. Upon termination, cell viability was assessed by ATP quantification assay. Number of repeats = 3 for each condition. E: Detection of SARS-CoV-2 Spike antigen in infected AGS-ACE2 cells. Cells were pre-treated with control/inhibitor for 1 hour followed by infection with viruses at indicated MOI for 30 minutes in the presence/absence of inhibitors. Viruses were removed and cells further maintained for 16 hours. Cells treated with Niclosamide were maintained at 1μM post infection. Cells were fixed and stained for Spike antigen. Representative images are shown in (i) and quantification as violin plot in (ii). Number of repeats = 2 for each condition. Intensity of Spike staining is compared to uninfected and DMSO-treated cells.
(TIF)

S1 Table p-value table for various experiments (in bold) is detailed in S1 Table. Statistical tests between control and treatment were performed using Wilcoxon rank-sum test.
(TIF)

S1 Text Methods of different assays such as preparation of RBD and Spike Pseudovirus, titre detrmination of Spike pseudovirus and SARS-CoV-2 virus, Cell viability assays, qPCR and western blot techniques, image segmentation methods, synthesis of Niclosamide, extraction of Esomoprazole and Pantoprazole are detailed in S1 Text. (PDF)
