# Supplementary figures and images for: Strategies to target SARS-CoV-2 entry and infection using dual mechanisms of inhibition by acidification inhibitors
Source: PLoS Pathog. 2021 Jul 12;17(7):e1009706. doi: 10.1371/journal.ppat.1009706 (PMC8297935; doi:10.1371/journal.ppat.1009706)

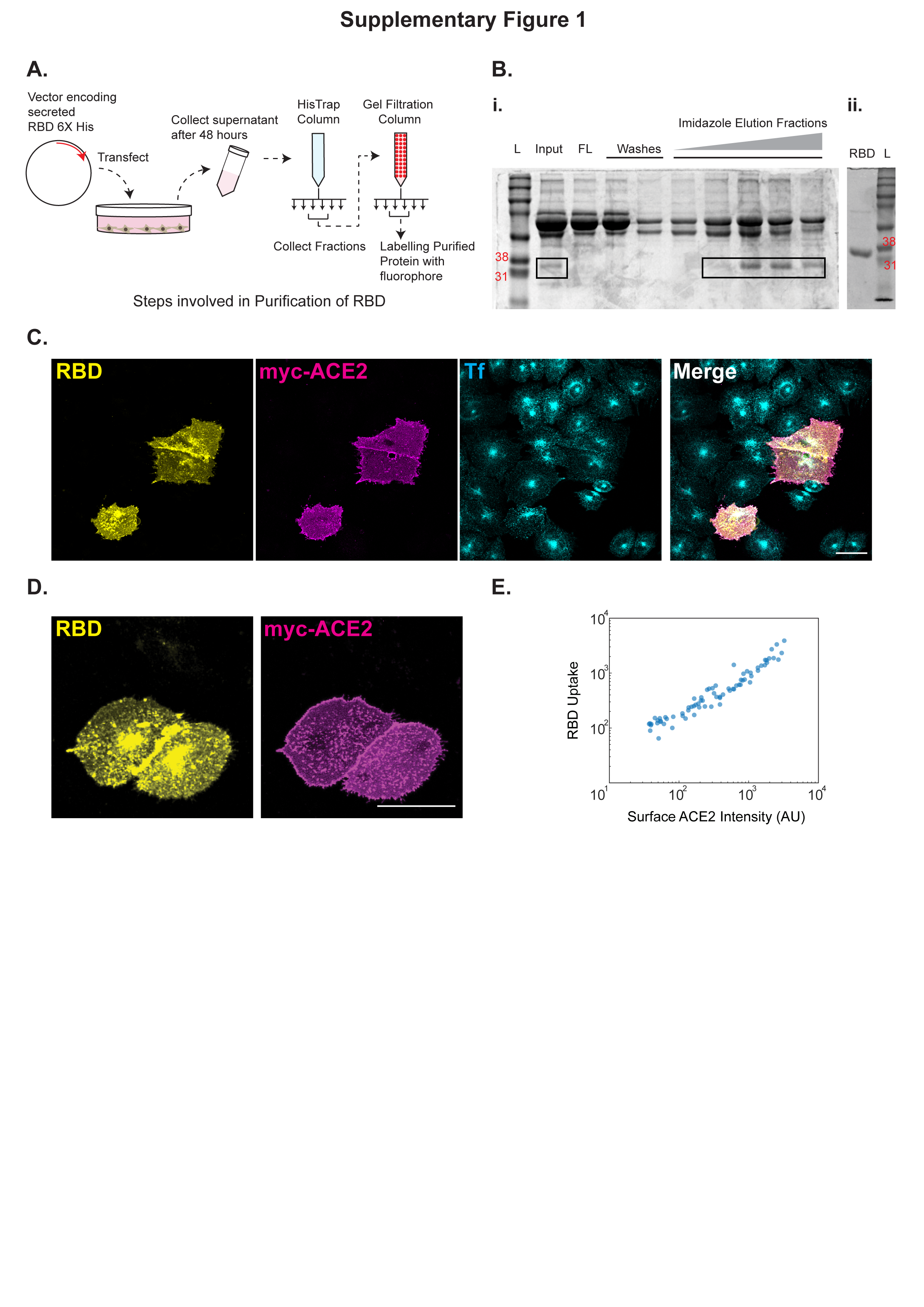

Supplement: S1 Fig — A: Schematic describing the protocol for purification and fluorescent labelling of RBD. B: i) Image of a 10% SDS PAGE Gel showing the output from Ni-NTA purification of his-tagged RBD. Input is the culture supernatant containing secreted RBD (marked by a black box on the gel). FL is the flowthrough after binding the supernatant to the Ni-NTA column. RBD is eluted in fractions containing increasing concentrations of imidazole (50, 100, 150, 200, 250 mM). ii) Image of a 10% SDS PAGE Gel showing purified RBD after Gel filtration step of purification. L represents the ladder lane. C: AGS cells were transfected with myc-ACE2 and pulsed with RBD and transferrin for 30 minutes. Surface ACE2 was marked using anti-myc antibody. Myc-ACE2 transfected cells show increased RBD. D, E: AGS cells were transfected with myc-ACE2 and pulsed with RBD for 30 minutes. The cell surface-bound RBD was stripped using ascorbate buffer and cell surface ACE2 was labelled using anti-myc antibody. Images in D and scatter plot in E shows a positive correlation between the amount of RBD endocytosed and levels of surface ACE2. Number of cells >50. Scale bar: 40μm (C, D). (TIF) [file ppat.1009706.s001.tif]

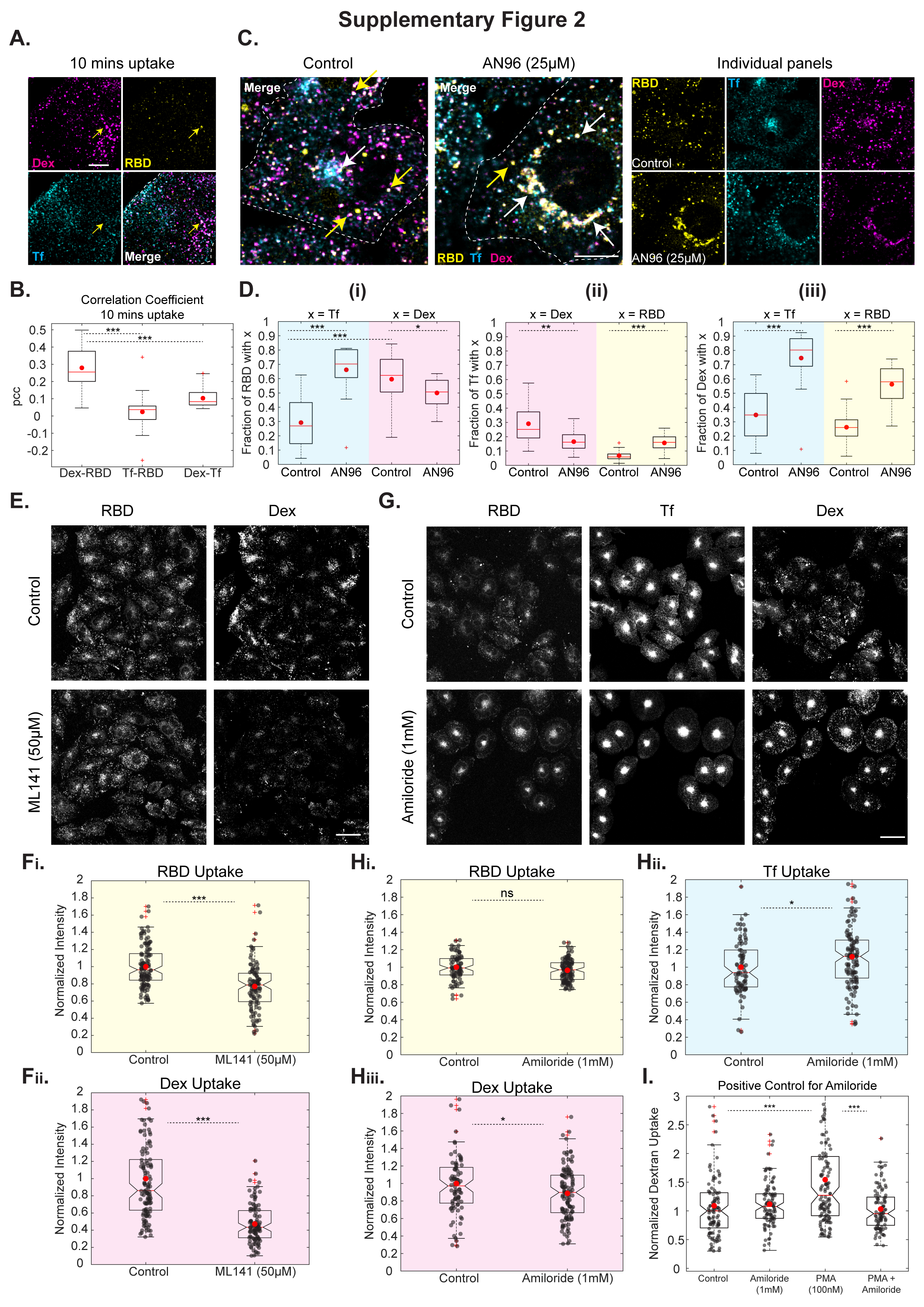

Supplement: S2 Fig — A, B: AGS cells were pulsed with RBD, dextran and transferrin for 10 minutes and imaged at high resolution after fixation. Images in A and quantification in B shows that dextran and RBD are more correlated compared to dextran and transferrin (p-value < e-04) or transferrin and RBD (p-value < e-05) as measured using Pearson’s correlation coefficient (PCC). Number of cells = 10. C, D: AGS cells were treated with Control (0.6% DMSO) or AN96 25μM for 30 minutes, pulsed with RBD, dextran and transferrin for 30 minutes with Control or AN96 and imaged at high resolution upon fixation. Images are shown in C and quantification of Manders’ co-occurrence coefficient is shown in D. This depicts the fraction of RBD endosomal intensity with transferrin or dextran (i), the fraction of transferrin endosomal intensity with dextran or RBD (ii) and the fraction of dextran endosomal intensity with transferrin or RBD (iii). As seen in D(i), in control cells, the fraction of RBD endosomal intensity is more associated with dextran than transferrin (p-value < e-07). With AN96, internalized RBD and dextran is associated more with transferrin compared to control cells. Numbers of cells in each condition >10. p-value table is indicated in S1 Table. E, F: AGS cells were treated with Control or ML141 50μM for 30 minutes and pulsed with RBD and Dextran for 30 minutes with or without the inhibitor. RBD (p-value < e-9) and Dextran (p-value < e-20) uptake is significantly reduced upon treatment with ML141. Images are shown in E and quantification in F. Numbers of cells > 100 for each treatment. G, H: AGS cells were treated with Control (0.2% DMSO) or Amiloride 1mM for 30 minutes and pulsed with RBD, transferrin and dextran for 30 minutes with or without the inhibitor. RBD (p-value = 0.05), Dextran (p-value = 0.04) and transferrin (p-value = 0.013) uptake is not altered with Amiloride. Images are shown in G and quantification in H. Numbers of cells > 80 for each treatment. I: AGS cells were serum st [file ppat.1009706.s002.tif]

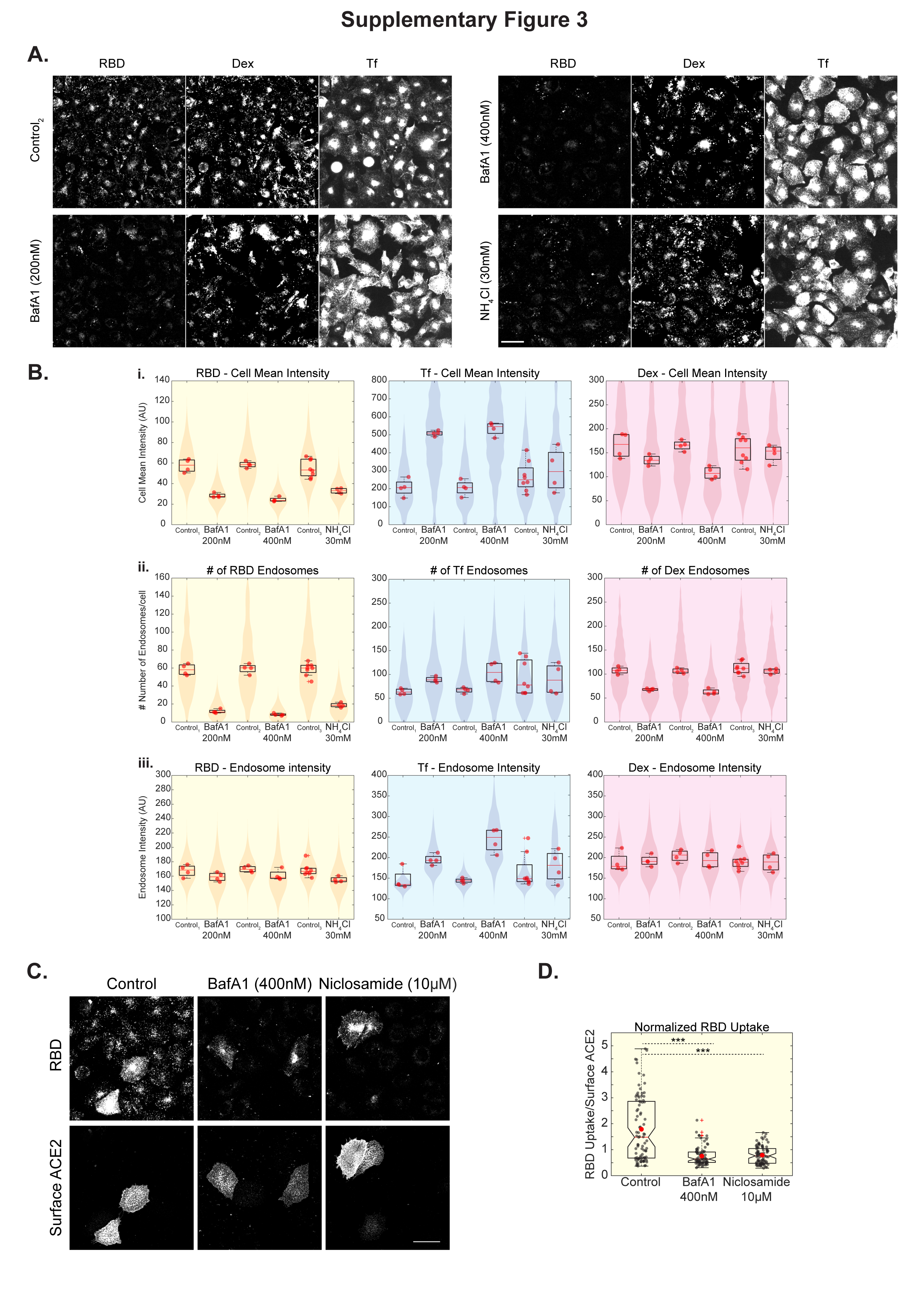

Supplement: S3 Fig — A, B: AGS cells were treated with Control (0.3% DMSO, 0.6% DMSO, 0% DMSO) or inhibitors (BafA1 200nM, BafA1 400nM, NH4Cl 30mM) for 30 minutes and then pulsed with RBD, transferrin and dextran for 30 minutes with or without inhibitors. Images are shown in A and quantification in B with total cell mean intensity shown in (i), the number of endosomes shown in (ii) and intensity per endosome shown in (iii) for each probe in each condition. Control1 is 0.3% DMSO, Control2 is 0.6% DMSO and Control3 is 0% DMSO. Number of repeats ≥ 4 for each treatment and each repeat has >80 cells. C, D: AGS cells transfected with myc-ACE2 were treated with Control (0.2%DMSO) or BafA1 400nM or Niclosamide 10μM for 30 minutes and then pulsed with RBD for 30 minutes. The cell surface-bound RBD was stripped using ascorbate buffer and cell surface ACE2 was labelled using anti-myc antibody. Normalized RBD uptake is quantified as the ratio of the amount of internalized RBD to the amount of surface ACE2. Images depicted in C and quantification in D show that there is a reduction of RBD uptake upon treatment with BafA1 (p-value < e-08) or Niclosamide (p-value < e-07) in transfected as well as untransfected cells. Number of cells > 50 for each condition. Data representation in B and D are as described in Figs 2 and 1, respectively. Scale bar: 40μm (A, C). (TIF) [file ppat.1009706.s003.tif]

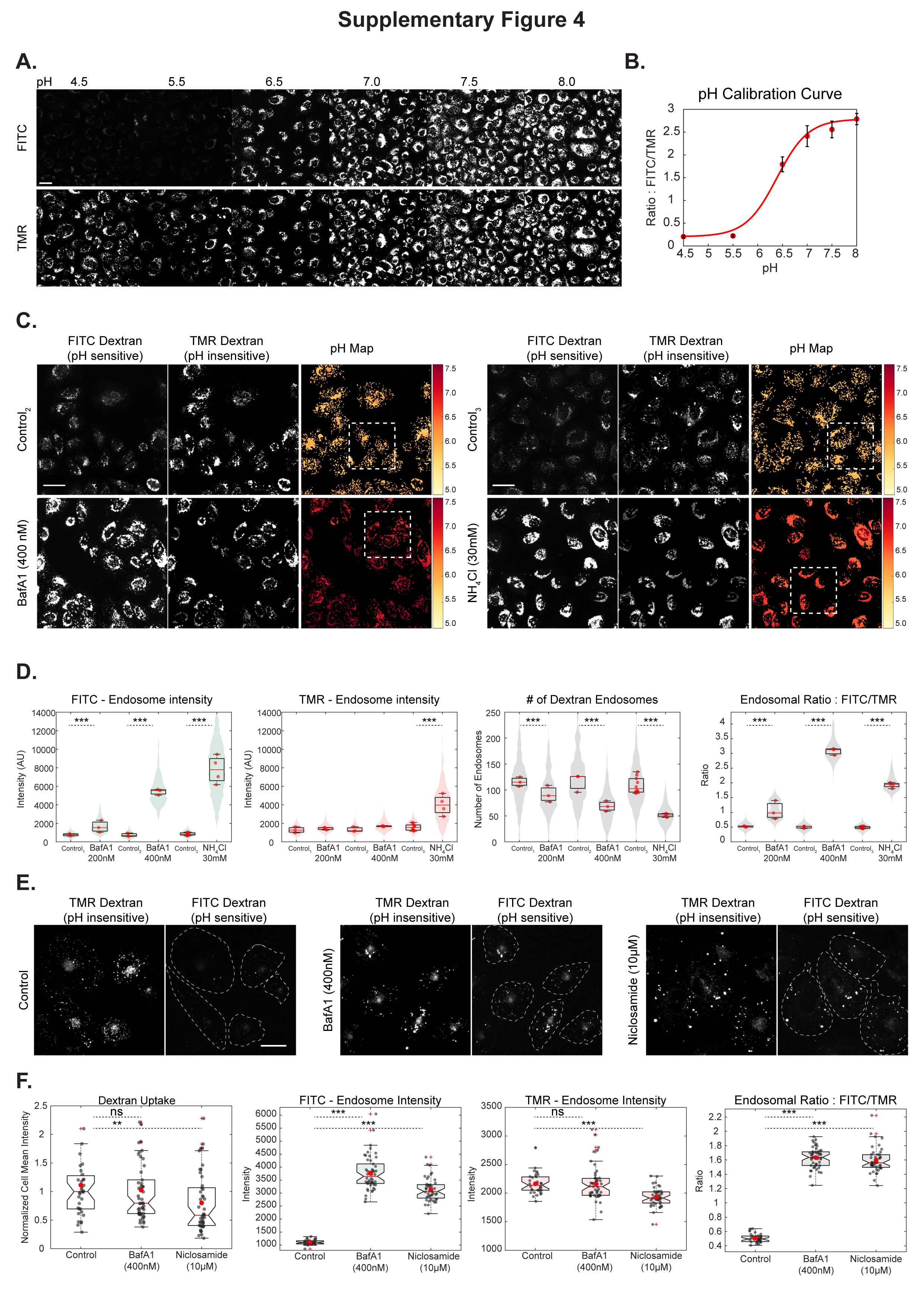

Supplement: S4 Fig — A, B: pH calibration in AGS cells. AGS cells pulsed with pH-sensitive (FITC) and pH-insensitive (TMR) dextran were incubated in buffers of different pH with 5μg/ml of Nigericin and imaged live. A steady increase in the endosomal ratio of FITC/TMR with increasing pH is observed. The observed ratio vs clamped pH is fit to a sigmoidal curve (red curve) which is used as a calibration curve to estimate the pH of endosomes. Numbers of cells in each condition is >100 cells. The data in B is represented as mean +/- SD. C, D: For the experiment described in Fig 2F–2H, images including pH maps are shown in Fig 2F, Fig 2H, S4C and quantification in Fig 2G, S4D. FITC and TMR endosomal intensities, numbers of endosomes and FITC/TMR endosomal ratio are quantified in S4D. BafA1 200nM/400nM and NH4Cl increases FITC intensity and reduces numbers of endosomes. NH4Cl also affects trafficking as seen with an increase of TMR intensity. Endosomal ratio (as a proxy for endosomal pH) also shows an increase with all the acidification inhibitors. Control1 is 0.2% DMSO, Control2 is 0.4% DMSO and Control3 is 0% DMSO. Number of repeats ≥ 3 for each treatment and each repeat has >80 cells. p-value table is indicated in S1 Table. E, F: Estimation of FITC/TMR ratio of early endosomes. AGS cells were pulsed with FITC and TMR dextran for 20 minutes, chased for 10 minutes and imaged live. Throughout the pulse and chase duration, the cells were incubated with Control (0.2%DMSO) or BafA1 400nM or Niclosamide 10μM. Dextran uptake and TMR endosomal intensity are marginally reduced with Niclosamide while unaffected with BafA1. An increase in FITC endosomal intensity as well FITC/TMR endosomal ratio is observed with both inhibitors. Number of cells > 35 for each condition. p-value table is indicated in S1 Table. Data representation in D and F is as described in Figs 2 and 1, respectively. Scale bar: 40μm (A, C, E). (TIF) [file ppat.1009706.s004.tif]

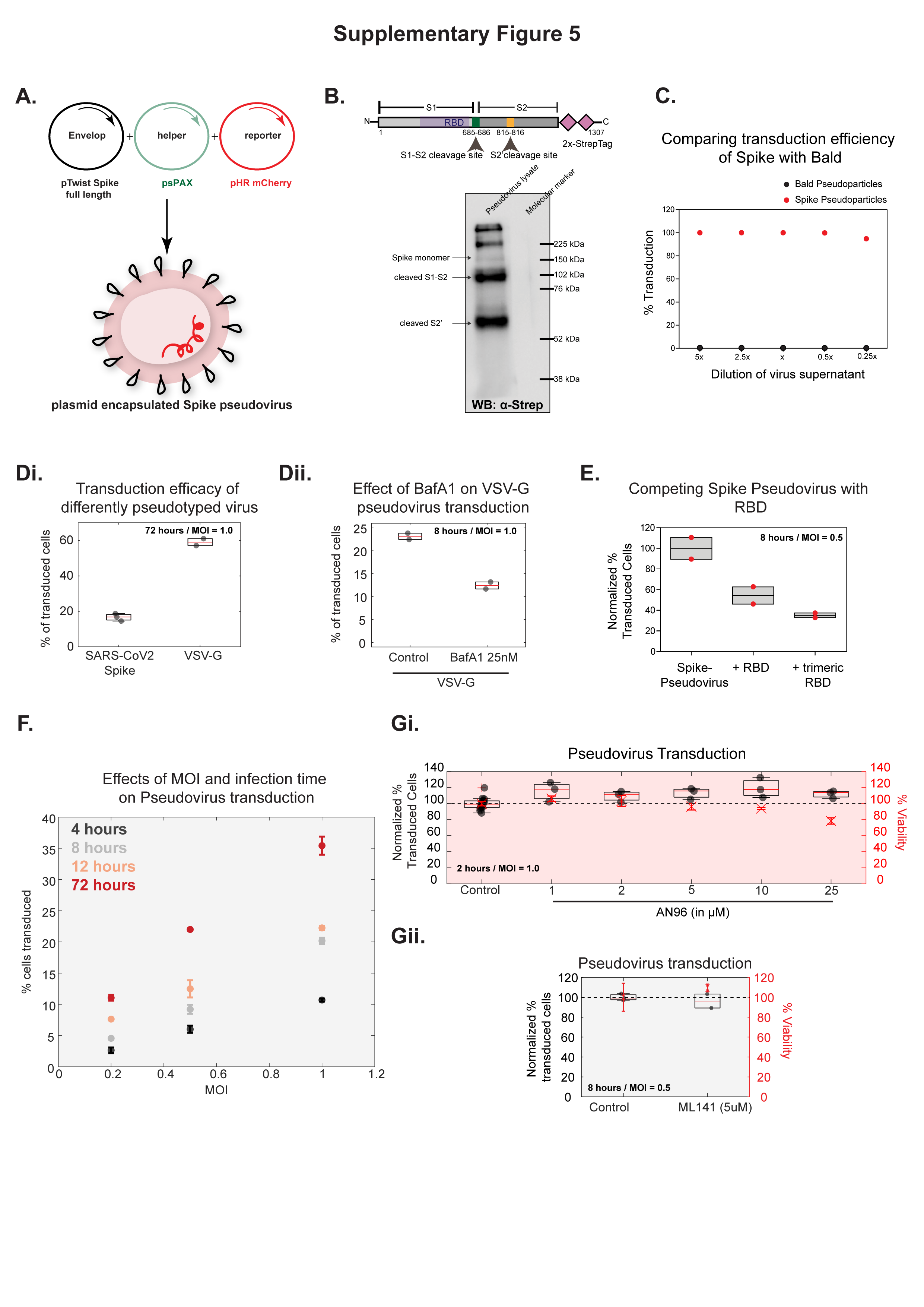

Supplement: S5 Fig — A: Schematic showing the strategy for generating SARS-CoV-2 Spike-pseudovirus. 2nd generation lentiviral helper plasmid psPAX was co-transfected with the reporter plasmid pHRmCherry and SARS-CoV-2 Spike protein-encoding plasmid pTwist Spike in HEK-293T cells to generate Spike pseudotyped virus particles. pHRmCherry reporter plasmid was used to score for infected cells by mCherry expression. B: Western blot showing bands of different molecular weights as detected by the anti-Strep-tag antibody which recognizes the C-term 2X Strep-tag on the Spike proteins incorporated into the pseudovirus particles. C: Comparison of infection by Spike-pseudotyped or bald pseudotyped (lacking spike) virus particles in AGS cells. Quantification shows that Spike-pseudotyped viruses infect AGS cells at various dilutions while bald-pseudoviruses do not infect at same dilutions. Number of repeats for each condition = 2 for each dilution. D: Transduction of AGS cells by Spike-pseudotyped viruses compared to an alternatively pseudotyped virus. (i) Quantification of infection at indicated MOI shows that VSV-G pseudotyped viruses are capable of transducing AGS cells at a higher efficiency. Number of repeats is 3 for Spike-pseudotyped viruses and 2 for VSV-G pseudotype. Data is plotted as mean +/- SD. (ii) Infection by VSV-G pseudotyped viruses is also susceptible to BafA1. Number of repeats = 2 for each condition. E: Specificity of Spike protein-ACE-2 dependent pseudovirus entry was tested in a competition assay in the presence of excess purified RBD of Spike. Quantification of percentage transduction shows a reduction in transduction efficiency with both monomeric and trimeric Spike RBD in AGS cells. Number of repeats = 2 for each condition. F: Characterization of transduction efficiency in AGS cells of Spike-pseudovirus at varying MOIs and varying incubation times. Quantification of percentage transduced mCherry positive cells depicts a steady increase in transduction as a function of MOI an [file ppat.1009706.s005.tif]

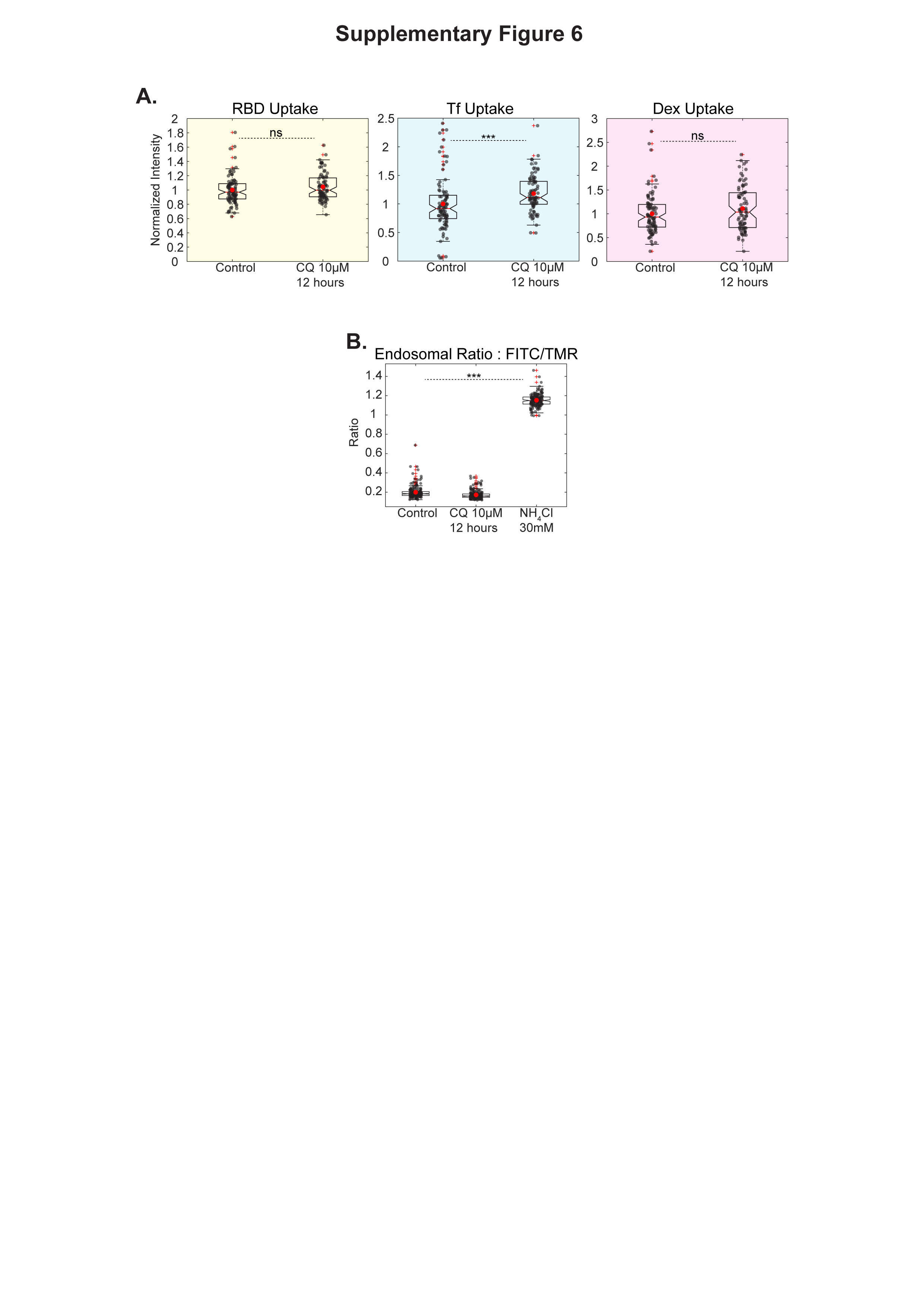

Supplement: S6 Fig — A, B: Using AGS cells treated with Control or CQ for 12 hours, RBD, dextran and transferrin uptake experiment (A) and FITC/TMR endosomal ratio estimation experiment (B) was conducted. Quantification in G and H show that RBD uptake, dextran uptake and FITC/TMR endosomal ratio are unaffected by long term treatment with CQ. Number of cells > 80 for each condition. Data representation is as described in Fig 1. (TIF) [file ppat.1009706.s006.tif]

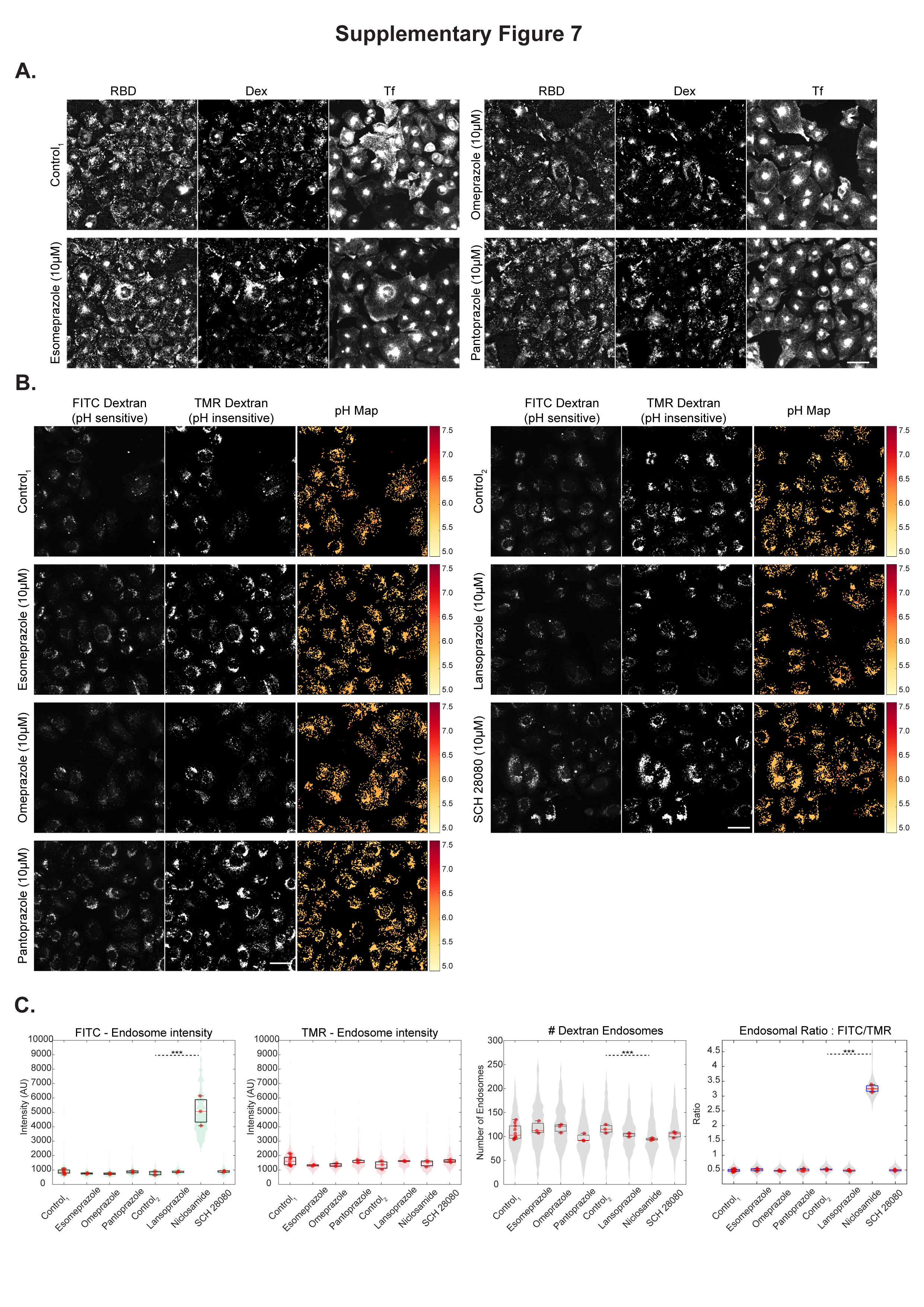

Supplement: S7 Fig — A: For the experiment described in Figs 6B and 6C, images are shown in 6B and S7A and quantification in Fig 6C. p-value table is indicated in S1 Table. B, C: For the experiment described in Fig 6D and 6E, images including pH maps are shown in Figs 6D and S7B and quantification in Figs 6E and S7C. FITC and TMR endosomal intensities and numbers of endosomes are quantified in S7C. Niclosamide increases FITC intensity, reduces numbers of endosomes and has minimal effect on TMR intensity. p-value table is indicated in S1 Table. (TIF) [file ppat.1009706.s007.tif]

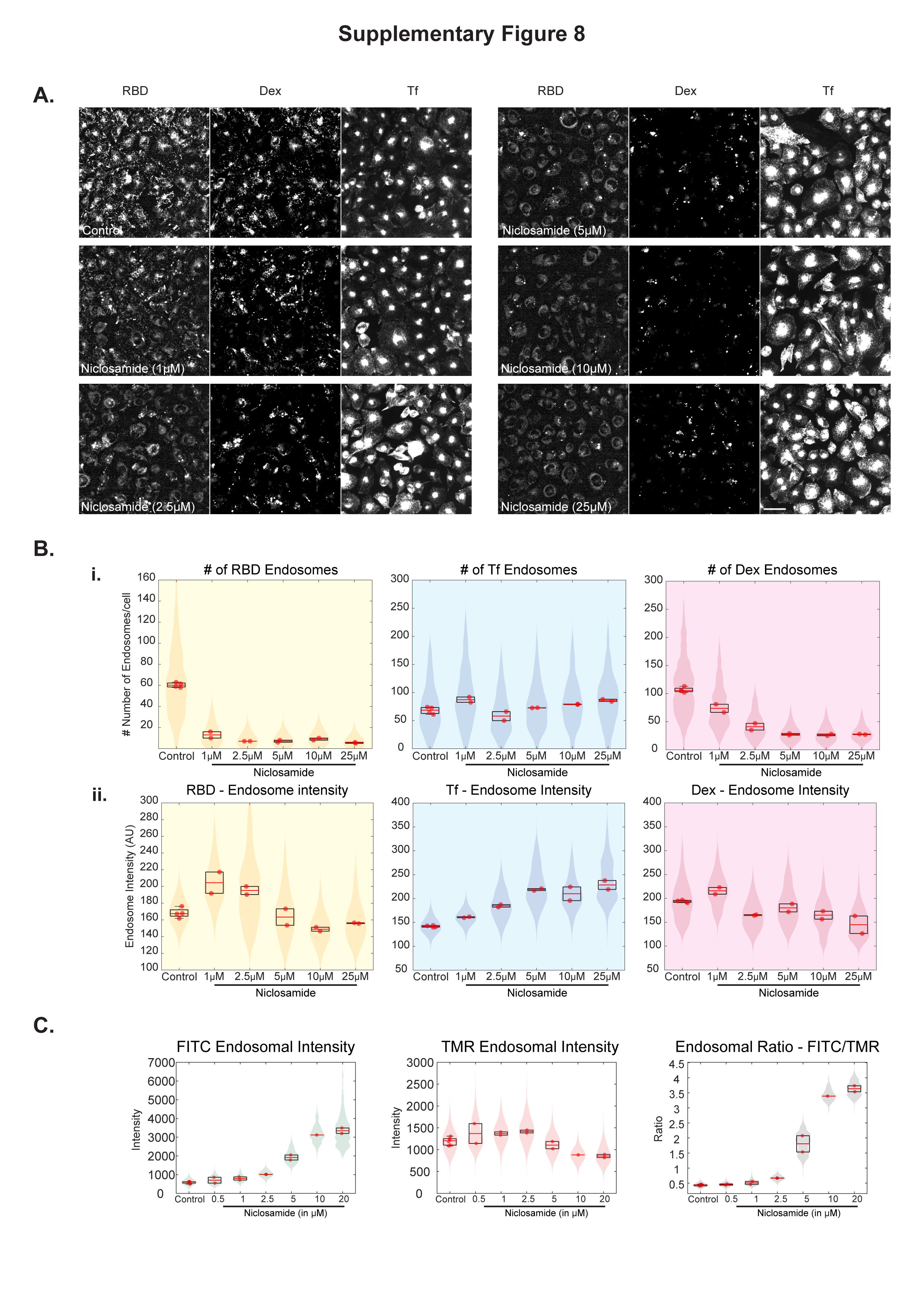

Supplement: S8 Fig — A, B: For the endocytic assay experiment described in Fig 7A, images are shown in S8A Fig and quantification in Figs 7A and S8B, with total cell mean intensity shown in Fig 7A, number of endosomes shown in S8B(i) Fig and intensity per-endosome shown in S8B(ii) Fig. The number of RBD endosomes and dextran endosomes decrease, while transferrin endosomal intensity increases with increasing concentrations of Niclosamide. p-value table is indicated in S1 Table. C: For the pH estimation assay described in Fig 7C and7D, quantification of endosomal FITC intensities, TMR intensities and FITC/TMR endosomal ratio is shown in S8C. A dose-dependent increase in FITC endosomal intensity, as well as ratio, is seen with increasing Niclosamide concentrations. p-value table is indicated in S1 Table. Data representation in B, C is as described in Fig 2. Scale bar shown in A is 40μm. (TIF) [file ppat.1009706.s008.tif]

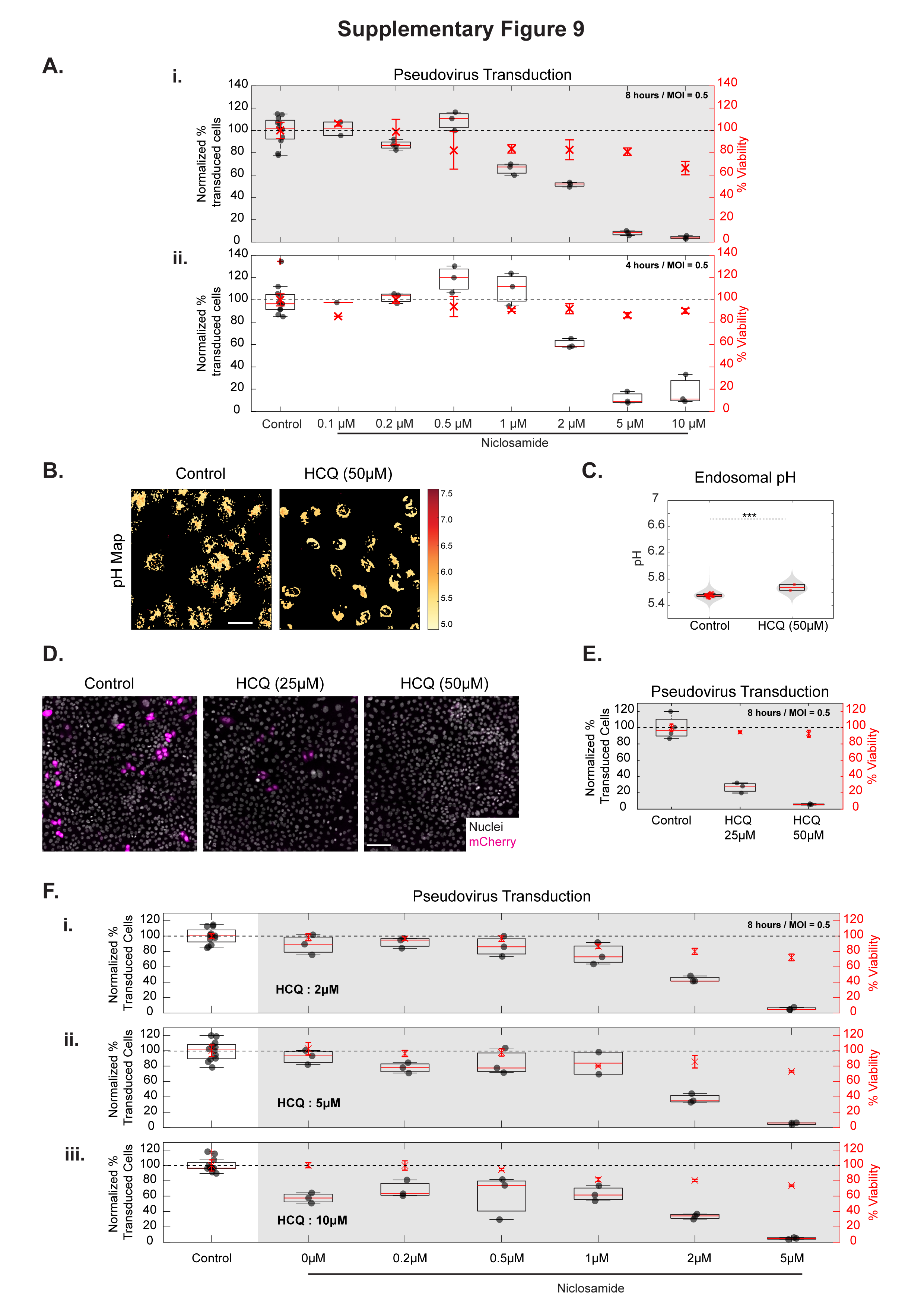

Supplement: S9 Fig — A: Quantification in A shows the normalized percentage transduction of Spike-pseudo virus across different concentrations of Niclosamide for incubation times of 8hours(i) and 4hours(ii) in AGS cells. Both the inhibitor and the virus were removed beyond the indicated times and cells were incubated with the continued presence of 100nM Niclosamide or 0.005% DMSO until termination. Images shown in Fig 7E and dose-response curve depicted in Fig 7F are related to the experiment in S9A(i). Number of repeats = 3 for each concentration of Niclosamide except 2 and 4 for 0.1μM and 0.2μM, respectively for the 8 hours set and 1 for 0.1μM Niclosamide in the 4 hours set. For the experiment in Fig 7G in AGS-ACE2 cells, number of repeats = 3 for each concentration of Niclosamide. p-value table is indicated in S1 Table. B, C: AGS cells pulsed with pH-sensitive (FITC) dextran for 2 hours and chased for 1 hour with Control or 50μM HCQ and imaged live. HCQ increases pH only slightly. pH maps are shown in B and quantification in C. Numbers of repeats: Control = 22, HCQ = 2. D, E: Images of AGS cells expressing the reporter mCherry protein upon transduction with Spike-pseudovirus in D and quantification in E show a dose-dependent reduction in transduction efficiency upon treatment with HCQ at the two concentrations tested compared to control (p-value < e-66 for HCQ 25μM, p-value < e-96 for HCQ 50μM). Number of repeats = 4 for control (0% DMSO) and 3 each for each concentration of HCQ. F: For the experiment described in Fig 7H, Quantification in S9F shows the normalized percentage transduction across indicated concentrations of Niclosamide in combination with indicated Hydroxychloroquine concentration of 2μM(i), 5μM(ii) and 10μM(iii). The percentage of cell viability for each condition is also indicated. Number of repeats = 2 for HCQ 5μM + Niclosamide 1μM combination and 3 each for all other combinations. p-value table is indicated in S1 Table. Data representation in A, E and F are as desc [file ppat.1009706.s009.tif]

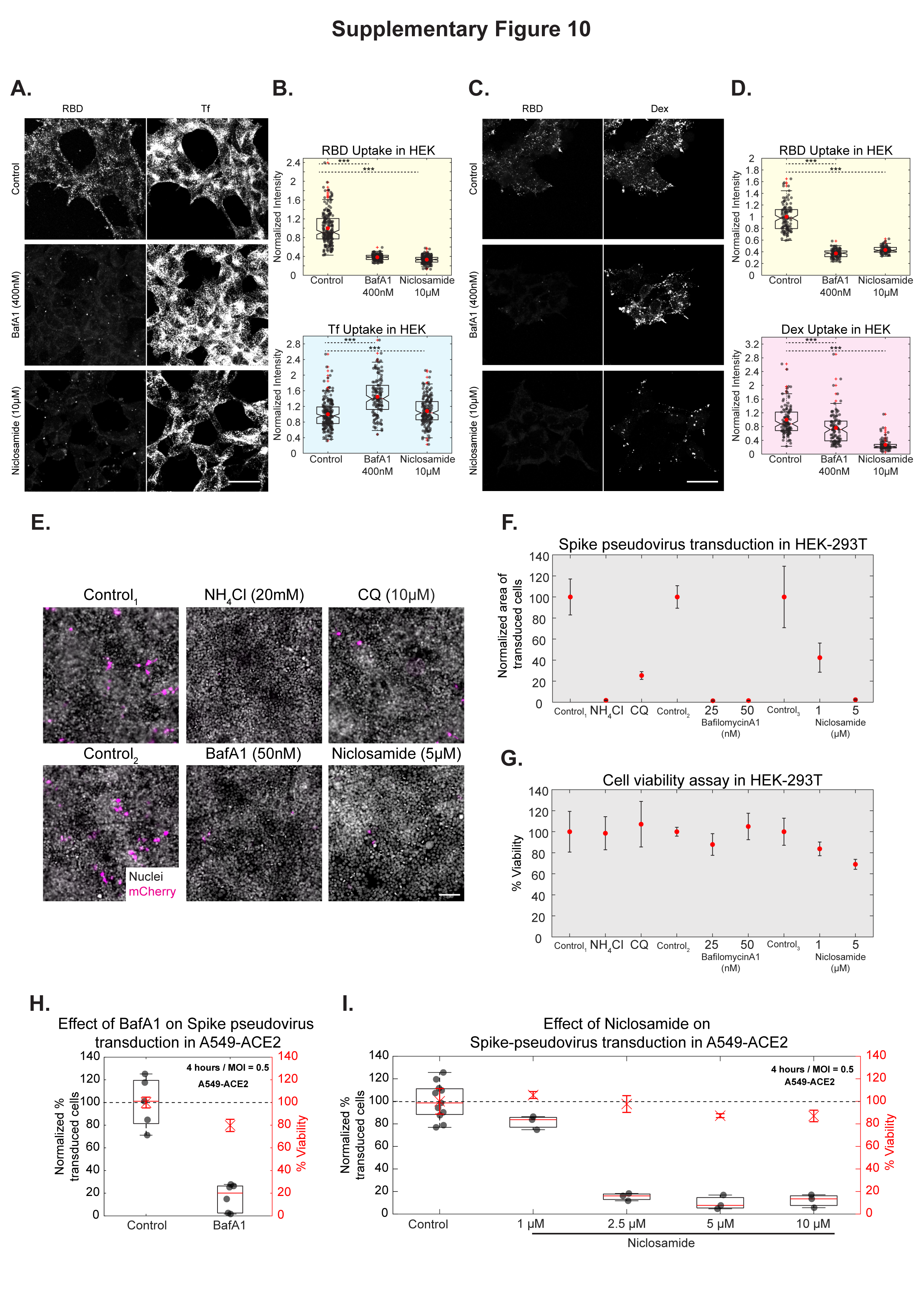

Supplement: S10 Fig — A-D: HEK-293T cells were treated with Control (0.4%DMSO), BafA1 400nM or Niclosamide 10μM for 30 minutes and pulsed with RBD and transferrin (A and B) or RBD and dextran (C and D) for 30 minutes with or without inhibitors. Images are shown in A and C, quantification is shown in B and D. RBD and dextran uptake is robustly reduced, while transferrin uptake increases upon treatment with BafA1 and Niclosamide. Number of cells ≥ 75 for each treatment. p-value table is indicated in S1 Table. E: Images show Spike-pseudovirus transduced mCherry positive HEK-293T cells in the presence of NH4Cl 20mM, CQ 10μM, BafA1 50nM and Niclosamide 5μM. F: Quantification of the normalized area of mCherry positive cells as a proxy for transduction, shows a reduction in transduction efficiency upon treatment with NH4Cl 20mM and CQ 10μM compared to 0% DMSO (Control1), 25nM and 50nM of BafA1 compared with 0.05%DMSO (Control2), 1μM and 5μM of Niclosamide compared to 0.1%DMSO (Control3). Number of repeats = 3 for each condition except 4 for 0%DMSO. The data is represented as mean +/- SD. G: Toxicity, as assessed by MTT based colorimetric assay, is represented as percentage viability of cells upon treatment with NH4Cl 20mM and CQ 10μM compared to 0% DMSO (Control1), 25nM and 50nM of BafA1 compared with 0.05%DMSO (Control2), 1μM and 5μM of Niclosamide compared to 0.1%DMSO (Control3). Number of repeats = 3 for each condition except 9 for 0%DMSO. The data is represented as mean +/- SD. H-I: Quantification of the mCherry positive cells in A549-ACE2 cells, as a proxy for transduction, shows a reduction in transduction efficiency upon treatment with 50nM of BafA1 in H and range of concentrations of Niclosamide in I (compared to respective DMSO controls). Data representation in B, D are as described in Fig 1 and H, I as in Fig 3. Scale bar: 40μm (A, C), 100μm (E). (TIF) [file ppat.1009706.s010.tif]

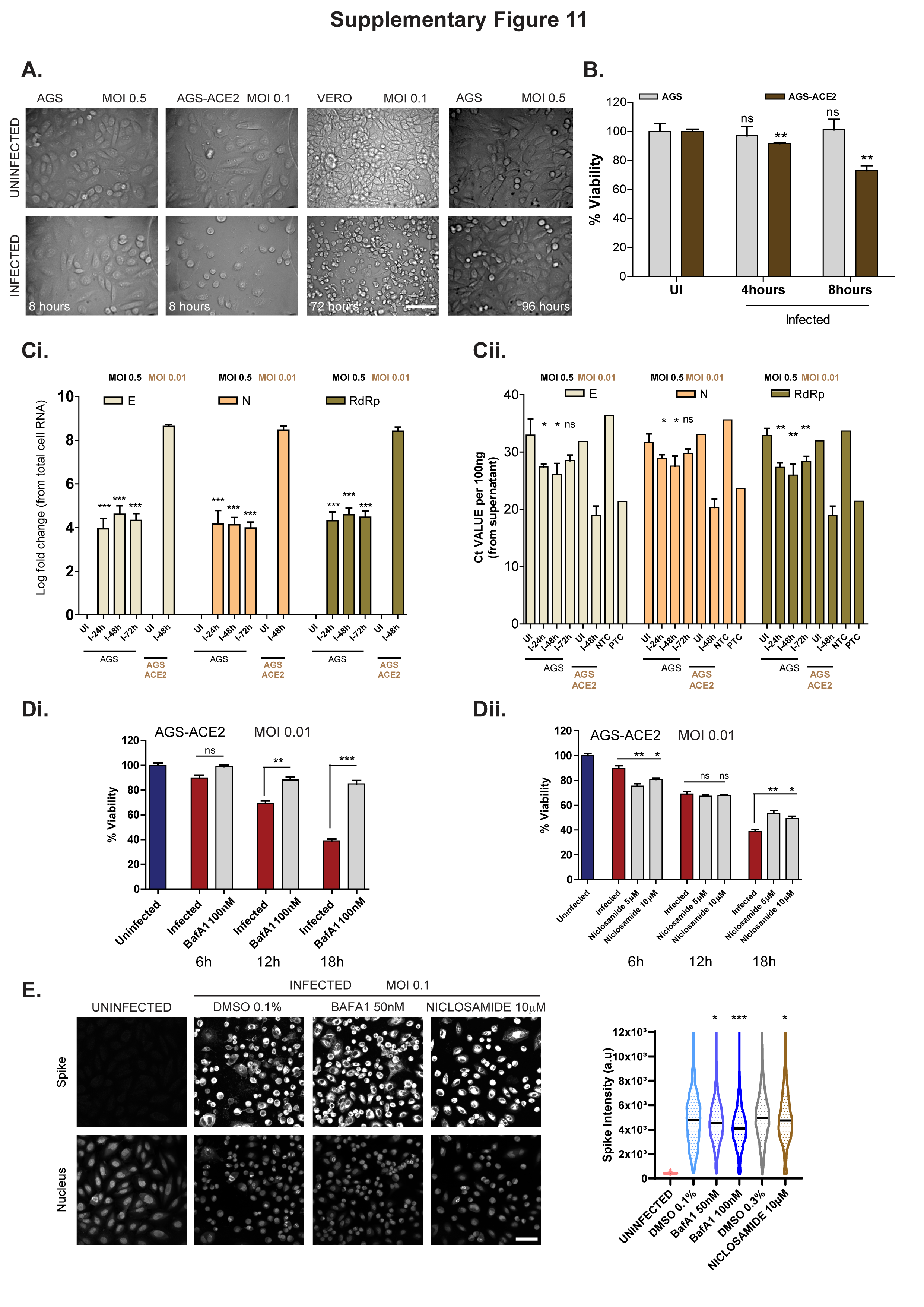

Supplement: S11 Fig — A: AGS, AGS-ACE2 and Vero cells were infected with viruses at indicated MOI for 8, 8 and 72 hours respectively. AGS cells were also infected with indicated MOI for 96 hours. Post infection, bright field images show cytopathic morphology in both AGS-ACE2 and Vero but not in AGS. Images shown are representative of multiple independent experiments. B: Evaluation of cell viability at early time points post infection in AGS and AGS-ACE2 cells. Cells were infected with viruses at indicated MOI for 4 hours or 8 hours. Cell viability, assessed using an ATP quantification assay, indicates cytopathic effects in AGS-ACE2 cells. Percentage viability relative to uninfected control is depicted. Number of repeats = 3 for each condition. C: Viral gene expression in AGS cell lysates (i) and supernatants (ii) as a function of duration of infection. AGS and AGS-ACE2 cells were infected with viruses at indicated MOIs for the specified time periods post infection. Expression is depicted as log fold change compared to uninfected cells in (i), raw Ct values of viral gene transcripts from culture supernatants in (ii). NTC: no transcript control; PTC: positive transcript control. Number of repeats = 3 (uninfected and infected AGS), 1 (uninfected AGS-ACE2) and 2 (infected AGS-ACE2). D: Effect of endosomal acidification inhibitors on SARS-CoV2 infection in AGS-ACE2 cells. Cells were pre-treated with control/inhibitor for 1 hour at indicated concentrations and infected with virus for 6 hours in the presence/absence of inhibitors. Viruses were then removed, and cells were further incubated for 0, 6 or 12 hours. Cells treated with Niclosamide were maintained at 1μM post infection. Upon termination, cell viability was assessed by ATP quantification assay. Number of repeats = 3 for each condition. E: Detection of SARS-CoV-2 Spike antigen in infected AGS-ACE2 cells. Cells were pre-treated with control/inhibitor for 1 hour followed by infection with viruses at indicated MOI for 30 minutes in the pre [file ppat.1009706.s011.tif]

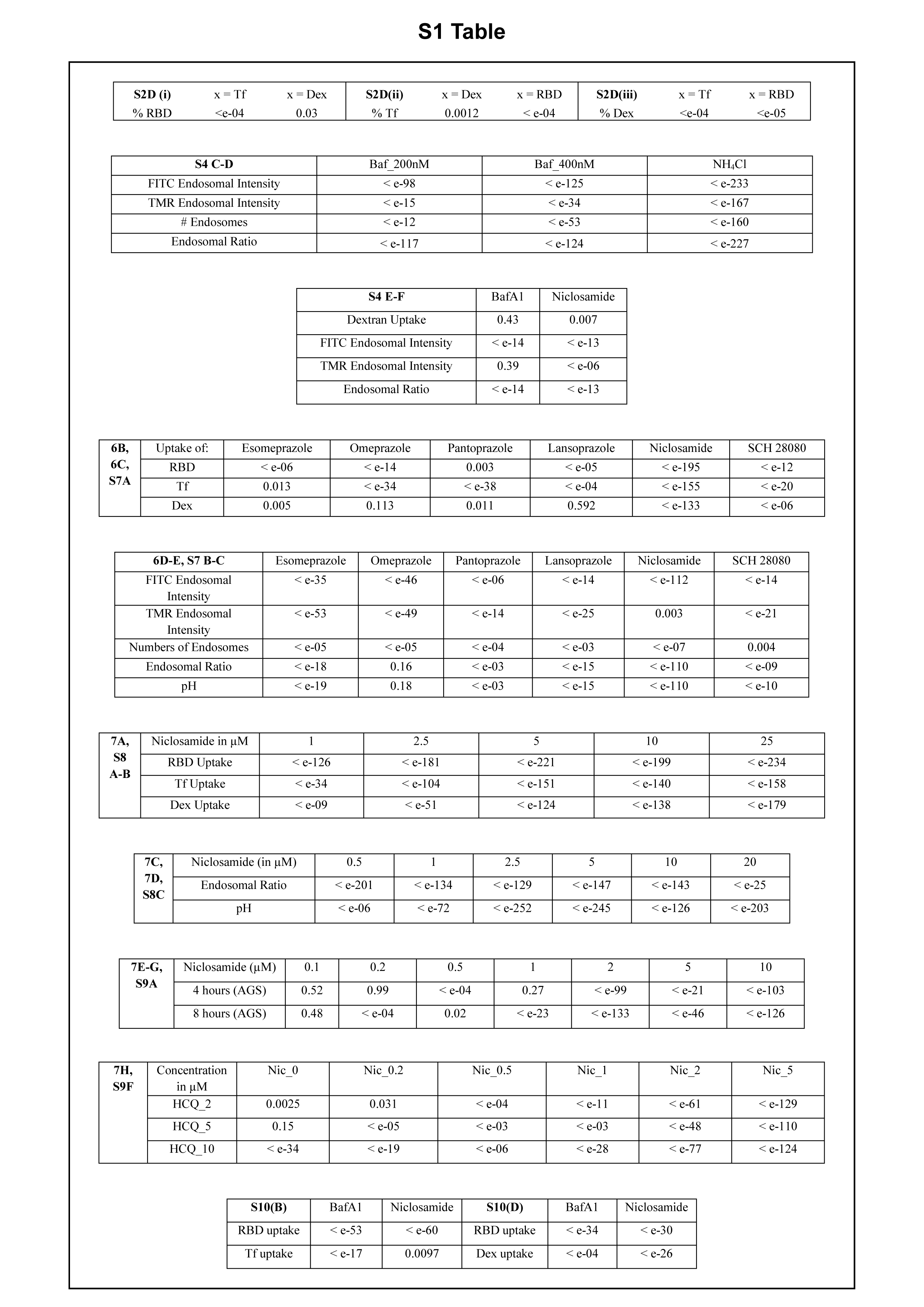

Supplement: S1 Table — Statistical tests between control and treatment were performed using Wilcoxon rank-sum test. (TIF) [file ppat.1009706.s012.tif]
