# Supplementary material for: Strategies to target SARS-CoV-2 entry and infection using dual mechanisms of inhibition by acidification inhibitors
Source: PLoS Pathog. 2021 Jul 12;17(7):e1009706. doi: 10.1371/journal.ppat.1009706 (PMC8297935; doi:10.1371/journal.ppat.1009706)
Supplement: S1 Text — (PDF) [file ppat.1009706.s013.pdf]

Strategies to target SARS-CoV-2 entry and infection using dual mechanisms of inhibition by acidification inhibitors

S1 Text: Supporting Methods

Cell lines, constructs, and antibodies:

Cell lines: AGS cells (Human adeno gastric carcinoma cells stably expressing human Folate Receptor) were maintained in HF12 media (HiMEDIA, India). HEK-293T, LentiX-293T, A549 and A549-ACE2 (BEI Resources, Cat# NR-53522) cells were maintained in DMEM-High Glucose (HiMEDIA, India). All culture media were supplemented with 10% FBS (16000044, Invitrogen), $NaHCO_3$ and L-Glutamine/ Penicillin/ Streptomycin solution (G1146, Sigma Aldrich). For AGS cells, the antibiotic hygromycin B (10687010, Invitrogen) was added at a concentration of 200µg/ml for selection during maintenance of cultures and the antibiotic-free medium was used for assays. AGS cells stably expressing ACE2 was prepared by transducing AGS cells with lentiviral particles made from second generation packaging system. These cells were maintained with 20µg/ml of puromycin (Sigma P8833) and hygromycin B. For assays with AGS-ACE2, puromycin containing media was used. A549-ACE2 were maintained with 2 µg/ml of puromycin. Vero cells (African green Monkey kidney cell line) were maintained in DMEM-High Glucose (Thermo Fischer, 11995065) supplemented with 10% heat inactivated FBS (16000-044, Thermo).

Constructs: pCAGGS-Secreted-RBD-6X-His (gift from Florian Krammer, Mt. Sinai), pCEP4-myc-ACE2 (gift from Erik Procko; Addgene plasmid #141185), pLenti-hACE2-Puro (gift from Raffaele De Francesco; Addgene plasmid #155295), pCDNA3.1 ACE2 GFP (gift from Utpal Pajvani; Addgene plasmid #154962), pTwist-EF1alpha-nCoV-2019-S-2xStrep (gift from Nevan Krogan, UCSF), pHR mCherry plasmid (gift from Minhaj Sirajuddin, inSTEM, India) and second-generation lentiviral helper plasmid psPAX2 (gift from Didier Trono; Addgene plasmid #12260).

Antibodies: Anti-human transferrin receptor antibody was purified from mouse hybridoma and labelled using NHS-ester chemistry. Anti-Myc Mouse mAb from CST (9B11 clone; Cat# 2276) was used to label myc-ACE2. Alexa Fluor 568 labelled goat anti-mouse secondary (115-005-071; Jackson Laboratory) was used to detect labelled Myc. Monoclonal Anti-Strep Tag antibody (clone GT517; Cat# SAB2702215) from Sigma-Aldrich and Goat anti-Mouse HRP secondary antibody (115-035-208; Jackson Laboratory) was used to identify the C-term Strep-tag on the Spike protein in the Spike-pseudotyped virus lysate western blot. Anti-ACE2 pAb from Sigma (SAB3500977) was used to detect ACE2 and anti-tubulin pAb from (Sigma, T3526) was used to detect Tubulin on western blot. Polyclonal Anti-SARS Coronavirus 2 spike Glycoprotein (IgG, Rabbit; BEI Resources, Cat# NR-52947) was used to label the spike protein of SARS-CoV-2. Alexa Fluor 647 labelled donkey anti Rabbit (Invitrogen, A13573) was used to detect the primary antibody.

Chemicals and reagents:

BafilomycinA1 (B1793 Sigma), NH_4Cl (A9434, Sigma), ML141 (4266, Tocris Bioscience), Chloroquine diphosphate (C6628, Sigma), Amiloride (A7410, Sigma), Atto 488 NHS Ester (41698; Sigma), TMR dextran (D1817, Thermo Scientific), Dextran 10kDa (D1860, Invitrogen), FITC Isomer-I (F1906, Molecular Probes), MTT (3-(4,5-Dimethylthiazol-2-yl)-2,5-diphenyltetrazolium bromide, M6494, Invitrogen), Hoechst (bisBenzimide trihydrochloride, H33342, Merck), Fugene 6 Transfection Reagent (E2692, Promega), Lipofectamine 3000 (Invitrogen), Lenti-X concentrator (631232, Takara Bio), 10X lysis buffer (9803, CST), Micro BCA™ Protein Assay Kit (23235; Thermo Scientific), PVDF

membrane (Immobilon-P, IPVH00010, Millipore), SuperSignal™ West Pico Chemiluminescent Substrate (34080, Thermo Scientific), TRI Reagent (AM9738, Invitrogen), RevertAid Reverse Transcriptase (EP0441, Thermo Scientific), Maxima SYBR-Green/ROX (K0221, Thermo Scientific), CellTiter- Glo Luminescent Cell Viability Assay (G7151, ProtoGen), Crystal violet (CO775, Sigma), VIRALDTECT-II multiplex real time PCR kit (G2MO20220, Genes2me) and Cell Mask Blue (H32720, Invitrogen). Hydroxychloroquine was provided by Mylan Laboratories.

Preparation of RBD:

RBD purification was optimized using a protocol from Krammer's Laboratory [1]. The procedure is briefly described in S1A Fig. HEK-293T cells were seeded in T75 flasks, grown up to 60% confluency and transfected with secreted RBD construct using Lipofectamine 3000. Cells were incubated with serum-free media during the first 4 hours of transfection and then changed to medium containing 2% serum. Cells were grown under these conditions for 40-48 hours, after which the media with secreted RBD was collected. The same cells were further incubated with fresh 2% serum-containing media for another 40-48 hours and the media containing secreted RBD was collected at the end of the incubation time. The collected media was filtered using a 0.45-micron filter to remove cell debris and other contaminants. RBD was purified by immobilized metal ion affinity chromatography (IMAC) over a 5-mL HiTrap His column (GE Healthcare Life Sciences) with step elution using buffers containing 0-500mM Imidazole. RBD containing fractions was found to be eluted within the range of 150 - 300mM Imidazole-containing elution buffers (S1B Fig). These fractions containing RBD were pooled and concentrated using 3kDa filters (Amicon). The concentrated filtrate was loaded onto HiLoad® 16/600 Superdex® 200 (GE Healthcare Life Sciences) gel filtration column and eluted using 1X PBS to separate RBD from serum albumin. The RBD-containing fractions were collected and concentrated using Centrivap vacuum concentrator or 3 kDa filters (Amicon). To label RBD using NHS ester chemistry, a 10-fold molar excess of Atto-488 NHS ester (Sigma) was incubated with RBD at pH 8.5 for an hour at room temperature. Labelled protein was separated from free dye using gravity flow-based size exclusion chromatography (Thermo Scientific).

Preparation of Spike-pseudovirus particles and estimation of titre:

Spike-pseudovirus particles were generated in LentiX-293T cells by co-transfecting the lentiviral helper plasmid psPAX, the C-term 2X-Strep-tagged Spike-encoding pTwist plasmid and a lentiviral pHR mCherry reporter plasmid encoding soluble mCherry protein under the control of SFFV promoter (S5A Fig). Transfection was carried out using Lipofectamine 3000. The expression of the Spike protein in the pseudovirus particles was verified by a western blot using the antibody against the C-terminal Strep-tag of Spike, which revealed bands corresponding to both the S2 fragment as well as the full-length protein (S5B Fig). Generation of bald pseudoparticles was carried out using same method except Spike-encoding plasmid was omitted from the cocktail. The supernatants were collected at least 3 times post-transfection between 48hours-72 hours. The viral supernatants were pooled and concentrated using the Lenti-X concentrator following manufacturer's protocol. The concentrated virus was resuspended in a small volume of Opti-MEM® (Gibco®), aliquoted and stored in -80ºC. For determining titre, AGS cells were transduced at different dilutions of the pseudovirus preparation for 72hours. The number of mCherry positive cells/colonies in the entire area of a 96 well plate was counted, and titre was recorded as Transducing units per ml (TU/ml). All procedures were carried in containment in a BSL2 facility.

For evaluation of comparative infection by pseudoparticles expressing or lacking Spike protein, viral supernatants were prepared as described above and concentrated 80 times (80x). Both preparations were

serially diluted (5x, 2.5x, 1x, 0.5x and 0.25x) and added to cells. Post infection, cells were scored for expression of mCherry reporter (S5C Fig).

q-PCR:

Total RNA was isolated from different cell lines using TRI Reagent. 500ng of RNA was converted into cDNA using Revert Aid Superscript RT as per manufacturers' instructions. q-PCR for *ACE2* and *TMPRSS2* was performed using SYBR-Green kit. The expression levels were normalized to 18srRNA. Primers used is listed below:

Primer name	Sequence 5'-3'
ACE2 FP	ATCAGAGATCGGAAGAAGAAAA
ACE2 RP	AAAAGGAGGTCTGAACATCA
TMPRSS2 FP	TAGTGTCCCCAGCCTACCTC
TMPRSS2 RP	GCACCAAGGGCACTGTCTAT
18s rRNA FP	GTAACCCGTTGAACCCCATT
18s rRNA RP	CCATCCAATCGGTAGTAGCG

Western blotting:

Virus-containing culture media, from 48-72h post-transfection, were pooled as mentioned in the above section. The medium was briefly centrifuged to remove cell debris and filtered using a 0.45µm filter. Filtered supernatant was overlaid on freshly prepared sucrose solution layer (50mM Tris-HCl, pH 7.4, 100mM NaCl and 0.5mM EDTA; with 20% sucrose (w/w) at a 4:1 ratio (v/v) [2]) and centrifuged at 15000 g at 4ºC for 4h. The supernatant was carefully discarded and any remaining liquid was removed by gently inverting the tube onto a blotting paper. The pellet containing pseudovirus particles was gently resuspended in a minimum volume of PBS (pH 7.4) on ice. Pseudovirions were then lysed using 10X lysis buffer. Protein content was estimated using a Micro BCA™ Protein Assay Kit as per manufacturer's protocol. To validate the presence of spike protein, 20µg of this lysate was loaded onto a 10% SDS polyacrylamide gel, blotted on a PVDF membrane and probed with anti-Strep-tag primary antibody followed by HRP secondary antibody.

For ACE2 western blot, lysates were prepared using cell lysis buffer (50mM Tris-HCl (pH7.4), 150mM NaCl, 1mM PMSF, 1mM EDTA, protease inhibitor cocktail III, 1% Triton X-100, 1% sodium deoxycholate and 0.1% SDS). Protein content was estimated using Micro BCA™ Protein Assay Kit as per manufacturer's protocol. For detecting ACE2 levels, 40ug of total protein lysates were loaded onto a 10% SDS polyacrylamide gel, blotted on a PVDF membrane and probed with anti-ACE2 primary antibody followed by anti-rabbit HRP secondary antibody. Processed blots were stripped using a stripping buffer (1.5% glycine, 0.1% SDS and 1% Tween 20, pH 2.2) and reprobed with anti-tubulin primary antibody followed by anti-mouse HRP secondary antibody.

RBD competition assay:

An excess of 10^{14} molecules of monomeric RBD and trimeric RBD was used for competing 1500 pseudovirus particles. Pseudovirus particles are estimated to have $4.5*10^5$ binding sites, considering 100 spike trimer or 300 spike monomers per virus particle, using the reported numbers for SARS-CoV [3]. The pseudovirus with excess protein mixtures were incubated at 37 ºC for 15 minutes. Following this, the pseudoviruses mixtures were added to AGS cells for 8 hours. After this, the medium was removed, and cells were replenished with fresh medium (not containing RBD or pseudoviruses) for 48 hours and scored for mCherry reporter expression (S5E Fig).

Cell viability assay:

To check the toxicity of inhibitors in HEK-293Ts, cells were treated with specified concentrations of the inhibitors, similar to the format employed for transduction assays (except no pseudoviruses were added). At the end of the incubation period, cells were washed twice with serum-free medium and then incubated with MTT working solution (20µl of 5mg/ml MTT in serum-free medium + 80µl of growth medium) for 3 hours at 37°C. Following this, contents from all wells were removed completely and 100µl of DMSO was added to each well. Complete dissolution of crystals in DMSO was ensured. Optical densities were then recorded at 570nm. Wells containing no cells were used for background correction. Values corresponding to cells treated with respective vehicle controls were considered as 100%.

Isolation and Titre determination of SARS-CoV-2:

SARS-CoV-2 NR-52284 was obtained from BEI-Resources. The virus was propagated by infection in Vero cells. Infected cells were monitored daily for cytopathic effects (as a proxy for confirmation of SARS-CoV-2 replication) using phase contrast bright field microscopy. In addition, cell supernatants were collected every 24 hours for quantitative PCR with reverse transcription (qRT-PCR) for detection of viral E (envelope), N (nucleoprotein) and RdRp (RNA-dependent RNA polymerase) genes. For the propagation of virus, three passages were performed in Vero cells and viral titres were determined by plaque forming unit (pfu) assays using crystal violet agar method, as previously described [4]. Aliquots of SARS-CoV-2 in defined titres were stored at -80 °C.

Viral infection evaluation assays:

1. Cell viability assays

To assess the cell death caused by SARS-CoV-2 in infected cells, two different assays were used. The CellTiter Glo assay was performed following manufacturer's instructions. Briefly, CellTiter Glo reagent was added to wells at a 1:1 ratio of cell culture supernatant volume. Plates were incubated at room temperature for 10 minutes and luminescence was measured for 500ms using SpectraMax multimode reader. In the second method, infected wells were fixed with 4% para formaldehyde (PFA) for 1 hour at room temperature and stained with crystal violet (50ul of 0.5% of crystal violet to each well and incubated for 10-15 minutes). Plates were then washed 3-4 times with distilled water to remove excess crystal violet and dried. Dried plates were scanned, converted into grey scale images and pixel intensities of individual wells were quantified using a custom macro in Fiji.

2. Spike Immunostaining

Cells were fixed at indicated time points with 4% paraformaldehyde for 30 minutes and labelled with SARS-CoV-2 spike protein. 0.2% of triton-x-100 in 0.2% BSA was used to permeabilize the cells for 10 minutes at RT followed by blocking in 1% BSA for 30 minutes. The cells were incubated for 1 hour at room temperature with Polyclonal Anti-SARS-CoV-2 Spike (1:2000) in blocking buffer. After three washes with wash buffer, the cells were incubated with Alexa Fluor 647 labelled donkey anti Rabbit (1:500) for 1 hour at room temperature in blocking buffer. After four additional washes, wash buffer supplemented with 5uM Hoescht and 2µg/ml of Cell Mask Blue was added to the cells for at least 10 min followed by two washes before imaging. Confocal images were acquired at 40X magnification using the automated high content spinning disc imaging system, Phenix. Anaysis was done using an automated pipeline set up with Harmony software from Perkin Elmer. The pipeline involved flat filed

correction, maximum projection and cell segmentation based on intensity and size of stained nuclei and cell cytoplasm marked by Hoescht and cell mask blue respectively. Mean intensities for Spike protein were calculated per cell and per well by the pipeline.

3. qRT-PCR

At indicated time points, cell supernatant and lysates were harvested for quantitative RT-PCR. Briefly, RNA isolation was done using TRIzol reagent following manufacturer's protocol using Genes2me qPCR kit to quantify SARS-CoV-2 replication. Each 20 µL reaction mixture contained 10 µL of 2 × OneStep Master Mix, 1µL of Target primer/Probe mix, and 9 µL of extracted RNA as template. Reaction conditions is as follows: 55°C for 10 min for reverse transcription, 95°C for 3 min for denaturation, followed by 40 cycles of 95°C for 15 s and 55°C for 60 s.

High content screening and automated imaging methodologies:

For high-throughput endocytic and pH estimation assays, AGS cells were plated in optical bottom 96 or 384 well plates at ~8000 or ~2000 cells/well and processed 16 hours after seeding. Experiments were conducted as detailed in the main text Methods using an automated plate washer and a robotic arm.

Image segmentation and feature extraction:

The maximum projected images were corrected for the system background and uneven illumination. The background was measured from wells not containing cells and subtracted from the raw data. For each channel, the uneven excitation was estimated by averaging all the maximum projected images from a single day's experiment and applying a 2D Gaussian filter of 50. This filtering removed any potential low-intensity features. The average of all the pixel values was then normalised to 1 to generate an illumination profile image. All background-corrected images were divided by this estimated value of illumination.

For cell segmentation, the Hoechst-stained nuclei images were used to first identify cells using Otsu's method on log-scaled intensity images. Next, using Sobel edge detection on each endosome channel, a 2D Gaussian filter of 20 pixels together with the Otsu methods allowed for the generation of a binary cell mask that contained most of the cell body of each cell. This allowed us to determine a field averaged background value by measuring the median intensity of the pixels outside of the area occupied by cells for all channels. Finally, individual endosomes within a cell were identified by using the endosome channels together with Otsu's method using the expected endosome sizes between 1-15 pixel diameters. Having identified cells and individual endosomes, their mean intensities in all channels, as well as their size and shape, were determined.

Synthesis of Niclosamide:

Niclosamide was synthesized by coupling 5-chlorosalicylic acid with 2-chloro-4-nitroaniline in the presence of thionyl chloride.

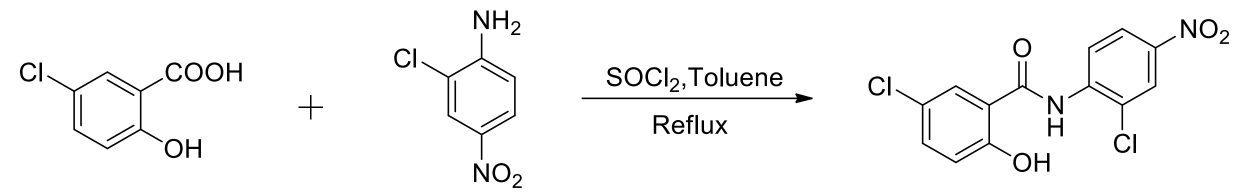

5-Chlorosalicylic acid 2-Chloro-4-nitroaniline Niclosamide

The 5-chlorosalicylic acid (1.72 g, 10 mmol) was suspended in 20 mL toluene. Thionyl chloride (160 µL, 2.2 mmol) was added and the reaction mixture was stirred at 110 °C for two hours. To this mixture was added 2-chloro-4-nitroaniline (1.14 g, 6.6 mmol) and the reaction was continued to stir at 110 °C for another 6 hours. The reaction mixture was allowed to cool to room temperature and kept overnight without disturbing. Niclosamide gets precipitated at the bottom of the RBF. The supernatant was decanted, the precipitate was washed with an excess quantity of water and the product was dried to get 1.8 g (83% yield) of niclosamide. The product was characterized by comparison of the spectral data with literature values [5]. Light yellow powder; m.p. 225-228 °C; HPLC: 98.9% purity; ^{1}H NMR (400 MHz, DMSO-d$_6$) δ 12.48 (s, 1H, OH), 11.34 (s, 1H, NH), 8.80 (d, *J* = 9.2 Hz, 1H), 8.44 (d, *J* = 1.6 Hz, 1H), 8.30 (dd, *J* = 8.4, 2.0 Hz, 1H), 7.98 (d, *J* = 2.0 Hz, 1H), 7.55 (dd, *J* = 8.2, 2.0 Hz, 1H), 7.10 (d, *J* = 8.8 Hz, 1H); ^{13}C NMR (100 MHz, DMSO-d$_6$) δ 162.56, 155.18, 142.54, 141.17, 134.03, 130.07, 124.78, 123.90, 123.78, 122.37, 120.70, 119.41, 119.16; IR (ν_{max}): 3488, 2921, 1679, 1607, 1348, 1195, 1124, 901 cm^{-1}; ESI-MS: *m/z* 325.05 [M-H]$^-$.

Spectral scans of Niclosamide:

^{1}H NMR:

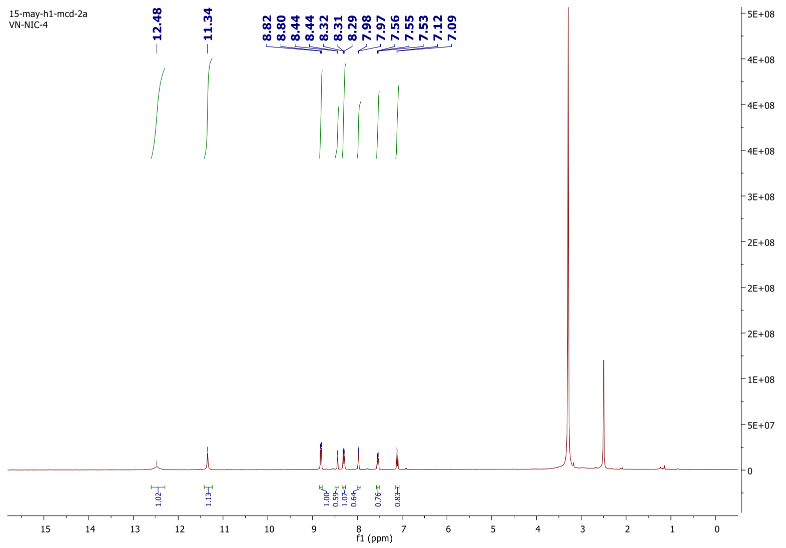

^{13}C NMR:

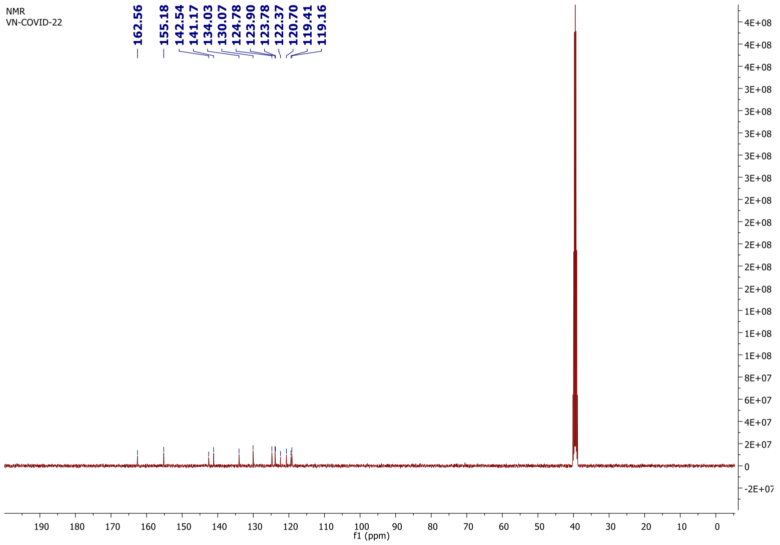

AN96, a stable analog of GBF1 inhibitor, LG186:

AN96(4-(2-(3,4,5-trimethoxybenzylidene)hydrazineyl)-5,6,7,8,9,10-hexahydrocycloocta[4,5]thieno[2,3-d]pyrimidine) was synthesized to create a more stable of analog of LG186[6,7], and this will be detailed elsewhere (Godbole et al., Manuscript in preparation).

LG-186 [6,7]

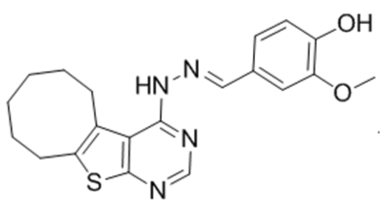

AN96

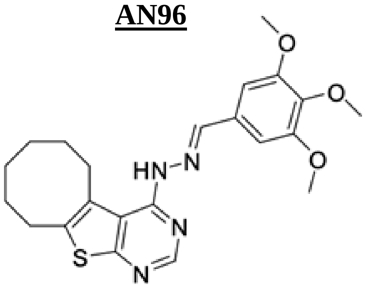

Extraction of Esomeprazole and Pantoprazole:

The commercially available tablets were dissolved in the aqueous phase and extracted with ethyl acetate (3x50mL). The organic layer was passed through anhydrous sodium sulphate, and the solvent was evaporated using Rotavapor. Recrystallization with hexane gave the pure compound in powder form. The purity of the drug has been confirmed using ^{1}H-NMR spectroscopy. Molecular weight of Esomeprazole and Pantoprazole is 345.4 g/mol and 383.4 g/mol, respectively.

Pantoprazole

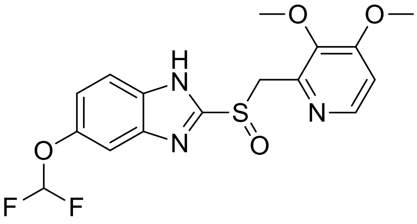

^{1}H NMR (600 MHz, DMSO-d6), δ (ppm) = 3.89 (s, 6H), 4.32 (d, J=12.9 Hz, 2H), 6.74 (dd, J=2.4 Hz, 2H),7.018 (s,1H), 7.07 (d, J=5.52 Hz, 2H), 7.24 (d, J=2.34 Hz, 1H), 7.45 (d, J=8.58 Hz, 1H).

^{13}C NMR (150 MHz, DMSO-d6), δ (ppm) = 56.36, 56.41, 61.42, 107.91, 108.32, 111.73, 116.26, 118.11, 119.65, 144.72, 144.80, 145.03, 146.27, 147.59.

Esomeprazole

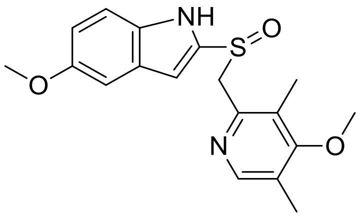

^{1}H NMR (600 MHz, DMSO-d6), δ (ppm) =2.21 (s, 6H), 3.68 (s, 6H), 4.40 (s, 2H),7.026 (s, 1H), 7.37 (d, J= 8.8 Hz, 1H), 8.25 (s, 1H).

References:

1. Stadlbauer, D. *et al.* SARS-CoV-2 Seroconversion in Humans: A Detailed Protocol for a Serological Assay, Antigen Production, and Test Setup. *Curr. Protoc. Microbiol.* **57**, (2020).

2. Jiang, W. *et al.* An optimized method for high-titer lentivirus preparations without ultracentrifugation. doi:10.1038/srep13875.

3. Bar-On, Y. M., Flamholz, A., Phillips, R. & Milo, R. SARS-CoV-2 (COVID-19) by the numbers. *Elife* **9**, (2020).

4. Mendoza, E. J., Manguiat, K., Wood, H. & Drebot, M. Two Detailed Plaque Assay Protocols for the Quantification of Infectious SARS-CoV-2. *Curr. Protoc. Microbiol.* **57**, 105 (2020).

5. Al-Hadiya, B. M. H. Niclosamide: Comprehensive Profile. *Profiles of Drug Substances, Excipients and Related Methodology* vol. 32 67–96 (2005).

6. Boal, F. *et al.* LG186: An Inhibitor of GBF1 Function that Causes Golgi Disassembly in Human and Canine Cells. *Traffic* **11**, 1537–1551 (2010).

7. Thottacherry, J. J. *et al.* Mechanochemical feedback control of dynamin independent endocytosis modulates membrane tension in adherent cells. *Nat. Commun.* **9**, 1–14 (2018).
