# Supplementary material for: Strategies to target SARS-CoV-2 entry and infection using dual mechanisms of inhibition by acidification inhibitors
Source: PLoS Pathog. 2021 Jul 12;17(7):e1009706. doi: 10.1371/journal.ppat.1009706 (PMC8297935; doi:10.1371/journal.ppat.1009706)
Supplement: Attachment — Submitted filename: 113001_1_rebuttal_2078109_qtlf3f.pdf [file ppat.1009706.s014.pdf]

## Point by Point Response to Reviewers

This document contains the responses to the reviewer comments for the Manuscript #RC-2020-00570. The reviewer's comment is marked in black and the author's response is in blue.

We thank all the reviewers for their constructive suggestions, criticisms and comments. We address them with new experiments and explanations listed below. Line numbers and Figure numbers are indicated according to the revised manuscript.

### *Reviewer #1 (Evidence, reproducibility, and clarity (Required)):*

The authors investigate in some detail how the SARS-CoV-2 spike protein or a Spike pseudotyped lentivirus are taken up by cells expressing the ACE2 receptor through the clathrin/dynamin-independent, pH-dependent CLIC/GEEC (CG) endocytic pathway. They show that the FDA-approved drug Niclosamide alone, or in combination with Hydroxychloroquine, interferes with Spike-dependent uptake.

While the authors go to great lengths in providing pharmacological evidence for their conclusions the manuscript has some major weaknesses.

**R1.1.** There are virtually no controls for their uptake studies in either ACE2-deficient cells or with an alternatively pseudotyped virus. This is particularly important because some of the conclusions derived from uptake of the Spike RBD don't apply to the Spike pseudotyped virus.

We thank this reviewer for emphasizing the importance of ACE2 in studying SARS-CoV-2 entry and infection. The Gastrointestinal tract comprises of cell-types differentially expressing ACE2: while esophagus and proximal stomach have undetectable ACE2, distal stomach, duodenum, colon and rectum express high levels [1]. Prompted by this, we examined the levels of ACE2 in AGS cells and found them to be deficient in protein expression (now added to **Figure 5A**). We compared both RBD uptake (now added to **Figure 5C-F, 7B**) and Spike-pseudovirus infection (now added to **Figure 5G-H, 7G**) in AGS and stable cell line of AGS overexpressing ACE2 (AGS-ACE2). Furthermore, we set up SARS-CoV-2 infection assays in both cell types (now added to **Figure 8, S11**). The conclusions of our study derived from these new experiments remain unchanged. Additionally, refer to the explanation provided for the query raised by Reviewer 2 (R2.2) on ACE2-dependence.

We have also conducted control experiments using VSV-G pseudotyped viruses in the AGS cells to compare the percentage of transduced cells with VSV-G and Spike pseudovirus. We observe that the transduction efficiency of VSV-G pseudovirus is significantly more than Spike-pseudovirus (now added to **Figure S5D**), as also observed by others [2]. Additionally, we have also confirmed that the VSV-G infection is sensitive to BafA1. This re-iterates that the mode of entry of VSV-G pseudovirus is via endocytic means, as also reported earlier [3,4].

The discrepancy between RBD uptake and Spike-pseudovirus infection pointed out as 'conclusions derived from uptake of the Spike RBD don't apply to the Spike pseudotyped virus' can be explained as below: Partial inhibition of uptake may not strongly manifest in our pseudovirus assay, as the read-out is all or none and is not sensitive to the number of virus particles entering the cells. Together with the results of BafA1 and $NH_4Cl$, our findings suggest that inhibitors that affect both RBD uptake and neutralize acidic endosomes could be one of the strategies used to impede Spike-pseudovirus

transduction. However, those which affect one or the other pathway may not be as effective at blocking virus transduction. This is also detailed in the main text (*Line numbers: 239-244*).

**R1.2.** The authors show that the inhibitor used to block the CG pathway, AN96, redirects RBD uptake towards a clathrin-dependent pathway. To my opinion this makes the inhibitor unsuitable for studies trying to distinguish clathrin-dependent and independent effects.

We would like to clarify this confusion. We observe that RBD uptake in AGS cells is primarily co-localized with the fluid phase dextran but not with endocytosed transferrin **(Figure 1)**. Previous experiments in the laboratory [5] have shown that the majority of the fluid phase uptake takes place via the CG pathway, independent of the clathrin-mediated endocytic pathway in many other cell types including AGS. Therefore, we concluded that RBD uptake in AGS cells is predominantly endocytosed via the CG pathway. Inhibiting the CG pathway (using AN96), we observe a significant reduction in RBD uptake (~25%). Further, the residual internalized RBD co-localizes much more with transferrin, internalized via the clathrin-mediated endocytic pathway (CME). This is similar to our observations with other bonafide CG cargoes (Examples: GPI-anchored proteins and fluid-phase dextran [5]) that are internalized via CME upon inhibition of the CG pathway. Thus, we postulate that in the absence/inhibition of CG, RBD enters cells via CME. There can be a plethora of binding receptors at the cell surface directing RBD uptake to either CG or CME. The use of inhibitors such as AN-96 allows us to clearly distinguish this possibility.

It is to be noted that transduction observed in the pseudovirus assays is at least two steps downstream of internalization: requiring conformational change in the Spike protein as well as fusion with the endosomal membrane and release of the virus nuclear material into the cytoplasm. BafA1 affects multiple trafficking steps relevant to transduction - first entry via the CG pathway (since CG uptake is blocked using specific acidification inhibitors as detailed here and [6]) and next by altering the acidic environment in all endosomes including late endosomes. On the other hand, AN96 only inhibits CG uptake. This could be a potential reason for BafA1 being a more potent inhibitor of pseudovirus transduction, compared to AN96. Absence of an effect on pseudovirus transduction upon AN-96 treatment suggests that virus entering the cells via CME could still lead to infection.

We further strengthened this point by studying the effect of BafA1 in cells overexpressing ACE2 (AGS-ACE2). In AGS-ACE2 cells, RBD enters via CME in addition to CG (now added to **Figure 4C, 4D**). In this scenario too, BafA1 inhibits pseudovirus infection (now added to **Figure 4G, 4H**).

*Specific comments:*

**R1.3.** Figures 1D, E: the effect of AN96 is comparable between RBD and Tf, taking into account that the y axes of the two graphs are different.

The reduction observed with RBD uptake and Tf is not comparable. Below, we mention the reduction (mean +/- sd) and the p-values of the Wilcoxon rank sum significance test (also mentioned in the main figure legends *line numbers: 16 and 17*) upon treatment with AN96.

RBD uptake reduces to 0.73 +/- 0.198 (p value < e-19) while the transferrin uptake is unaffected (0.92 +/- 0.4 with $p$ value of 0.02). The 27% reduction observed in RBD uptake is statistically significant.

In all quantified data the graphs should be displayed with the same y axis to facilitate a direct comparison.

All graphs show normalized uptake (normalized to the control). Different probes cannot be compared using similar scaling. To facilitate the comparison, the significance is indicated on all plots as well as the values are available in the figure legends.

**R1.4.** Figures 2E, F: the Tf control is missing.

The reviewer might be mistaken here. Figures 2E and 2F describe the 2nd assay (**Schematic in Figure 2C**). This assay is for evaluating the pH of endosomes. This assay utilizes 2 probes: pH sensitive FITC dextran and pH insensitive TMR dextran and there is no requirement for Transferrin here. Below is the section from the Methods describing the assay:

pH estimation assays:

For estimating the pH of late endosomes, cells were pulsed with pH-sensitive 10kDa FITC-dextran (1mg/ml) and pH-insensitive 10kDa TMR-dextran (1mg/ml) for 2 hours in serum-free media, chased for 1 hour in the presence of inhibitors or control and imaged live. The above pulse and chase times were chosen to allow the accumulation of labelled dextran in acidic late endosomal and lysosomal compartments (co-labelled with Lysotracker, data not shown). To estimate the endosomal pH, the ratio of FITC to TMR fluorescence was computed and compared to a pH calibration curve (Figures S4A-S4B) which was generated by equalizing the endosomal pH to that of an external buffer. After the pulse with FITC and TMR-dextran and chase, cells were incubated with 5µg/ml nigericin containing buffers of different pH for 10 minutes and imaged to evaluate FITC/TMR ratios for each pH.

For estimating the pH of late endosomes using the 488/458 excitation ratio of FITC-dextran (Figures 4E-4F), cells were pulsed with FITC-dextran at 1mg/ml for 2 hours, followed by chase in the presence or absence of inhibitors and imaged live.

For estimating the FITC/TMR ratio of early endosomes (Figures S4E-S4F), cells were incubated with pH-sensitive 10kDa FITC-dextran (1mg/ml) and pH-insensitive 10kDa TMR-dextran (1mg/ml) for 20 minutes, chased for 10 minutes and imaged live. Throughout the pulse and chase duration, the cells were incubated in serum-free media with control (0.2% DMSO) or BafA1 (400nM) or Niclosamide (10µM).

**R1.5.** Figure S3: there is by no means a linear relationship between dose and effect of the inhibitors. In contrast, the dose effect of BafA1 in e.g. Figs. 2D. E is very pronounced. How is this explained in terms of the role of pH?

The reviewer is correct here and we agree that there is not a linear relationship between RBD uptake and endosomal pH. As shown in the **Figure 2F**, for 200nM BafA1, there is a distribution of high pH endosomes as well as endosomes with pH similar to the average value. Whereas for 400nM BafA1, almost all endosomes show high pH **(Figure 2F)**. This is true for Niclosamide as well with 2.5µM Niclosamide showing more heterogeneous distribution of endosomes while higher concentrations of Niclosamide show that almost all endosomes have high pH (**Figure 7C**). The effect on RBD uptake is more pronounced with higher concentrations of BafA1 and Niclosamide. At this stage, we are not aware of the exact mechanism by which BafA1-sensitive vacuolar ATPases affect the CG pathway. However, we can only hypothesize that there could be a threshold effect of pH on CG endocytosis, since another agent, namely Niclosamide, whose target is not the vacuolar ATPases but it influences the pH in all endocytic compartments, has similar effects on RBD uptake.

**R1.6.** Figure S6D: the effect of competition is fairly small. Are there ACE2-independent uptake pathways?

We have now evaluated the effect of competition in the presence of excess monomeric or trimeric RBD in AGS cells, both of which show a significant reduction in infection (now added to **Figure S5E**). As expected, trimeric RBD shows more reduction in transduction efficiency compared to monomeric RBD. This confirms that the Spike-pseudovirus can be specifically competed with monomeric as well as trimeric RBD.

Additionally, refer to the explanation provided for the query raised by Reviewer 2 (R2.2) on ACE2 dependence.

Reviewer #1 (Significance (Required)):

For obvious reasons any gain of knowledge about SARS-CoV-2 is welcome these days and likely to arouse the interest of a larger than usual audience. The paper does not report anything entirely novel, but it emphasises the possibility to target viral uptake with FDA-approved drugs.

Reviewer expertise: innate immunity to pathogens

### *Reviewer #2 (Evidence, reproducibility and clarity (Required)):*

In this paper, the authors investigated the endocytic pathway leading to the internalization of the angiotensin converting enzyme 2 (ACE2), the receptor of SARS-CoV-2, when bound to its ligand, the receptor binding domain (RBD) of the spike protein of SARS-CoV-2. By using different cell biology approaches, they showed that the RBD of SARS-CoV-2 is internalized via a clathrin- and dynamin-independent, pH-dependent CLIC/GEEC endocytic pathway. Furthermore, they showed that endosomal acidification inhibitors Bafilomycin A1 and $NH_4Cl$, which inhibit the CLIC/GEEC endocytic pathway, blocked the internalization of the RBD. To further validate their observations in the context of a viral infection, they used retroviral pseudotypes harboring the spike protein of SARS-CoV-2. In this context, they confirmed that the acidification inhibitors also blocked viral infection. They also showed that chloroquine, a molecule shown to inhibit SARS-CoV-2 endosomal entry, does not affect RBD uptake and does not alter endosomal pH. However, it affected SARS-CoV-2 pseudoparticles entry. Finally, they also showed that niclosamide, an acidification inhibitor, blocks SARS-CoV-2 entry, suggesting the potential interest of this anti-parasitic FDA approved drug could be used in the treatment of SARS-CoV-2. The cell biology data showing RBD internalization with ACE2 receptor via a CLIC/GEEC endocytic pathway are potentially interesting after consolidation of the data. However, the virological part of the paper is not convincing.

We thank the reviewer for insights on the project and we have addressed concerns and suggestions with new experiments and explanations as listed.

**Major points:**

**R2.1-**There is an oversimplification in the presentation of SARS-CoV-2 entry in this manuscript. Coronaviruses are well known for their capacity to enter cells either by endocytosis or by direct fusion of their envelope at the plasma membrane of the target cells. In the case of SARS-CoV-2, there is growing evidence that the major route of entry is by fusion at the plasma membrane, at least in the respiratory tract which is the primary organ affected by this virus. Therefore, the idea of developing

inhibitors that block virus entry by endocytosis does not have a major medical interest. A good example is chloroquine which does not inhibit SARS-CoV-2 infection in lung derived cell lines or when TMPRSS2 is expressed in target cells (see Hoffmann et al., Nature, 2020, 585, 588) explaining the lack of effect of this drug in patients.

We agree with this reviewer that in lung epithelial cells studied such as Calu-3 which endogenously express higher levels of TMPRSS2, the process of viral entry may be predominantly via the cell surface route and is sensitive to inhibitors which affect TMPRSS2. However, this result does not imply or negate the possibility that viruses can enter cells via multiple pathways in any given cell type. CoVs are known to utilize both direct fusion route as well as endocytic routes for entry [7–11]. An understanding of which entry pathway is prevalent in each cell type is important as it allows better interpretation of cell-based drug-screens and translatability of cellular model of infection. Our study is focussed on the effects of acidification inhibitors in controlling the endocytic entry of SARS-CoV-2. The methods described in our study can be extended to primary cells that represent the more natural hosts for infection.

There is no indication whether or not the cells used in this study express TMPRSS2.

We measured the *TMPRSS2* transcript levels in the cell lines used in this study using qPCR and found the levels to be extremely low in all cell lines used in this study – AGS, A549 and HEK-293T (now added to **Figure 4B**).

There is also a confusion between internalization potentially mediated by ACE2 and the endosomal fusion process of the virus which is known to be pH-dependent. There is no evidence that the authors have tried to distinguish between these two processes with their pseudoparticles. It is indeed well known that NH4Cl, BafA1 and Niclosamide inhibit the fusion process by blocking conformational changes in the spike protein which is the motor of viral fusion.

We thank this reviewer for these criticisms, and they have driven further experimentation. Vacuolar ATPases (V-ATPases), which actively pump protons into the endocytic compartments [12], play a crucial role in the formation of CG endosomes as established using genetic and pharmacological perturbations [6,13]. By contrast, uptake through clathrin mediated endocytosis (CME) remains unaltered upon V-ATPase perturbation [6]. Our experiments indicate that BafA1 which inhibits V-ATPases has dual roles in controlling infection: inhibiting uptake via CG endocytosis as well as neutralizing the acidic compartments. AGS-ACE2 cell line provides an important tool to distinguish the effects brought about by acidification inhibitors on endocytosis and neutralization of acidic endosomes. This is because ACE2 biases RBD uptake towards CME (now added to **Figure 5C-5F**). Therefore, the effects brought about by BafA1 in this cell line will more predominantly be due to its neutralization role in the endo-lysosomal network.

We designed experiments to disentangle the possibilities of the involvement of BafA1 at different stages of the entry process in both AGS and AGS-ACE2 cells: by addition of BafA1 either before, during or after virus presentation (now added to **Figure 3F and 5G**). Our time-of-addition experiments revealed that the BafA1 sensitive step is during the virus presentation (~45 minutes) in both cell lines. However, pre-treatment with BafA1 or post-treatment with BafA1, even as early as 45 minutes after pseudovirus presentation, does not inhibit viral entry in both cell lines. This confirms that the effect of BafA1 is restricted to the early time points of entry. Furthermore, BafA1 inhibits infection in both AGS and AGS-ACE2 cells confirming that endosomal neutralization role of BafA1 is a necessary and sufficient step in controlling infection. Additionally, in cell lines with low ACE2 (like AGS and HEK293T), BafA1 also restricts infection by restricting entry via the CG pathway.

Finally, a final validation with an infectious SARS-CoV-2 virus would be welcome.

In agreement with the reviewer, we have now extended our observations with the clinical isolate of SARS-CoV-2 (now added to **Figure 8 and S11**). Briefly, both AGS and AGS-ACE2 cells were found permissive to infection by a SARS-CoV-2 clinical isolate with AGS-ACE2 being much more susceptible to cytopathy compared to AGS cells. BafA1 and Niclosamide could rescue the cytopathic effects brought about by SARS-CoV-2 in AGS-ACE2 and Vero cells (now added to **Figure 8D, S11D**).

Refer to section titled: *Bafilomycin and Niclosamide inhibit SARS-CoV-2 infection in AGS-ACE2 and Vero cells* (*Line numbers: 410-439*) in the Results section for more information.

**R2.2**-There is no clear demonstration that the uptake of RBD is mediated by ACE2. Other candidate receptors have also been proposed for SARS-CoV-2. A clear demonstration would be provided by a KO experiment showing that in the absence of endogenous expression of ACE2 there is no RBD internalization.

This comment is similar to the question raised by Reviewer 1 (R1.1 and R1.5) and we have addressed them altogether as detailed below:

We probed the levels of ACE2 in all the cell lines used in the study using western blot (now added to **Figure 5A**). While low levels of ACE2 was detected in HEK293T and A549 cells, we did not detect any ACE2 in AGS cells by western blot analysis (now added to **Figure 5A**). However, low expression of ACE2 transcripts was observed by qPCR in all the three cell types (now added to **Figure 5B**). Thus, AGS can be considered as a cell line with undetectable levels of endogenous ACE2. RBD uptake, pseudovirus transduction and infection by SARS-CoV-2 clinical isolates observed in AGS cells is likely independent of ACE2.

Towards understanding the specific effect of ACE2, a stable AGS cell line ectopically expressing ACE2 (referred to as AGS-ACE2) was generated and expression of ACE2 was confirmed using a western blot (now added to **Figure 5A**). On characterizing the RBD endocytic itinerary in AGS-ACE2 cells, we observed that in addition to trafficking via the CG pathway, RBD traffics via the CME (now added to **Figure 5C, 5D**). We further evaluated the effect of BafA1 on RBD and dextran endocytosis and observed a significant reduction in both RBD and dextran uptake in AGS and AGS-ACE2 cells (now added to **Figure 5E, 5F**). However, the absolute reduction of RBD uptake in AGS-ACE2 is not to the same extent as in AGS cells. This could be because BafA1 only affects the CG fraction of uptake and doesn't affect RBD entering via the CME.

Both AGS and AGS-ACE2 cells are permissive to infection by Spike-pseudovirus and infectious SARS-CoV-2 virus. AGS-ACE2 shows severe cytopathic effects and is more efficient at producing infectious virus upon infection with SARS-CoV-2 (now added to **Figure 8 and S11**). This result suggests that differences in susceptibility can be an outcome of interaction with different host factors and endocytic routes employed by the virus.

Refer to the new subsections in the Results section for more information:

- *ACE2 biases RBD uptake via the clathrin-mediated endocytic pathway* (*Line numbers: 269-291*)
- *Effects of BafA1 on RBD uptake and Spike-pseudovirus infection in AGS-ACE2* (*Line numbers: 292-317*)

*- Bafilomycin and Niclosamide inhibit SARS-CoV-2 infection in AGS-ACE2 and Vero cells* (*Line numbers: 410-439*)

There are also other questions that need to be answered concerning RBD and ACE2. Is the uptake of ACE2 constitutive or induced by RBD binding?

Since the AGS line does not have detectable levels of ACE2 protein, this question is not relevant to our findings.

Is the uptake of RBD receptor-mediated?

As included in the discussion and results (*Line numbers: 453-456 and 278-281*) - Although ACE2 has been identified as the receptor for SARS-CoV-2, other receptors are also being uncovered. These include Neuropilin [14,15], CD147 [16], Heparan Sulphate proteoglycans [17] and HDL scavenger receptors [18]. Additionally, the highly glycosylated nature of Spike protein could also confer the ability to interact with yet unidentified receptors. Preliminary attempts using heparin to compete for heparin-binding sites [16] or PIPLC to remove GPI-anchored proteins [34] showed only partial reduction in RBD uptake (data not shown). Further studies interrogating specific RBD-receptor interactions in AGS cells will be required to determine the exact binding mechanisms. However, this is beyond the scope of our current work.

**R2.3**-The experiments using pseudoparticles need proper controls. A comparison between HEK 293T cells and NIH 3T3 is not relevant. There is no evidence that the entry factors used by SARS-CoV-2 are similarly expressed in both cell line. Furthermore, NIH 3T3 cells are from mouse origin and it is well known that mouse ACE2 is not compatible with SARS-CoV-2 entry. better control would be to use the same pseudoparticles devoid of the spike protein. Furthermore, this can be consolidated by using neutralizing antibodies and/or anti-SARS2 antibodies to show the specificity of the entry process which is supposed to rely on the presence of SARS-CoV-2 spike.

We agree with the reviewer and have now conducted experiments with bald virus and compared the transduction efficiency with Spike-pseudovirus in AGS cells. Dilutions of the supernatant containing bald pseudoparticles showed no transduction, while similar dilutions with Spike-pseudovirus showed high levels of transduction in AGS (now added to **Figure S5C**). We also conducted additional control experiments (now added to **Figure S5D, S5E**):

a. Competition assays using monomeric and trimeric RBD in AGS cells: Both soluble and trimeric RBD show a significant reduction in Spike-pseudovirus infection confirming that the Spike-pseudovirus can be specifically competed with monomeric as well as trimeric RBD
b. Alternatively pseudotyped virus (VSV-G) in AGS cells: Transduction efficiency of VSV-G pseudovirus is significantly more than Spike-pseudovirus, as also observed by others [2]

All these experiments show the specificity of the Spike-pseudovirus.

**Minor points:**

**R2.4**-The title of the paper does not really reflect the work presented. It should therefore be modified. This is now modified to *"Strategies to target SARS-CoV-2 entry and infection using dual mechanisms of inhibition by acidification inhibitors"*.

**R2.5**-A positive control is needed for the amiloride experiment (Fig S2G - S2H) This is now included in **Figure S2I**. On using PMA to induce macropinocytosis, an increase in dextran uptake was observed.

This increase was inhibited on treating cells with PMA and Amiloride. Cells treated with Amiloride only or PMA with Amiloride had similar dextran uptake as control cells confirming the functionality of the Amiloride used in the study.

**R2.6**-The authors should use the internationally recognized abbreviation for the coronaviruses described in the paper: SARS-CoV-2, HCoV-229E, MERS-CoV, HCoV-OC43, ...: This has been corrected.

**R2.7**-The authors need to double-check when they quote the figures in the text:

Line 264: Figure 3D and 3E should be Figures 3F and 3G

Line 275: Figures 3F and 3G should be Figures 3H and 3I

Line 303: Figure 5E should be Figure 4E

Line 304: Figure 5D should be Figure 4D

We have cross-verified that all the figure number references in the text match the correct figure panels. Some figures have been modified and care has been taken to refer to the correct figure panel in the revised manuscript.

**R2.8**-Line S263: colony-forming units/ml (CFUs/ml) is not appropriate. It is normally used for bacteria!

Our titre estimation protocol involved infecting serial dilutions of the pseudovirus preparation and scoring for mCherry reporter expression after 48 hours. Using image-segmentation pipelines, we determined the number of mCherry-positive cells/colonies formed per dilution and recorded the titre as Colony forming units per ml. However, we have now changed our metric to Transducing units per ml (TU/ml). The method is detailed in Supplementary text (*Line number: S309*).

Reviewer #2 (Significance (Required)):

This is a cell biology work in which the authors have tried to include some SARS-CoV-2 virology which could potentially sell the paper to a larger audience. However, the virological part of the paper is not convincing probably due to the lack of competence of the team in the biology of viruses.

### *Reviewer #3 (Evidence, reproducibility and clarity (Required)):*

**Summary of findings**

In the first part, the manuscript describes a very interesting "detective work" to decipher the major, receptor-mediated mechanism of entry of the SARS-CoV2 virus. The experimental plan progresses step-by-step to confirm that the major receptor of the Spike is ACE2, even though the manuscript also confirms that additional (co)receptors contribute to uptake, before it rigorously determines that the CG pathway is the main route of entry. The study robustly demonstrates that this pathway is responsible for uptake, using a variety of cargoes, from the simplest (the RBD domain of Spike) to the more complex (pseudo viruses with the Spike protein). In addition, the authors provide strong, statistically significant and therefore robust evidence that this is valid in two different cell lines of human origin.

In the second part, the study takes advantage of the reporters of uptake and infection established in the

first part to conduct a screen for anti-infective compounds. Beside confirming the activity of some expected CG inhibitors, the authors take the well-trodden but perfectly appropriate path of drug repurposing. This strategy leads to the (re)discovery of niclosamide, a potent proton ionophore, as a CG inhibitor and promising lead for development of a SARS-CoV2 uptake inhibitor. More surprisingly, the authors also bring interesting findings on chloroquine, which apparently fails the test as acidification inhibitor, but nevertheless is shown to additively function with niclosamide as SARS-CoV2 uptake and infection inhibitor, though via an unknown mechanism.

The manuscript is very well written, the cartoons introduced in the figures to illustrate the experimental design are very helpful, and the details given in text, legends and M&M allow to really grasp how the experiments are conducted. The experimental strategies are often very complex, but absolutely well thought through, the reproducibility and quantifications are state of the art. Overall, a solid study with convincing findings, with data reasonable interpreted and placed in their context, with ample and adequate reference to others' work.

We thank the reviewer for the motivating comments and for reading through the work completely.

**Major criticisms**

My only, but possibly serious reservation concerns the cell type used for the various assays. I perfectly understand that one has to use model systems that allow robust monitoring and quantitation, and therefore, have to be amenable to a variety of cellular and molecular methodologies. But I do not really follow the logic of using adenocarcinoma gastric cells (and even worse for HEK-293T cells). I am not a specialist, but there are for sure lung epithelial cell lines and explant systems that would allow to confirm the most important results in a relevant system. For example, some colleagues of mine use commercially available Human Airway Epithelia reconstituted in well formats, which are routinely used for SARS-CoV2 testing (sorry for the advertisement, but check Epithelix MucilAir and SmallAir products at https://www.epithelix.com/news/?page=1#448).

We hope our study paves way for more investigations in primary cells that represent the more natural hosts for SARS-CoV-2 infection. There is growing evidence for the involvement of the gastrointestinal tract in SARS-CoV-2 infection [20]. Even though respiratory symptoms dominate the clinical presentation of COVID-19, a subset of patients also face gastrointestinal symptoms [21,22]. In vitro model systems such as Caco-2 cells (human adenocarcinoma colorectal cells) as well as enterocytes of human small intestine organoids are readily infected by SARS-CoV-2 [8,23]. The AGS cells, derived from stomach adenocarcinoma, provide a useful model to study virus infection in the context of gastrointestinal manifestations of SARS-CoV-2. We have evaluated infectivity of AGS and AGS-ACE2 using clinical isolate of SARS-CoV-2 and we observe that these cells are permissive to infection (now added to **Figure 8 and S11**). BafA1 and Niclosamide rescues cells from cytopathic effects brought about by infectious SARS-CoV-2 in both AGS-ACE2 and Vero cells.

As suggested, we validated our key results: the effects of BafA1 and dose dependent effect of Niclosamide on Spike-pseudovirus infection in human adenocarcinoma basal epithelial cells overexpressing ACE2 (A549-ACE2 cell line). Both these inhibitors abrogated Spike-pseudovirus infection (**now added to Figure S10 H-I**).

Reviewer #3 (Significance (Required)):

**Significance**

I am not a virologist and even less an epidemiologist, but the present work cannot be more timely, in the light of the preoccupying COVID-19 pandemics. In that context, and contrary to a very large proportion of the zillion publications on the topic, the present study has two fundamental strengths. First, it is conducted by world specialists in endocytosis and the methodology used to determine that CG is the major uptake pathway is most convincing. Based on this fundamental finding, the second strength is that the compound screens are performed with the necessary rigour and reproducibility, which is a prerequisite to advance from hits to lead. Now, whether the findings are significant enough, and obtained in a relevant-enough cellular model system to warrant progress towards tailoring a novel therapy to fight the pandemics still remains to be shown. But in any case, these robust and significant data have to be brought to light and shared with the scientific community at large.

References:


1. Hikmet, F. *et al.* The protein expression profile of ACE2 in human tissues. *Mol. Syst. Biol.* **16**, e9610 (2020).

2. Ou, X. *et al.* Characterization of spike glycoprotein of SARS-CoV-2 on virus entry and its immune cross-reactivity with SARS-CoV. *Nat. Commun.* **11**, (2020).

3. Sun, X., Yau, V. K., Briggs, B. J. & Whittaker, G. R. Role of clathrin-mediated endocytosis during vesicular stomatitis virus entry into host cells. *Virology* **338**, 53–60 (2005).

4. Johannsdottir, H. K., Mancini, R., Kartenbeck, J., Amato, L. & Helenius, A. Host Cell Factors and Functions Involved in Vesicular Stomatitis Virus Entry. *J. Virol.* **83**, 440–453 (2009).

5. Sathe, M. *et al.* Small GTPases and BAR domain proteins regulate branched actin polymerisation for clathrin and dynamin-independent endocytosis. *Nat. Commun.* **9**, 1–16 (2018).

6. Gupta, G. D. *et al.* Population distribution analyses reveal a hierarchy of molecular players underlying parallel endocytic pathways. *PLoS One* **9**, e100554 (2014).

7. Glowacka, I. *et al.* Evidence that TMPRSS2 Activates the Severe Acute Respiratory Syndrome Coronavirus Spike Protein for Membrane Fusion and Reduces Viral Control by the Humoral Immune Response. *J. Virol.* **85**, 4122–4134 (2011).

8. Hoffmann, M. *et al.* SARS-CoV-2 Cell Entry Depends on ACE2 and TMPRSS2 and Is Blocked by a Clinically Proven Protease Inhibitor. *Cell* **181**, 271-280.e8 (2020).

9. Simmons, G. *et al.* Characterization of severe acute respiratory syndrome-associated coronavirus (SARS-CoV) spike glycoprotein-mediated viral entry. *Proc. Natl. Acad. Sci. U. S. A.* **101**, 4240–4245 (2004).

10. Simmons, G. *et al.* Inhibitors of cathepsin L prevent severe acute respiratory syndrome coronavirus entry. *Proc. Natl. Acad. Sci.* **102**, 11876–11881 (2005).

11. Huang, I. C. *et al.* SARS coronavirus, but not human coronavirus NL63, utilizes cathepsin L to infect ACE2-expressing cells. *J. Biol. Chem.* **281**, 3198–3203 (2006).

12. Forgac, M. Structure, function and regulation of the vacuolar ($H^+$)-ATPases. *FEBS Lett.* **440**, 258–263 (1998).

13. Dey, G. *et al.* Exploiting cell-to-cell variability to detect cellular perturbations. *PLoS One* **9**, e90540 (2014).

14. Cantuti-Castelvetri, L. *et al.* Neuropilin-1 facilitates SARS-CoV-2 cell entry and infectivity. *Science (80-. ).* **370**, eabd2985 (2020).

15. Daly, J. L. *et al.* Neuropilin-1 is a host factor for SARS-CoV-2 infection. *Science (80-. ).* **370**, eabd3072 (2020).

16. Wang, K. *et al.* SARS-CoV-2 invades host cells via a novel route: CD147-spike protein. (2020) doi:10.1101/2020.03.14.988345.

17. Clausen, T. M. *et al.* SARS-CoV-2 Infection Depends on Cellular Heparan Sulfate and ACE2. *Cell* **183**, 1043-1057.e15 (2020).

18. Wei, C. *et al.* HDL-scavenger receptor B type 1 facilitates SARS-CoV-2 entry. *Nat. Metab.* (2020) doi:10.1038/s42255-020-00324-0.

19. Sabharanjak, S., Sharma, P., Parton, R. G. & Mayor, S. GPI-anchored proteins are delivered to recycling endosomes via a distinct cdc42-regulated, clathrin-independent pinocytic pathway. *Dev. Cell* **2**, 411–23 (2002).

20. Xu, J. *et al.* Digestive symptoms of COVID-19 and expression of ACE2 in digestive tract organs. *Cell Death Discov.* **6**, 1–8 (2020).

21. Gu, J., Han, B. & Wang, J. COVID-19: Gastrointestinal Manifestations and Potential Fecal–Oral Transmission. *Gastroenterology* vol. 158 1518–1519 (2020).

22. Cholankeril, G. *et al.* High Prevalence of Concurrent Gastrointestinal Manifestations in Patients With Severe Acute Respiratory Syndrome Coronavirus 2: Early Experience From California. *Gastroenterology* **159**, 775–777 (2020).

23. Lamers, M. M. *et al.* SARS-CoV-2 productively infects human gut enterocytes. *Science (80-. ).* **369**, 50–54 (2020).
